# A novel definition and treatment of hyperinflammation in COVID-19 based on purinergic signalling

**DOI:** 10.1007/s11302-021-09814-6

**Published:** 2021-11-10

**Authors:** Djo Hasan, Atsuko Shono, Coenraad K. van Kalken, Peter J. van der Spek, Eric P. Krenning, Toru Kotani

**Affiliations:** 1 Kasterlee, Belgium; 2 Department of Anaesthesiology and Critical Care Medicine, School of Medicine, Showa University, Tokyo, 142-8666 Japan; 3 Hoorn, The Netherlands; 4 Department of Pathology & Clinical Bioinformatics, Erasmus MC, Erasmus Universiteit Rotterdam, 3015 CE Rotterdam, The Netherlands; 5 Rotterdam, The Netherlands

**Keywords:** COVID-19, P2X7 receptor antagonist, Lidocaine base, Hyperinflammation, Cytokine storm, Immune paralysis

## Abstract

Hyperinflammation plays an important role in severe and critical COVID-19. Using inconsistent criteria, many researchers define hyperinflammation as a form of very severe inflammation with cytokine storm. Therefore, COVID-19 patients are treated with anti-inflammatory drugs. These drugs appear to be less efficacious than expected and are sometimes accompanied by serious adverse effects. SARS-CoV-2 promotes cellular ATP release. Increased levels of extracellular ATP activate the purinergic receptors of the immune cells initiating the physiologic pro-inflammatory immune response. Persisting viral infection drives the ATP release even further leading to the activation of the P2X7 purinergic receptors (P2X7Rs) and a severe yet physiologic inflammation. Disease progression promotes prolonged vigorous activation of the P2X7R causing cell death and uncontrolled ATP release leading to cytokine storm and desensitisation of all other purinergic receptors of the immune cells. This results in immune paralysis with co-infections or secondary infections. We refer to this pathologic condition as hyperinflammation. The readily available and affordable P2X7R antagonist lidocaine can abrogate hyperinflammation and restore the normal immune function. The issue is that the half-maximal effective concentration for P2X7R inhibition of lidocaine is much higher than the maximal tolerable plasma concentration where adverse effects start to develop. To overcome this, we selectively inhibit the P2X7Rs of the immune cells of the lymphatic system inducing clonal expansion of Tregs in local lymph nodes. Subsequently, these Tregs migrate throughout the body exerting anti-inflammatory activities suppressing systemic and (distant) local hyperinflammation. We illustrate this with six critically ill COVID-19 patients treated with lidocaine.

## Introduction

Hyperinflammation and acute respiratory distress syndrome (ARDS) caused by coronavirus disease 2019 (COVID-19) have become the world’s number 1 challenge. The exponential pattern in the number of severe cases in the second and third waves of the SARS-CoV-2 pandemic has shown to reach nations’ maximum ICU capacities in weeks rather than months after outbreak of the disease irrespective of rigorous population-based preventive measures. In a recently published systematic review, the case fatality rates in patients in the ICU across 7 countries vary between 14.9 and 66.7%, while the case fatality rates among those who required mechanical ventilation vary between 16.7 and 97.0% [1]. In addition, the case fatality rate in a cohort of 1035 critically ill COVID-19 patients requiring extracorporeal membrane oxygenation (ECMO, artificial lungs) is alarmingly high (37.4%) [2].

The clinical manifestations of severe COVID-19 consist of pneumonia with dyspnoea and hyperinflammation. Hyperinflammation is thought to be the basis of the development of severe and critical COVID-19 [3–5]. Currently, a clear-cut definition of hyperinflammation is lacking. Some authors describe the condition of hyperinflammation as a form of very severe inflammation with cytokine storm [6]. The criteria of hyperinflammation are not consistent and include clinical data and/or different combinations of the parameters of the activation of the pro-inflammatory response of the immune system (i.e. fever, rapid respiratory deterioration, cytokine, ferritin and/or CRP concentrations, changes in blood levels of several types of immune cells, etc., examples are presented in Table 1) [3, 4, 6–8, 9, 10–14]. In addition, the current definitions of hyperinflammatory syndrome do not provide an explanation for the frequently observed co-infections or secondary infections in COVID-19 [15, 16].

**Table 1:** Examples of the criteria of hyperinflammation. These criteria are not consistent and include different combinations of symptoms and laboratory parameters of the activation of the pro-inflammatory response of the immune system

Author	Year of publication	Criteria of hyperinflammation		Reference number
		Clinical	Laboratory or pathogenesis	
Webb BJ et al.	2020	Fever (temperature of more than 38.0°C)	Macrophage activation (ferritin concentration of 700 μg/l or more) Haematological dysfunction (neutrophil to lymphocyte ratio of 10 or more or both haemoglobin concentration of 9.2 g/dl or less and platelet count of 110 × 10^9^cells/L or less) Haematological dysfunction (neutrophil to lymphocyte ratio of 10 or more or both haemoglobin concentration of 9.2 g/dl or less and platelet count of 110 × 10^9^cells/L or less) Coagulopathy (D-dimer concentration of 1.5 μg/ml or more) Hepatic injury (lactate dehydrogenase concentration of 400 U/L or more, or an aspartate aminotransferase concentration of 100 U/L or more) Cytokinaemia (defined as an IL-6 concentration of 15 pg/ml or more, or a triglyceride concentration of 150 mg/dl or more, or a CRP concentration of 15 mg/dl or more)	[7]
Fajgenbaum DC and June CH	2020		Very severe inflammation with cytokine storm	[6]
Manson JJ et al.	2020		C-reactive protein (CRP) concentration greater than 150 mg/L Doubling of CRP concentration within 24 h from a concentration of greater than 50 mg/L Ferritin concentration of greater than 1500 μg/L	[3]
Gustine JN and Jones D	2021		Cytokine storm, dysregulated macrophage activation, impaired natural killer cell response, lymphopenia, elevated absolute neutrophil count and neutrophil/lymphocyte ratio and increased levels of neutrophil extracellular traps (NETs)	[4]
Anka AU et al.	2021		Excessive secretion of pro-inflammatory cytokines and the recruitment of pro-inflammatory cells such as granulocytes and macrophages caused by tissue injury result in a snowballing of cytokine secretion leading to a systemic inflammatory response such as macrophage activation syndrome (MAS), secondary haemophagocytic lymphohistiocytosis (sHLH—cytokine storm)	[11]
Cardone MC et al.	2020		Increased plasma levels of pro- and anti-inflammatory cytokines (IL-1β, IL-6, IL-7, IL-8, IL-9, IL-10, IFN-γ, TNF), chemokines (MCP1, MIP1A, MIP1B) and growth factors (G-CSF, GM-CSF)	[8]
Mehta P et al.	2020		Trends in laboratory results such as increasing ferritin, decreasing platelet counts or high erythrocyte sedimentation rate	12
Freeman TL et al. (2020)	2020		Vigorous stimulation of the innate immune response activating the Nod-like receptor family, pyrin domain-containing 3 (NLRP3) inflammasome pathway. This causes the release of the pro-inflammatory cytokines IL-6 and IL-1β	[13]
De Luca G et al.	2020		Elevation of CRP to ≥100 mg/L or ferritin to ≥900 μg/L in the presence of any increase in lactate dehydrogenase (LDH)	[ 14]
Bozzi G et al.	2021		Ferritin plasma levels of ≥1000 ng/mL and/or CRP of >10 mg/dl	[9]
Landewé RBM et al.	2021	Rapid respiratory deterioration on or during admission	Plus fulfilment of at least two out of three biomarker criteria: CRP of >100mg/L, serum ferritin of >900 μg/L, D-dimer of >1500 μg/L	[10]

The results of non-randomised cohort studies with controls and of retrospective observational studies suggest that IL-1 receptor blockade (anakinra) [9, 17], monoclonal antibodies against IL-6 receptors [18–21] and the combination of both drugs [22, 23] may improve survival rate in at least a subgroup of patients with COVID-19. However, in prospective randomised controlled trials with the exception of one trial with tocilizumab and sarilumab in critically patients [24], anti-inflammatory therapy with anakinra [25] or tocilizumab [26–32] did not improve the outcome in moderate, severe and critically ill COVID-19. On December 10, 2020, an editorial commented that it is disappointing that nearly 10 months into the COVID-19 pandemic, a breakthrough treatment has not been identified [33]. Researchers of the US National Institute of Allergy and Infectious Diseases stated that although Remdesivir is effective to reduce time to recovery in hospitalised COVID-19 patients [34] and dexamethasone reduces mortality in critically ill COVID-19 patients [35], there is no treatment for early or mild infection [36]. Moreover, dexamethasone raises concerns because it increased the 28-day mortality in patients who did not receive respiratory support [35] and it dampens the “alarm phase” of the inflammation process including the capacity of detecting pathogens in mammals by the immune system [37]. In addition, administration of methylprednisolone (1 mg/kg/day intravenously) in COVID-19 reduced the blood levels of NK cells, CD4^+^ and CD8^+^T-cells and increases the duration of throat viral RNA detectability indicating immune cell dysfunction [38]. Furthermore, targeted anti-viral medication has failed to treat COVID-19 effectively [39]. According to the World Health Organisation, after a record-breaking development, vaccine deployment is slow and has many challenges to overcome [40]. Vaccine hesitancy is relatively high [41] even among health care workers [42, 43]. It could take more than a year to vaccinate enough people required to make an impact on SARS-CoV-2 spreading, while therapeutic measures that can immediately attenuate the course of SARS-CoV-2-related lung damage are promptly needed on a global scale. To make the matters worse, many scientists expect that SARS-CoV-2 may become endemic and is here to stay [44].

**Fig. 1:**
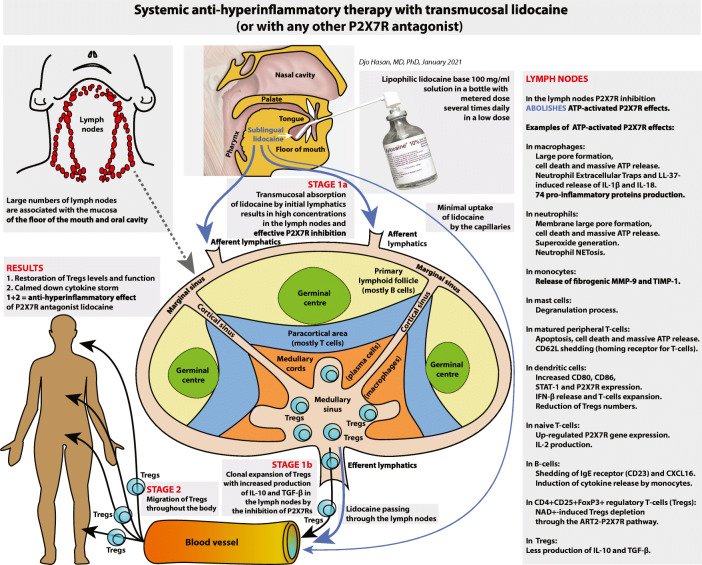
A graphical summary of the future development of the administration of lipophilic lidocaine base in the sublingual region or elsewhere in the oral cavity. We postulate that selective inhibition of the P2X7Rs of the immune cells of the lymphatic system by lidocaine suppresses hyperinflammation in two stages. Stage 1: The selective inhibition of the P2X7Rs of the immune cells residing in the lymph nodes (stage 1a) induces clonal expansion of Tregs with improved function in these lymph nodes (stage 1b); Stage 2: Subsequently, these Tregs migrate throughout the body exerting anti-inflammatory activities reducing systemic and (distant) local hyperinflammation. See text under the heading “Future development” for explanation

In this report, we developed a novel definition of hyperinflammation based on purinergic signalling. Subsequently, we describe our discovery of an old drug capable of attenuating hyperinflammation and illustrate this with six critically ill patients suffering from COVID-19. Finally, we present the future development of a new and more accessible administration route for this drug as shown in Fig. 1.

## Purinergic signalling

In 1929 adenylic acid (identical to adenosine) was identified [45], and in the same year, the adenosine triphosphate (ATP) molecule was discovered and isolated [46]. Ten years later (1939), researchers contributed to the understanding of intracellular ATP as an intracellular energy transport molecule [47–50]. In 1948 and in 1959, it was reported that extracellular ATP has a different function than ATP within the cytoplasm [51, 52]. The authors showed that extracellular ATP molecules have an intercellular signalling function. The intercellular signalling by nucleotides (ATP, ADP, UTP and UDP) and nucleoside (adenosine) is referred to as purinergic signalling. The purinergic co-transmission in neurons was discovered by Geoffrey Burnstock in 1972 [53]. It took over 20 years for the importance of purinergic signalling to be accepted [54, 55]. Finally, researchers of the University of Ferrara first reported that the P2Z receptor (the former name of the P2X7R) plays an intriguing role in immunity, inflammation and cell death [56].

The intracellular levels of ATP are high at millimolar concentrations (2–8 mM) [57], and the ATP concentrations in synaptic vesicles are even higher in the range of 5 to 100 mM [58]. In contrast, under normal resting conditions, the extracellular levels of ATP are quite low at nanomolar concentrations (<3 nM) [57, 59]. Under specific conditions, ATP release can rise by more than 1000-fold [53, 57, 60, 61] and leads to a significant increase in the extracellular levels of ATP. The resulting significant increase in extracellular nucleotides and adenosine concentrations activates their purinergic receptors inducing certain cellular functions. Examples of such conditions are membrane depolarisation (i.e. sympathetic neuron endings) [53], mechanical stress (i.e. high mechanical power ventilation) [59–63], hypoxia [64], hyperosmosis, hypotonic and isotonic stress of endothelial cells [65–68], inflammation [69, 70], surfactant release by alveolar epithelial type II cells [59–61], mucine release by airway smooth muscle cells [71], insulin release by pancreatic islet beta-cells [72, 73], etc. There is an exception to this concept: Although a spontaneous ATP-induced inward Ca^2+^ current through the P2X7R could not be detected below extracellular ATP levels of 200 μmol/ml [74], low tonic basal activation of P2X7R at nanomolar extracellular ATP concentrations promotes serum independent cellular proliferation [75], promotes closure of the wound area in scratch wound assay [76], protects from apoptosis [77], initiates anaerobic glycolysis independent of the oxygen contents [78], etc. (Table 2, rows 80*–*85). However, low tonic basal activation of the P2X7Rs by extracellular ATP does not cause a pro-inflammatory response of the immune system. Therefore, this topic is beyond the scope of this paper and will not be discussed here.

**Table 2:** Summary of the effects of extracellular nucleotides and nucleoside on the innate and adaptive immune system through different purinergic receptors. *AdoR a*denosine receptor; *TNF-α t*umour necrosis factor alpha; *FcγR r*eceptors belonging to the immunoglobulin superfamily; *IFN-γ i*nterferon gamma; *IFN-β i*nterferon beta; *MAC-1 m*acrophage-1 antigen comprised CD11b (integrin αM) and CD18 (integrin β2); *CpG-A* oligodeoxynucleotides; *PARP* Poly ADP ribose polymerase; *FMLP N*-Formylmethionyl-leucyl-phenylalanine, a chemotactic factor; *COX-2 c*ytochrome C ox*i*dase polypeptide II; *PGE2 p*rostaglandin E2; *MIP-1α m*acrophage inflammatory protein 1 alpha (MIP-1α = *CCL3* chemokine ligand 3 ), MIP-1β (CCL4), MIP-2α (CXCL2 chemokine CXC motif ligand 2) and MIP-3α(CCL20);*RANTES* (Regulated on Activation, Normal T cell Expressed and Secreted, CCL5); *LTB4* Leukotriene B4; *LTA4* Leukotriene A4; *VCAM-1 v*ascular cell adhesion molecule 1 (CD106);*ICAM-1 i*ntercellular adhesion molecule 1 (CD54);*HMGB-1 h*igh-mobility group box 1 (belongs to danger*-*associated molecular patterns); *MCP-1 m*onocyte chemoattractant protein 1 (CCL2);*FoxP3* Forkhead box P3; *CTL c*ytotoxic T lymphocyte; *Th* T helper cell; *CTLA-4 cytotoxic T-lymphocyte-associated protein 4 (CD152); CD39 n*ucleoside triphosphate diphosphohydrolase 1 (NTPD1);*CD73*5'-nucleotidase (5'-NT); *VEGF v*ascular endothelial growth factor; *IDO*Indoleamine-pyrrole 2,3-dioxygenase; *α-SMA* alpha smooth muscle actin; *CTGF* connective tissue growth factor (CCN2);*bFGF b*asic fibroblast growth factor; *TCR*T-cell receptor; *NFAT n*uclear factor of activated T *cells; NLRP3*Nod-like receptor family pyrin domain containing 3 gene; *ART2-P2X7 pathway* extracellular NAD+-induced ATP-independent p2X7R activation involving ADP-ribosyltransferase 2; *MMP-9 m*atrix metalloproteinase-9;*TIMP-1* tissue inhibitor of metalloproteinase 1; *LC-MS/MS l*iquid chromatography and tandem mass spectrometry; *STAT-1 s*ignal transducer and activator of transcription 1. Updated table, source: Hasan D, et al. (2017) [60] with permission

Effects of extracellular nucleotides and nucleoside on the innate and adaptive immune system through different purinergic receptors					
Row number	Receptor	Ligand [52]	Immune cell expression or experimental model	Results of receptor signalling	Reference number
1	AdoRA1	Adenosine	Neutrophils	Promotes chemotaxis	[79, 80]
2			Neutrophils	Increases adherence to endothelial cells	[81]
3			Neutrophils	Inhibits TNF-α release	[82]
4			Neutrophils	At low concentrations adenosine enhances FcγR phagocytosis and actin dynamics	[83–85]
5			Neutrophils	Restores LPS-inhibited chemotaxis	[86]
6			Resting DCs (rDCs)	Inhibits vesicular MHC class I cross-presentation	[87]
7			Plasmacytoid DCs (pDCs)	Potent chemoattractants, reduces IL-6, IL-12 and IFN-γ release	[88]
8	AdoRA1 and AdoRA2A		CD39^high^B-cells (Bregs)	Promotes expansion and function of CD39^high^ B-cells	[89, 90]
9	AdorA2A	Adenosine	Monocytes	Inhibits IL-12 and TNF-α release	[91, 92]
10			Neutrophils	Promotes chemotaxis	[80]
11			Neutrophils	Inhibits oxygen radical generation	[79]
12			Neutrophils	Inhibits upregulation of beta2 integrins or MAC-1 (CD11/CD18) and shedding of L-selectin by FMLP	[93, 94]
13			Neutrophils	Promotes Cox-2 and PGE2 release	[95]
14			Neutrophils	Decreases adherence to endothelial cells	[81]
15			Neutrophils	Decreases adherence to fibrinogen coated surfaces	[96]
16			Neutrophils	Inhibits TNF-α release and chemokines MIP-1α (CCL3), MIP-1β (CCL4), MIP-2α(CXCL2) and MIP-3α (CCL20)	[82, 97]
17			Neutrophils	At high concentrations adenosine inhibits FcγR functions and actin dynamics	[83–85]
18			Neutrophils	Inhibits leukotriene (LTB4, LTA4) synthesis	[98–102]
19			Neutrophils	Inhibits degranulation and superoxide release or oxidative burst	[96, 103–106]
20			Neutrophils	Delays neutrophil apoptosis	[107]
21			Neutrophils	Inhibits autophagy suppressed apoptosis of neutrophils by blocking caspase-8, caspase-3 and PARP signalling	[108]
22			Mast cells	Increases IL-1β, IL-3 and IL-8 release	[109]
23			Macrophages	Inhibits LPS-induced TNF-α release	[110]
24			Endothelial cells	Reduces thrombin-induced permeability. Inhibits thrombin-mediated expression of VCAM-1, ICAM-1 an E-selectin. Inhibits thrombin induced increase of IL-6, HMGB-1; chemokines, MCP-1 (CCL-2), CXCL-1 and CXCL-3	[111]
25			Naïve T-cells	Promotes the differentiation towards CD4^+^FoxP3^+^Lag3^+^ Tregs, inhibits Th1 and Th17 differentiation, inhibits IL-6 secretion and increases TGF-β secretion	[112]
26			Th1, Th2 and Th17cells	Reduces release of IL-2, IL-4, TNF-α and IFN-γ	[113–115]
27			CD8^+^CTLs, Th1, Th2	Reduces release of IL-2, TNF-α, IFN-γ. Inhibits CD8^+^CTL and Th1 expansion to alloantigens	[116]
28			CD4^+^ T-cells	Inhibits TCR-mediated IFN-γ release	[117]
29			CD4^+^CD25^+^FoxP3^+^ Tregs	Increases number of Tregs and increases the expression of CTLA-4 receptor	[118]
30			CD4^+^CD25^+^FoxP3^+^ Tregs	Upregulates ecto-enzymes CD39 and CD73 expression accelerating adenosine generation from extracellular ATP	[118]
31			AdoRA2A-knockout mice	Bleomycin-induced fibrosis is more severe and elevated TGF-β is higher than in wild-type mice	[119]
32			Human leukaemia monocytic cell line THP-1 cells	TNF-α upregulates the expression of AdoRA2A followed by the increase of the expression of CD163 and TGF-β1	[120]
33			Human CD4+ CD25+ CD127low/− Tregs and CD8+ T-cells	Tregs from gastric cancer patients hydrolyse ATP into adenosine. Adenosine synthesised by Tregs promotes apoptosis and suppresses proliferation of CD8+ T-cells. Tregs reduces CD8+ T-cell activity by promoting cAMP synthesis. Tregs Inhibit the immune function of CD8+ T-cells through A2aR pathway	[121]
34	AdoRA2A and AdorA2B	Adenosine	Macrophages	Differentiation of monocytes towards M2 macrophages with VEGF and IL-10 release	[122–126]
35			Macrophages	Inhibits LPS-induced IL-6, MIP-2 and TNF-α release	[127, 128]
36	AdoRA2B	Adenosine	Neutrophils	Inhibits neutrophil recruitment and transmigration, release of TNF-α, IL-6, MIF-1α and IL-8	[129, 130]
37			Neutrophils	Inhibits superoxide generation	[131]
38			Neutrophils	Inhibits TNF-α release	[82]
39			Mast cells	Stimulates degranulation (mice), IL-13, IL-4 (Th2 cytokines)	[109]
40			Macrophages	Stimulates IL-10 release	[132]
41			DCs	Differentiation and maturation towards regulatory DCs: High level expression of angiogenic (VEGF), wound healing (IL-6), chemokine (IL-8), immune suppressing (IL-10) and tolerogenic (IDO) factors	[133]
42			DCs	Promotes Th17 differentiation via stimulation of IL-6 release	[134]
43			Bone marrow cells	Promotes differentiation towards CD11c^+^Gr-1^+^ DCs that promotes Th17 response	[135]
44			Myeloid cells in systemic bleomycin-induced pulmonary fibrosis	Myeloid cells AdorA2B knock out mice show a reduction in CD206 and arginase-1 (markers for M2 macrophages). 10-fold reduction in IL-6 and 5-fold reduction in hyaluronan (both linked to pulmonary fibrosis)	[136]
45			Mast cells	Upregulates the IL-4 and IL-13 release	[109]
46			B-cells	Induces Ig-E release through IL-4 and IL-13 release by the adenosine-activated mast cells	[109]
47			Endothelial cells	Reduces endothelial permeability, ICAM-1, P-selectin and E-selectin (adhesion molecules)	[137]
48			Endothelial cells	Stimulates basic fibroblast growth factor (bFGF) and insulin-like factor-1 release	[138]
49			Bronchial epithelial cells	Increases IL-19 release	[139]
50			Human leukaemia monocytic cell line THP-1 cells	Increases TNF-α release through mast cell-released IL-19	[139]
51			Renal fibroblasts	Increases the expression of α-SMA, IL-6, TGF-β, CTGF and fibronectin (pro-fibrotic mediators)	[140]
52			AdoR2B knock-out mice	Negligible effect on bleomycin-induced acute lung injury. Enhanced loss of barrier function	[141]
53			AdorR2B knock-out mice exposed to systemic bleomycin	Substantial reduction of fibrosis and IL-6 production	[141]
54			Specific pathogen-free male Sprague-Dawley rats	Inhibition of AdoRA2B: Attenuates necrotizing enterocolitis in newborn rats and protects against body weight loss, decreases myeloperoxidase activity, decreases TNF-α, IFN-γ and IL-6 intestinal levels and increases IL-10 intestinal levels	[142]
55			RAW 264.7 murine macrophage cells with and without transfection with AdoRA2B siRNA cultured with B. abortus 544 biovar 1 strain (ATCC 23448)	Blocking of Adora2b using siRNA induces productions of IL-6, MCP-1 and TNF-α in cells without infection. Adora2b siRNA macrophages have reduced uptake of *B. abortus*. Inhibition of AdoRA2B results in higher total weight of the spleens and less Brucella colonisation in this organ, decreases IL-10, elevates the levels of IFN-γ and IL-12 at three days p.i. and elevates the levels of IL-6, TNF-α and IL-12 at 14 days p.i.	[143]
56	AdoRA2B and AdoRA3	Adenosine	Mast cells	Stimulates IL-8(chemokine) and VEGF (angiogenic) release	[144]
57			Peritoneal macrophages from wild type, AdoRA2A knockout and AdoRA3 knockout FVB or C57BL/6 male mice	Simultaneous adenosine AdoRA2B and AdoR3 signalling is required to promote chemotactic migration of macrophages towards the apoptotic cells	[145]
58	AdoRA3	Adenosine	Neutrophils	Synergistic AdorA3 and P2Y2R neutrophil chemotaxis through autocrine ATP release by pannexin-1, extracellular conversion of ATP to adenosine by the ecto-enzymes (CD39 and CD73), strategic translocation of the FPR, AdorA3, P2Y2, pannexin-1 receptors and CD39, Cd73 to the leading edge of the neutrophils. This results in the amplification of the chemoattractant gradient sensing and the self-generated gradients	[146–152]
59			Microglial cells and colonic epithelial cells	Suppresses LPS-induced TNF-α production	[153, 154]
60			Anti-CD3-activated CD8^+^ CTLs	Reduces the expression of mRNAs coding for granzyme B, perforin, Fas ligand and TNF-related apoptosis-inducing ligand (TRAIL). Diminishes Nalpha-CBZ-L-lysine thiobenzylester esterase activity (enzyme with cytotoxic activity). Reduces IL-2 sand IFN-γ release.	[155]
61			Microglia BV-2 cell line	Reduces elevated hydrostatic pressure-induced inducible nitric oxide synthase (iNOS) expression, microglia migration and phagocytosis in BV-2 cells	[156]
62			AdoRA3 knock-out mice exposed to intratracheal bleomycin	Increase in eosinophil numbers and selective upregulation of eosinophil-related chemokines and cytokines. But decreased eosinophil peroxidase activity in the BALF	[157]
63			Human colonic mucosa biopsies	Significantly decreases TNF-α and IL-1β production and attenuates the NF-κBp65 activation	[158]
64	P2X1R	ATP	Neutrophils and platelets	Promotes thrombosis and fibrinogenesis: Keeps circulating neutrophils in quiescent state, recruit neutrophil to the injury site, activate adhered neutrophils and platelets	[159]
65			Bovine polymorphonuclear leukocytes (PMNs)	Oleic acid (OA) and linoleic acid (LA) induce Neutrophil Extracellular Traps (NETs) formation and ATP release via PANX1 and activation of P2X1	[160]
66	P2X1R, P2X4R and P2X7	ATP	Naïve T-cells	TCR stimulation results in the translocation of pannexin-1 hemichannels, P2X1Rs and P2X4Rs to the immune synapse. While the P2X7Rs remain uniformly distributed. This process is required to induce calcium entry, NFAT and release of IL-2	[161]
67	P2X3R	ATP	Mast cells	Increases the expression of IL-4, IL-6, IFN-γ, TNF-α, RANTES and M IP-2. Increases the release of IL-6 and IL-13	Article retracted due to figure irregularities [162]
68	P2X4R	ATP	γδ T-cells	Activates and upregulates TNF-α and IFN-γ release	[163]
69			Microglial cells	Promotes survival after LPS-activation	[164]
70			CD4^+^T-cells from Human peripheral blood mononuclear cells (PMBCs)	Chemokine stromal-derived factor-1α (SDF-1α) triggered mitochondrial ATP production, rapid bursts of ATP release and increased migration of primary human CD4+ T cells. This process depended on pannexin-1 ATP release channels and autocrine stimulation of P2X4Rs. SDF-1α stimulation caused localised accumulation of mitochondria with P2X4Rs near the front of cells, resulting in a feed-forward signalling mechanism that promotes cellular Ca2+ influx and sustains mitochondrial ATP synthesis at levels needed for pseudopod protrusion, T cell polarisation and cell migration	[165]
71			Chinese hamster ovary (CHO) cells transfected with human Kv1.3 cDNA and rat P2X4 construct	The voltage-gated potassium channel Kv1.3 is required for microglia activation . Inhibition of Kv1.3 channels completely nullified the ability of Kv1.3 to normalise membrane potential changes, resulting in excessive depolarisation and reduced calcium transients through P2X4 receptors	[166]
72	P2X4R and P2Y11R	ATP	CD4^+^T-cells from Human peripheral blood mononuclear cells (PMBCs), Jurkat T cells (clone E6-1) and U-937 cells	P2Y11Rs retract from the immune synapse (IS) towards the back of cells where their stimulation by extracellular ATP induces cAMP/PKA signalling that redirects mitochondrial trafficking to the IS. P2Y11Rs thus reinforce IS signalling by promoting the aggregation of mitochondria with panx1 ATP release channels and P2X4 receptors at the IS. This dual purinergic signalling mechanism involving P2X4Rs and P2Y11Rs focuses mitochondrial metabolism to the IS where localised ATP production sustains synaptic activity in order to allow successful completion of T cell activation responses	[167]
73			CD4+ T-cells from Human peripheral blood mononuclear cells (PMBCs)	Autocrine P2X4R and simultaneous P2Y11R activation regulate mitochondrial metabolism, T-cell polarisation, pseudopod formation and redistribution of P2Y11Rs to the back of polarised T-cells resulting in T-cell trafficking. Exogenous activation of P2Y11R blocks T-cell trafficking	[168]
74	P2X4R and/or P2X7R	ATP	Neutrophils, monocytes, macrophages, DCs, CD4^+^ T-cells, CD8^+^ T-cells, iNKTs, adenoviral infected macrophages and alveolar epithelial cells	Mediates NLRP3 inflammasome-dependent IL-1β and IL-18 secretion (signal 2, non-classical pathway), increases IL-6 production	[169–176]
75	P2X5R		C57BL/6J mice: wild type, P2X5R knockout, P2X7R knockout and P2X5R/P2X7R knockout and their bone marrow-derived macrophages (BMMs)	P2X5R-deficient BMMs exhibit defective cytosolic killing of L. monocytogenes P2X5R is required for L. monocytogenes-induced inflammasome activation and IL-1β production and that defective L. monocytogenes killing in P2X5R-deficient BMMs is substantially rescued by exogenous IL-1β or IL-18. The P2X5-dependent anti-L. monocytogenes immunity is independent of the ATP-P2X7 inflammasome activation pathway	[177]
76	P2X7R	Unactivated state in the absence of ATP	Macrophages and P2X7R-transfected HEK-293 cells	P2X7 is a scavenger receptor for apoptotic cells in the absence of i ts ligand ATP	[178, 179]
77		ATP release channel	Alveolar epithelial type I cells (AT I cells), mice osteoclast cells, murine neuroblastoma cells, astrocytic cell line, mice astrocytes, B16 melanoma cells	Release ATP after mechanical deformation (AT I cells), spontaneously (osteoblast cells), after activation (neuroblastoma cells, astrocytic cell line), after γ irradiation (melanoma cells)	[180–185]
78		P2X7R-mediated ATP release	Mouse 3T3fibroblasts	P2X7R-mediated ATP secretion is accompanied by depletion of cytosolic ATP	[186]
79			Bone marrow–derived dendritic cells from WT mice and Panx1−/− C57BL/6 mice	Upon stimulation of the P2X7 receptor by ATP, Panx1 contributed to fast DC motility by increasing the permeability of the plasma membrane, which resulted in supplementary ATP release	[187]
80		ATP, low tonic basal activation	HET293 and HELA cells	Elevates mitochondrial calcium and potential, cellular ATP levels and promotes serum-independent growth. This process requires a full pore-forming function	[75]
81			In-vitro scratch wound assay with HaCat cells (human skin keratinocytes)	Medium hyaluronan fragment (MMW-HA, between 100 and 300 kD) increases tight junction ZO-1 protein expression and induces a low activation of P2X7 receptor resulting in improved closure of the wound area. This is accompanied by pore formation as shown by Yo-Pro-1 cellular uptake. The P2X7R antagonist brilliant blue G (BBG) completely inhibits this process	[76]
82			HEK293 and NIH3T3 cells	Increases the Ca2+ content of the endoplasmic reticulum, activates NFATc1 and protects from apoptosis	[77]
83			PC3 cells LNCaP, Kelly, RPMI-8226, DU145 and SK-MEL-5 cells	Drives the expression of nfP2X7, a key mediator of cell survival	[188]
84			Osteoclast-like cells	Promotes the increase in the extracellular adenosine concentrations	[189]
85			HEK293 cells	The initiation of anaerobic glycolysis independent of the oxygen content: Upregulates glucose transporter Glut1 (thus enhances intracellular glycogen stores); Upregulates glycolytic enzymes (PFK, G3PDH, PKM2), phosphorylated Akt/PKB and hypoxia-inducible factor 1a (HIF-1a) expression Impedes the Krebs cycle independent of the oxygen concentrations: Promotes pyruvate dehydrogenase kinase 1 (PDHK1) and inhibits of pyruvate dehydrogenase (PDH, conversion of pyruvate to acetyl-coA)	[78]
86		ATP >1 mM, vigorous activation	C57BL/6 mice	P2X7 activation inhibits the suppressive potential and stability of Tregs. In contrast, P2X7R inhibition promotes the conversion of the cell-autonomous conversion CD4+ T cells into Tregs after stimulation of their T-cell receptors (TCRs)	[190]
87			C57BL/6 wild type and P2X7 knockout mice	P2X7 knock-out mice show an increase of CD90/CD45RB^low^ FoxP3^+^ Tregs in colon lamina propria, prevents Tregs death in mesenteric lymph nodes and these Tregs produce more IL-10. Colitis is prevented or reduced and P2X7 knock-out mice. Treg cells lacking the P2X7 receptor have higher levels of integrin CD103	[191]
88			C57BL/6 mice	P2X7R activation reduces the frequency of Tregs and P2X7R inhibition increases the expansion of Tregs	[192]
89			C57BL/6 wild type, P2X7 knockout mice and foetal thymus organ culture	Selectively increases immature γδ+CD25+ cells which are much more competent to release ATP than pre-TCR expressing cells following TCR stimulation and Ca2+ influx. Genetic ablation as well as pharmacological antagonism of P2X7 results in impaired ERK phosphorylation, reduction of early growth response (Egr) transcripts induction, diversion of γδTCR-expressing thymocytes towards the αβ lineage fate and increased representation of the Id3-independent NK1.1-expressing γδ T-cell subset in the periphery	[193]
90			C57BL/6J mice implanted with melanoma B16F10 cells	P2X7 activation in tumour infiltrating CD8+ lymphocytes (TILs) promotes cell cycle arrest and p38 MAPK mediated cellular senescence in the tumour microenvironment	[194]
91			BAC1.2F5 macrophage cell line	P2X7 receptor-dependent blebbing and the activation of Rho-effector kinases, caspases and IL-1μβ release	[195]
92			Dendritic cells cultured from mice bone marrow precursor cells	Autocrine-mediated (pannexin-1 channels) fast migration of dendritic cells through the reorganisation of the actin cytoskeleton	[187]
93			RAW 264.7 murine macrophages	mediates actin reorganisation and membrane blebbing via p38 MAP kinase and Rho	[196]
94			Monocytes	Induces MMP-9 and TIMP-1 release, fibrosis markers	[197]
95			M1 macrophages	Induces release of 74 pro-inflammatory proteins detected by antibody protein array and 33 inflammatory proteins detected by LC-MS/MS	[198]
96			M2 macrophages	Induces release of 21 anti-inflammatory proteins detected by LC-MS/MS	[198]
97			Macrophages	Enhances intracellular bacterial killing	[199]
98			Macrophages and P2X7R-transfected HEK-293 cells	Mediates rapid uptake of beads and bacteria in the absence of serum after ATP activation	
99			Mast cells	Induces degranulation	[200]
100			Naïve NKTs	Facilitates NAD^+^-induced inhibitory signal through the ART2-P2X7 pathway resulting in non-functional NKTs	[201]
101			Activated NKTs	Facilitates NAD^+^-induced stimulatory signal through the ART2-P2X7 pathway resulting in functional NKTs with increased IFN-γ and IL-4 release	[201]
102			B cells	Induces shedding of IgE receptor (CD23) and CXCL16. Soluble CD23 sustains growth of B-cell precursors, promotes B and T cell differentiation and drives cytokine release from monocytes. CXCL16 is a chemoattractant for lymphocytes	[202, 203]
103			CD11c^+^CD103^+^ DCs	Mediates infection-induced rapid recruitment of CD11c^+^CD103^+^ DC subsets into the epithelial layer of the gut	[204]
104			Naïve T-cells	TCR stimulation triggers rapid release of ATP and upregulates P2X7 gene expression. Autocrine ATP stimulation through the P2X7R is required to for the TCR-mediated calcium influx, NFAT activation and IL-2 production	[205]
105			T follicular B helper cells (Tfh cells)	Reduces and thus controls the number of Tfh cells in Peyer’s patches in the gut with high-affinity IgA responses to promote host-microbiota mutualism	[206]
106			CD4^+^CD25^+^FoxP3^+^ regulatory T-cells (Tregs)	Facilitates NAD^+^-induced Tregs depletion through the ART2-P2X7 pathway	[207]
107			DCs	Increases CD80, CD 86, STAT-1 and P2X7R expression, IFN-β release and T-cells expansion. Reduces Tregs numbers	[208]
108			AT I cells	Induces VCAM-1 shedding and neutrophil transmigration in acute lung injury	[209]
109			Human endometrial mesenchymal stem cells, murine luteal cells	Causes cell cycle arrest in G0/G1 phase and suppresses cell replication	[210, 211]
110			Brain-derived type-2 astrocyte cell, mesangial cells	Stimulates TGF-β mRNA expression	[212, 213]
111					
112			Sprague-Dawley rats with and without spinal cord injury	After spinal cord injury P2X7R of microglia was upregulated by BzATP and down-regulated by P2X7R antagonist A-438079. Upregulation of P2X7R on microglia coincides with increase of neuroinflammation after spinal cord injury. P2X7R of microglia participates in spinal cord-mediated neuroinflammation via regulating NLRP3 inflammasome-dependent inflammation	[214]
113			Abdominal cells of male Kunming mice of clean grade	Transfection of the long non-coding siRNA uc.48+ decreases the upregulated mRNA and protein levels of the P2X7 receptor in diabetes mellitus type 2 mice model	[215]
114			Human embryonic kidney cells (HEK293T)	Promotes paxillin and NLRP3 migration from the cytosol to the plasma membrane and facilitates P2X7R-paxillin interaction and PaxillinNLRP3 association, resulting in the formation of the P2X7R-Paxillin-NLRP3 complex. Paxillin is essential for ATP-induced NLRP3 inflammasome activation in mouse bone marrow-derived macrophages and bone marrow-derived dendritic cells (PMDCs) as well as in human PBMCs and THP-1-differentiated macrophages	[216]
115			P2Y1R knockout, P2Y12R knockout, P2Y13R knockout, P2X7R knockout, NLRP3 knockout and wild type C57BL/6 mice	Aggravates inflammatory bowel disease through ERK5-mediated tyrosine phosphorylation of the adaptor protein ASC essential for NLRP3 inflammasome activation and the secretion of IL-1β	[217]
116			C57BL/6 mice: Wild-type, P2X7 knockout, NLRP3 knockout and caspase-1/11 knockout	Induces the release of extracellular vesicles containing CD14. Extracellular CD14 induced during sepsis controls bacterial dissemination and cytokine secretion	[218]
117			C57BL/6 J mice and their peritoneal macrophages, immortalised human liver stellate cell line LX-2 and immortalised human leukaemia monocytic cell line THP-1 cells	Blockade of P2X7R reverses TAA-induced liver fibrosis thioacetamide and attenuates thioacetamide-induced inflammatory response by inhibiting NLRP3 and NF-κB activation in mice liver. P2X7R overexpression significantly enhances TGF-β-increasedα-SMA and collagen I protein and mRNA level in LX-2 cells. Macrophages increase fibrogenesis in LX-2 HSCs through the release of IL-1β by P2x7R stimulation	[219]
118			Macrophages derived from human leukaemia monocytic cell line THP-1 cells cultured with T. pallidum with and without P2X7R gene siRNA-transfection	T. pallidum increases both the mRNA and protein levels of P2X7R, increases levels of NLRP3 mRNA expression and IL-1β. SiRNA transfection of the macrophages reduces the percentage of spirochete-positive macrophages and spirochete internalisation	[220]
119			Human and mice macrophages	Enhances the Neutrophil Extracellular Traps (NETs) and LL-37 formation (an antibacterial protein externalised on NETs) activated caspase-1, the central enzyme of the inflammasome, in both human and murine macrophages, resulting in release of active IL-1β and IL-18. LL-37 activation of the NLRP3 Inflammasome utilises P2X7R-mediated potassium efflux. IL-18 can stimulate NETosis (NET activation and release) in human neutrophils	[221]
120		ATP >1 mM, prolonged vigorous activation	Macrophage, HeLa cells, 1321-N1 astrocytes and HEK293 cells	Induces pannexin-1 mediates large pore formation and IL-1β release	[222]
121			Human neutrophils and HL-60 cells	Mediates membrane large pore formation and superoxide generation	[223]
122			Matured peripheral T-cells	High dose ATP promotes apoptosis, cell death and CD62L shedding (homing receptor for central T-cells) independent from the NAD^+^-induced ART2-P2X7 pathway	[224–226]
123			J774 cells and HEK cells expressing the P2X7 receptor	Promotes the formation of pores permeable to very large ions leading to cytolysis	[227]
124	P2X7R and P2Y13R		Human mast cell line HMC-1 and rat basophilic leukaemia cell line (RBL-2H3) with and without transfection of P2Y13-siRNAs and P2X7-siRNAs	P2Y13R mediates nanomolar mechanical-induced ATP release. P2X7R mediates micromolar mechanical-induced ATP release	[228]
125	P2Y1R and P2Y12R	ADP>ATP	Platelets	P2Y1R and P2Y12R synergistic action in thrombin-induced platelet activation	[229]
126			Platelets	C-activation of P2Y1R and P2Y12R by ADP upregulates the expression of P-selectin (CD62P)	[230]
127	P2Y2R	UTP≥ATP	Neutrophils	Synergistic AdorA3 and P2Y2R neutrophil chemotaxis (see under AdoRA3 above)	[146, 147]
128			Neutrophils and fibroblasts	Mediates recruitment of neutrophils into the lungs, proliferation and migration of lung fibroblasts and IL-6 production	[231]
129			Monocyte-derived DCs (moDCs), eosinophils	Promotes chemotaxis	[232]
130			Eosinophils	Induces VCAM-1 expression	[233]
131			Peritoneal macrophages (RPMs) isolated from resting C57/BL6 mice	P2Y2R-Induced c-Jun N-terminal kinase (JNK) activation is responsible for Increased in IL-1 β production	[234]
132			Murine model of cutaneous leishmaniasis	Induces CASP-1 activation and IL-1β secretion during *L. amazonensis* infection. IL-1β/IL-1R signalling is crucial for P2Y2R-mediated protective immune response in an experimental model of cutaneous leishmaniasis	[235]
133			ChAT^BAC^-eGFP mice	Elicits tracheal brush cells generation of cysteinyl leukotrienes. Aeroallergens elicit P2Y2-dependent tracheal brush cells cysteinyl leukotrienes generation and tracheal brush cells -dependent airway eosinophilia	[236]
134	P2Y4R and P2Y12	UTP≥ATP, ADP>ATP, respectively	Microglial cells	P2Y4R and P2Y12R synergistic action increases microglial chemotaxis	[237, 238]
135	P2Y6R	UDP>UTP>>ATP	Neutrophils	Induces neutrophil activation and extracellular trap formation	[239]
136			Human leukaemia monocytic cell line THP-1 cells	Induces IL-1β release	[240]
137			Macrophages	Induces MCP-3(CCL7) expression in response to necrotic tissue cells	[241]
138			Microglial cells	Facilitates phagocytosis	[242]
139			Microglial cells	Induces the expression of MCP-1 (CCL-2)	[243]
140			Microglial cells	Promotes phagocytosis	[244, 245]
141			Basophils	UDP promotes IgE-dependent degranulation	[246]
142			Plasmacytoid DCs	UDP and UTP strongly inhibit IFN-alpha secretion induced by influenza virus or CpG-A	[247]
143			Tissue cells	Induces IL-1α, IL-8/CXCL8 and IL-6 release	[240, 248, 249]
144			Tissue cells	Induces IFN-β release	[250]
145			Wild-type C57BL/6 mice and their DCs	Inhibits the maturation and activation of DCs via suppressing the activation of the transcription factor NF-κB. In-vitro studies show that P2Y6 signalling inhibits the production of IL-12 and IL-23 and the polarisation of Th1 and Th17 subsets mediated by DCs. Mice lacking P2Y6 develop more severe experimental autoimmune encephalomyelitis compared with wild-type mice	[251]
146			Institute of Cancer Research (ICR) mice, primary microglial cells and neurons from Sprague Dawley rat	Transient middle cerebral artery occlusion (tMCAO) increases P2Y6R expression. P2Y6 receptor-specific inhibitor blocked the phagocytosis of primary microglia under LPS and UDP stimulation. P2Y6 receptor-specific inhibitor down-regulates myosin light-chain kinase (MLCK) required for the cytoskeletal remodelling for the formation of the phagocytic cup. Inhibition of P2Y6R does not reduce the tMCAO-induced upregulation of mRNA levels of IL-1α, IL-1β, IL-6, IL-10, TNF-α and TGF-β	[252]
147	P2Y11R	ATP	Neutrophils	Inhibits neutrophil apoptosis	[253]
148			Neutrophils	Enhances chemotactic response	[254]
149			Neutrophils and moDCs	Induces maturation of the granulocytic progenitors and monocyte differentiation	[255, 256]
150			moDCs	Inhibits migratory capacity	[257]
151			moDCs	Induces IL-8 release	[258]
152			Monocytes	Autocrine differentiation towards M1 macrophages, induces IL-1β, IL-6, IL-12 and TNF-α release	[259]
153	P2Y12R	ADP>ATP	Monocytes	Increases monocyte adhesion	[260]
154			Vascular smooth muscle cells	Upregulates MCP-1 (CCL-2)	[260]
155			DCs	Increases antigen endocytosis with subsequent enhancement of specific T-cell activation	[261]
156			Microglial cells	Induces movement of juxta-vascular microglial processes to close the injured blood-brain barrier (BBB) and microglial activation	[262, 263]
157			Microglial cells	Promotes migratory, inflammatory (TNF-αand IL-6 release) responses	[264]
158			Microglial cells	ADP treated microglial cells induces CCL3 expression in activated T-cells	[265]
159			Murine model of sepsis, caecal ligation and puncture (CLP). Co-cultures of human platelets and T-cells with or without anti-CD3/CD28	Blockade of the P2Y12 signalling pathway restrains Treg proliferation in vivo and in vitro	[266]
160			Male C57BL/6 mice microglial cells	Mediates microglial activation via Ras homolog family member A/Rho-associated protein kinase (RhoA/ROCK) pathways	[267]
161	P2Y13R	ADP>ATP	Red blood cells	Inhibits ATP release	[268]
162	P2Y14R	UDP>UDP-glucose	Neutrophils	Enhances neutrophil chemotactic response through IL-8 dependent manner	[269, 270]
163			Sprague-Dawley Rats and human leukaemia monocytic cell line THP-1 cells	P2Y14R knockout reduces in-vivo and in-vitro monosodiu m urate-indu ced NLRP3 inflammasome activation, increased expressions of NLRP3, ASC, active Caspase-1 and downstream active IL-1β. Therefore, increases resistance to monosodium urate-induced acute gouty arthritis. Decreased AMP reverses the in-vivo and in-vitro protective effect of P2Y14R knockout	[271]

**Fig. 2:**
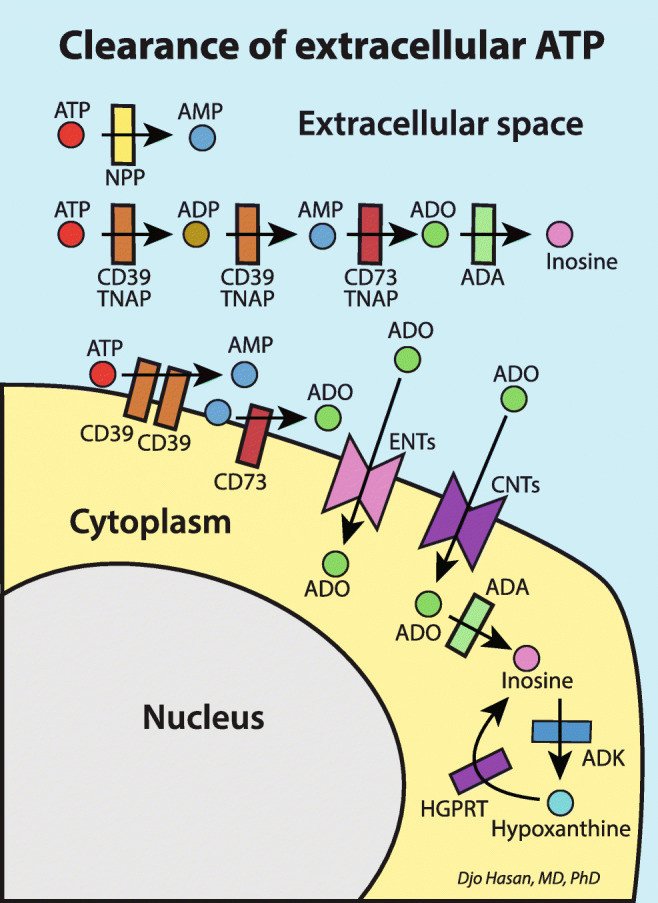
Clearance of *e*xtracellular ATP and adenosine by ectonucleotidases and soluble extracellular nucleotidases [272–274,275]. This process is indispensable to enable receptors to recover from desensitisation following receptor activation (resensitisation, see text under the heading “Purinergic signalling in inflammation and hyperinflammation” for explanation). CD39*,*Ecto-nucleoside triphosphate diphosphohydrolase 1-3 (ENTPD 1-3); CD73*,* Ecto-5′-nucleotidase (5’-NT); NPP*, n*ucleotide pyrophosphatase/phosphodiesterase; TNAP*, t*issue *n*onspecific *a*lkaline *p*hosphatase; ADA*, a*denosine deaminase; ADK*,* adenosine kinase; HGPRT*, h*ypoxanthine-guanine phosphoribosyltransferase; ATP*, a*denosine triphosphate; ADP*, a*denosine diphosphate; AMP*, a*denosine monophosphate; ADO*,* adenosine; ENTs*, e*quilibrative nucleoside transporters; CNTs*, c*oncentrative nucleoside transporters

Clearance of the ATP molecule in order to avoid accumulation in the extracellular space is performed by enzymes attached to the outside of the cell membranes (ecto-enzymes) and by soluble enzymes excreted to the extracellular space (Fig. 2) [57, 272–275]. A proportion of the enzymatic-breakdown product of ATP adenosine enters the cells via the equilibrative nucleoside transporters (ENT1 and ENT2) and concentrative nucleoside transporters (CNT1 and CNT2) (Fig. 2) [57, 60, 61]. The release and subsequently clearance of the extracellular nucleotides and adenosine cause fluctuation in the extracellular levels of ATP, other nucleotides and adenosine. These fluctuations in extracellular concentrations are indispensable for the receptor resensitisation after desensitisation following receptor activation as discussed below.

### Purinergic signalling in inflammation and hyperinflammation

The purinergic control of cellular processes including the pro-inflammatory and anti-inflammatory responses of the immune system is depending on the activation and the desensitisation phenomenon of the nucleotides and adenosine receptors of the immune cells [74, 276–282]. Except for the P2X7R, all other purinergic receptors, i.e. P2XRs, P2YRs and P1 receptors (adenosine receptors—AdoRs), are subject to desensitisation [279–283]. In addition, a certain extent of desensitisation occurs after every activation, and this desensitisation requires time to return to the state of complete resensitisation [279, 280]. The higher and the longer the stimulus of the activation, the higher the extent of desensitisation and the longer the recovery time to the state of complete resensitisation [278]. One of the P2 receptors, the P2X7 receptor, is not prone to desensitisation, and apart from the low tonic basal activation of this receptor at low nanomolar concentrations as mentioned above, the extracellular concentration of ATP required to activate this receptor is much higher. Activation of the P2X7R starts at 100 μM with an EC_50_ of >1 mM [74, 279, 284].

**Fig. 3:**
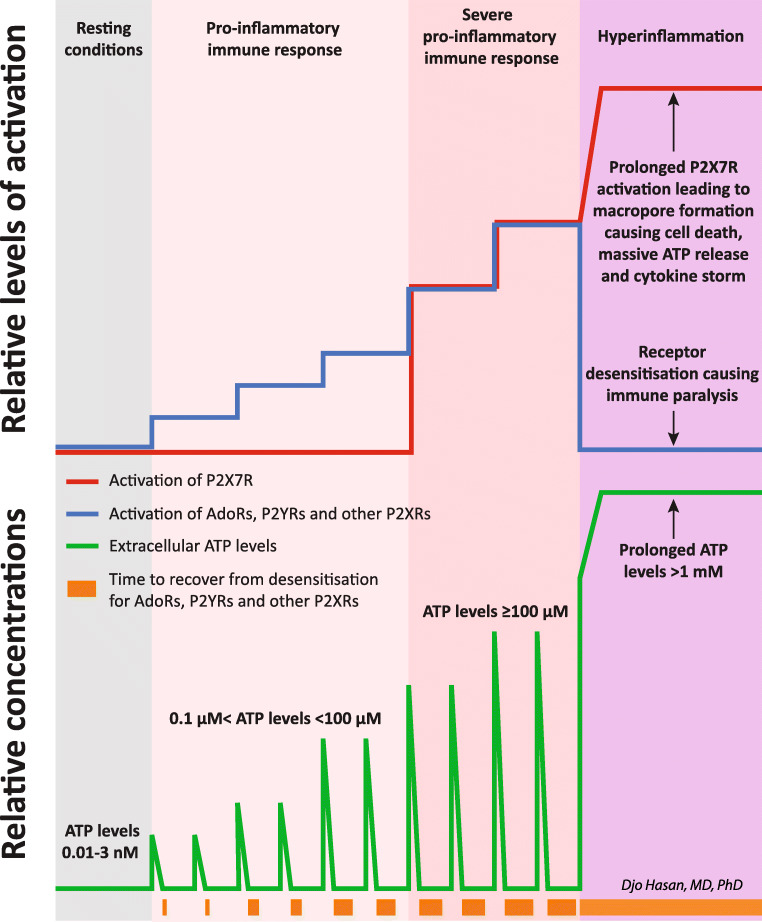
A schematic presentation of the activation of the purinergic receptors of the immune cells causing a pro-inflammatory response leading to hyperinflammation. Viral infection drives the controlled cellular release of ATP molecules. Increased extracellular nucleotides levels activate P2XRs and P2YRs. Upregulation of the extracellular ATP hydrolysing enzymes as depicted in Fig. 2 results in the increase of extracellular adenosine levels followed by the activation of the adenosine receptors (AdoRs). These processes initiate the physiologic pro-inflammatory response of the immune system. The green line at the bottom of the graph represents the extracellular ATP levels. The ascending part is caused by the ATP release*,* and the descending part results from the clearance of ATP by the extracellular or membrane-bound ATP hydrolysing enzymes. As the disease progresses and extracellular ATP levels increase above 1 mM, the P2X7R is additionally and effectively activated leading to a severe immune response. Except for P2X7Rs, all these receptors are known to be subject to desensitisation. Desensitisation of a receptor is defined as being unresponsive to activation by the ligand, resulting in (near) zero transmembrane signal transduction. A certain extent of desensitisation occurs after every activation*,* and this desensitisation requires time to return to the state of complete resensitisation. Increasing intensity and duration of the activation stimuli leads to increasing extent of desensitisation and duration of the recovery time to the state of complete resensitisation (represented by brown boxes with increasing size at the bottom of the graph). Severe viral infection can increase the controlled ATP release beyond the capacity of the extracellular enzymes to clear ATP and adenosine molecules. This causes a sustained high extracellular ATP and adenosine levels preventing the purinergic receptors from recovering from the state of desensitisation. The capacity to clear invading microorganisms diminishes leading to immune paralysis. In addition, prolonged high extracellular levels of ATP and activation of the P2X7R lead to macropore formation and cell death with uncontrolled release of ATP. In turn, this leads to vigorous activation of the P2X7R of the immune cells promoting massive production of cytokines ending in a cytokine storm and hyperinflammation

Summary of the effects of extracellular nucleotides and nucleoside on the innate and adaptive immune system through different purinergic receptors is presented in Table 2, rows 1–163. Coronaviruses can induce inflammation by the activation of the intracellular sensing molecules IRIG1/MDA5 [285, 286]. Reportedly, acute inflammation [69, 70] and infection with SARS-CoV-2 virus induce ATP release [287]. The vesicular exocytosis-mediated release of ATP, connexin-43 (Cx43)-mediated ATP release and pannexin-1 (Panx-1)-mediated ATP release can be triggered by the activation of Toll-like receptor 4 (TLR4) and TLR2 by pathogen-associated molecular patterns (PAMPs) and by the activation of P2X7Rs [180–182, 187]. In turn, activation of the P2X7Rs upregulates the protein expression of TLR 2, TLR3, TLR4 and TLR 5 [288]. Additionally, increased levels of TNF-α during inflammation induce ATP release via Panx-1 [289]. Pro-inflammatory immune response is initiated by the increase in the extracellular ATP, ADP and adenosine levels in the microenvironment of immune cells activating the P2XRs, P2YRs and AdoRs (Fig. 3) [57, 60, 169, 290]. In this case, ATP acts as a danger-associated molecular pattern (DAMP) [291, 292]. Increased ADP levels promote platelet activation and intravascular thrombosis (Table 2, rows 125 and 126). Reportedly, the pathological changes in the lung in patients with COVID-19 pneumonia showed marked microvascular thrombosis [293]. The EC_50_ for AdoRs is in the range of 26 nM to 1.4 μM [281] and for ATP, UTP or ADP receptors (P2XRs and P2YRs with the exception of the P2X7R) in the range of 0.01 nM to 10 μM [284, 294]. Obviously, the extent of the cellular ATP release is proportional to the severity of the infection. A severe infection with SARS-CoV-2 causes massive extracellular ATP release by the infected cells. This may be confined to the airway mucosa and the lung or may be extensive in multiple organs. Although increased extracellular ATP concentrations upregulate the expression of ecto-nucleotidases [295] these high ATP concentrations exceed the capacity of these ecto-enzymes (CD39, CD73, etc.) to clear the extracellular space from ATP molecules [60] ending in ATP concentrations of >1 mM. This is demonstrated in a report where the authors show that TLR-mediated CD39 internalisation (causing the deactivation of the ecto-enzyme CD39) in mice bone marrow-derived dendritic cells (BMDCs) leads to the accumulation of extracellular ATP to 1.4 mM [296]. The activation of P2X7Rs in ongoing inflammation is the hallmark of severe pro-inflammatory immune response (Table 2, rows 74, 86–119 and Fig. 3) [297] including COVID-19 [298]. If these levels of extracellular ATP are accompanied by the absence of the required fluctuations for other purinergic receptor to recover from desensitisation, all P1 and P2 (other than P2X7) purinergic receptors will become fully desensitised demarcating the initiation of hyperinflammation (Fig*.*3 and Table 2, rows 120–123) [279–283].

#### Hyperinflammation is characterised by the activation of P2X7Rs and desensitisation of other P2 receptors and AdoRs

As mentioned above, hyperinflammation starts when fluctuation of the extracellular nucleotides and adenosine no longer occurs and leads to prolonged activation of the P2X7Rs of the immune cells. Prolonged vigorous activation of the P2X7Rs leads to macropore formation and cytolysis with uncontrolled ATP release [222, 223, 227, 299] (Table 2, rows 120*–*123) causing hyperinflammation with massive pro-inflammatory immune response, massive pro-inflammatory and anti-inflammatory cytokine release: the cytokine storm (Fig. 3). In the early phase of COVID-19, hyperinflammation may be confined to the site of viral entry (i.e. airway mucosa and conjunctivae) but as viral replication and viral spreading progress, systemic hyperinflammation devel ops.

The upregulation of the expression of ectonucleotidases also leads to an increase in the concentrations of other nucleotides (i.e. ADP) and adenosine. These high extracellular concentrations of nucleotides and adenosine do not show concentration fluctuations required for the recovery (resensitisation) time from desensitisation causing a state of persistent desensitisation of all P2XRs, P2YRs [279, 280, 283, 300, 301] and AdoRs [282] with the exception of P2X7Rs. Consequently, the physiological function in the affected organs and inflamma tory response of the immune system are deactivated. This leads to the failure of organ function (i.e. ARDS in the lungs as we reported earlier [61]) and the immune system (immune paralysis) rendering the host susceptible to secondary co-infections(Fig*.*3). Sepsis-induced immunosuppression [302, 303] or compensatory anti-inflammatory response syndrome (CARS) in critically ill patients [304] was already raised by researchers in 1996 [305] and is a well-known phenomenon in critically ill patients [302]. Secondary bacterial infections occurred in 34.4% of 274 surviving elderly patients (age over 60 years) with COVID-19 and in 81.7% of 65 deceased patients [15]. In addition, it was found that 76 co-infections with other respiratory pathogens occurred in another cohort of 354 COVID-19 patients (16 of 115 mild cases (13.9%), 33 of 155 severe cases (21.3%) and 27 in 84 critical cases (32%)) [16]. In a meta-analysis involving 118 scientific reports on patients with COVID-19, co-infection with other pathogens at admission was observed in 19% and superinfection with other pathogens during admission in the hospital in 24% [306].

#### Control of hyperinflammation is annihilated by the downregulation of Tregs through the activation of P2X7R and the desensitisation of adenosine receptors

Tregs are key elements in the control of hyperinflammation [307]. Activation of AdoRA2As promotes the differentiation of naïve T-cells towards regulatory T-cells(Tregs) [112], increases the frequency of Tregs and the expression of CTLA-4 receptor and upregulates ecto-enzymes CD39 and CD73 expression accelerating adenosine generation from extracellular ATP [118] (Table 2, rows 25, 29, 30 and 33). This process is upset in case of desensitisation of AdoRs. In addition, activation of P2X7Rs inhibits the suppressive potential and stability of Tregs, inhibits the clonal expansion of Tregs, promotes Treg death, induces Treg depletion and reduces Treg IL-10 production (Table 2, rows 86*–*88, 106 and 107). In COVID-19 patients, significant lower Treg frequencies [308–310], lower expression of forkhead box protein P3 (FoxP3), lower expression of transforming growth factor-β(TGF-β) and lower cytokine TGF-β secretion [309] are observed compared to healthy control. Additionally, a reduced proportion of specific SARS-CoV-2-reactive Tregs was reported [311]. The desensitisation of AdoRs and the activation of P2X7Rs may well be the underlying mechanism of the low Tregs frequency in severe and critically ill COVID-19.

### P2X7R antagonist restores the reduced Tregs population and Tregs function in hyperinflammation

As stated above, infected cells release ATP into the extracellular space. Obviously, the P2X7R antagonist blocks the activation of the P2X7Rs. Because a significant proportion of the ATP release to the extracellular space is mediated by the P2X7R (Table 2, rows 77–79), P2X7R a ntagonism combined with the upregulated ATP hydrolysing activity of the ecto-enzymes results in the decrease of the extracellular ATP concentrations. This can potentially abrogate hyperinflammation and the concomitant immune paralysis. Moreover, P2X7R inhibition promotes the cell-autonomous conversion of CD4+ T cells into Tregs after stimulation of their T-cell receptors (TCRs) [190]. In addition, P2X7R knock-out mice, mimicking the state of complete P2X7R inhibition, show an increase in tissue Tregs, prevent Tregs death and the Tregs produce more IL-10 and TGF-β [191]. Experimental inhibition of P2X7Rs restores the Tregs levels and function (Table 2, rows 86*–*88, 106 and 107) [190–192]. Inhibition of the P2X7R or P2X7R knock-out can attenuate severe inflammation in abdominal sepsis [312] and in acute lung injury [313, 314]. Apparently, amelioration of hyperinflammation by P2X7R inhibition is based on the increased activation and clonal expansion of the anti-inflammatory Tregs population (Table 2, rows 86*–*88, 106 and 107).

Some authors proposed that the P2X7R is an ideal candida te to target in COVID-19-associated severe pneumonia [298, 315], and others suggested that hyperactivation of the P2X7R plays a key role in the neuropathology of COVID-19 and that P2X7R antagonism may prevent or treat neurological manifestations of COVID-19 [316].

#### Lidocaine is a P2X7R antagonist

In 2015 it was discovered that lidocaine is a P2X7R antagonist [74], and therefore, lidocaine can potentially reduce the clinical symptoms of hyperinflammation significantly. In experimental sepsis, lidocaine improves organ failure [317–319] and survival [317]. In septic patients, lidocaine reduces neutrophil recruitment by the mitigation of chemokine-induced arrest and transepithelial neutrophil migration [320]. Neutrophil recruitment is an important facilitating process in the pathogenesis of multiple organ failure [320] and hyperinflammation in COVID-19 [321–324]. In patients with skin lesions from atopic dermatitis, lidocaine increases the proportion of Tregs and upregulates the FoxP3 expression [325]. In addition, lidocaine increases the IL-10 levels in mechanically ventilated mice [326] and decreases the TNF-α in BAL, plasma and lung samples in pigs undergoing surgery for lung resection [327].

**Fig. 4:**
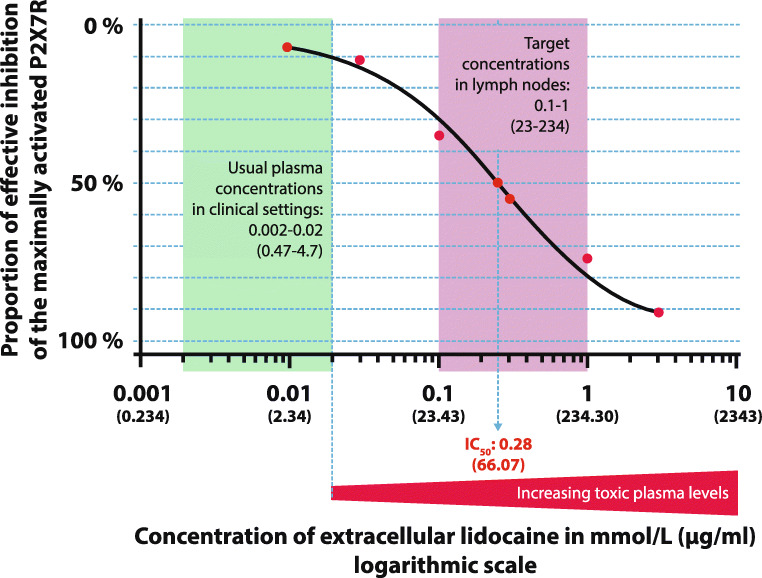
Dose-response relationship of lidocaine suppressing the ATP-induced currents in oocytes expressing P2X7R. We reconstructed the fitted curve from the inhibitory concentrations data of lidocaine for P2X7R from the original article: 7% inhibition: 0.01 mM (2.34 μg/ml); 11% inhibition: 0.03 mM (7.03 μg/ml); 35% inhibition: 0.10 mM (23.43 μg/ml); 50% inhibition (IC_50_): 0.28 mM (66.07 μg/ml); 55% inhibition: 0.30 mM (70.29 μg/ml); 74% inhibition: 1.00 mM (234.30 μg/ml); 91% inhibition: 3.00 mM (702.90 μg/ml); and 98% inhibition: 10.00 mM (2343.00 μg/ml), respectively. The usual plasma concentrations in clinical settings are indicated by the green box, and the targeted concentrations in the lymph nodes are indicated by the magenta box. Note that the maximal tolerable plasma levels for human (about 4.7 μg/ml*–*0.02 mmol/L) are much lower than the required extracellular concentrations of lidocaine to effectively inhibit the P2X7R. Source: Okura D, et al. [74]

The P2X7R antagonist dose-response relationship of lidocaine is presented in Fig*.*4. The IC_50_ for the inhibition of the P2X7R by lidocaine is about 66.07 μg/ml (0.28 mM) [74] where IC_50_ is defined as the required extracellular concentrations of the receptor antagonist to reach an inhibitory effect of halfway between maximal activation and maximal inhibition (half-maximal inhibitory concentration). The main issue is that the IC_50_ for P2X7R inhibition is much higher than the maximal tolerable plasma concentration for mammals. The maximal tolerable plasma concentration in humans is about 4.7 μg/ml (0.02 mM); this corresponds with an IC_10_ or lower (<10% inhibitory concentration, Fig*.*4). Above this lidocaine plasma concentration, adverse effects in increasing severity occur as presented in Table 3 [328, 329]. Thus, systemic lidocaine plasma concentrations of >4.7 μg/ml must be avoided [328, 329]. Caveat: The inhibitory concentrations of lidocaine for P2X7R as presented in Fig*.*4 are not corrected for the series resistance (in the range of 1–3 MΩ) of the used whole-cell voltage clamp method with two puller microelectrodes [74]. One should bear in mind that after correction for series resistance, the reported inhibitory concentration values including IC_50_ are expected to be higher [330].

**Table 3:** Adverse effects in relation to the plasma concentrations of lidocaine. The maximal tolerable plasma levels for human are about 4.7 μg/ml (0.02 mmol/L). Serious adverse effects start at 9.84 μg/ml (0.042 mmol/L). Source: Hermanns H et al. [328] and Weinberg L et al. [329]

Symptoms of toxic plasma levels of lidocaine	Lidocaine concentration		
	mmol/L	μmol/L	μg/ml
No noticeable symptoms	<0.020	<20	<4.69
Anxiety, dizziness	0.020	20	4.69
Decreased spinal reflexes	0.042	42	9.84
Central nervous system (confusion, diplopia, nausea and vomiting, twitching and tremors, seizures with reduced consciousness, respiratory depression, coma, etc.)	0.080	80	18.74
Cardiac toxicity (bradycardia, hypotension, cardiovascular depression, cardiac arrest, etc.)	0.130	130	30.46
Cytotoxicity	3.0	3000	702.9

In addition to the P2X7R antagonist properties, lidocaine is also known to have several other inhibitory pharmacological targets: the voltage-gated sodium channels (VGSC: Nav1.2 [331], Nav1.3 [332], Nav1.4[333], Nav1.5 [334], Nav1.7 [335], 1.8 [336] and Nav 1.9 [337]), the Toll-like receptor 2 (TLR 2) [338], TLR4 [318] and the N-methyl-D-aspartate receptor (NMDAR) [339].

VGSCs conduct sodium ions inward and are essential for the transduction of sensory stimuli, the generation of the action potential and the release of neurotransmitters from sensory neuron terminals. Lidocaine inhibition of VGSCs can effectively reduce pain signalling [340]. In addition, VGSCs are present on dendritic cells (maintain chemokine-induced migration) [341], macrophages (regulate phagocytosis and endosomal pH during LPS-mediated endosomal acidification) [342], microglia (regulate phagocytosis cytokine release ad migration) [343], neutrophils (regulate attachment, transmigration and chemotaxis) [344] and T-cells (regulate positive selection of CD4^+^ T cells) [345]. However, until date no relevant data have been published suggesting that other VGSC antagonists (such as HYP-17 [346], A-803467 [347, 348], PF-05089771 [349], phenytoin [350] or tetrodotoxin [351, 352]) may substitute non-steroidal anti-inflammatory drugs let alone may suppress COVID-19-related hyperinflammation [353]. A plausible reason is that during hyperinflammation—including hyperinflammation in COVID-19—the cytokine levels (i.e. IL-1β [354], IL-6, IL-10 [355, 356] and IL-12 [357]) are high. Reportedly, IL-1β [358] and IL-6 [359] inhibit sodium currents of VGSCs, and IL-10 downregulates the expression of VCSCs [360]. Moreover, activation of the P2X7R reduced the density and currents of VGSCs [361]. Therefore, we do not consider the inhibitory properties of lidocaine on VGSCs to be relevant for the treatment of hyperinflammation in COVID-19.

At first glance, the downregulation of the expression of TLR 2 [338] and TLR 4 [318] is an important anti-inflammatory mechanism directly induced by lidocaine. But at a closer look, it appeared that activation of P2X7R by the agonist cathelicidin (LL-37) leads to the upregulation of the protein expression of TLR2, TLR3, TLR4 and TLR 5 [288]. This is in line with the MyD88 (myeloid differentiation primary-response protein 88)-dependent activation of NF-κB (nuclear factor kappa-light-chain-enhancer of activated B cells) following the activation of the P2X7R by BzATP [362, 363]. The MyD88-dependent activation of NF-κB is part of the TLR4/NF-κB pathway. Therefore, it is unsurprising that the inhibition of P2X7R by its antagonists (Brilliant Blue G, A-438079 and A-740003) neutralises the above-mentioned P2X7R-induced upregulation of TLRs [362]. Consequently, we argue that lidocaine inhibits inflammation directly by blocking P2X7Rs independent from the neutralisation of the P2X7R-induced upregulated TLR2 and TLR4.

The subpopulation of NMDA receptors present on the peripheral neurons are involved in nociception, and their number increases during inflammation contributing to the sensitisation of peripheral nerves to nociceptive stimuli. NMDA receptor antagonists have anaesthetic-like effects [364]. In addition, NMDA receptor antagonist can prevent hypoxic neuronal death, IL-1β and TNFα release [365], reduce the activation of inflammatory experimental colitis [366] and suppress glial pro-inflammatory cytokine expression [367]. Moreover, the NMDA receptor antagonist memantine can increase IL-10 production in BCR/CD40-activatedB-cells [368]. Lidocaine inhibits NMDA receptors [339, 369, 370], and thus the anti-inflammatory properties of lidocaine could be attributed to the inhibition of NMDA receptors. However, it has been reported that the anti-inflammatory effect in T-cell functions (inhibition of antigen-specific T-cell proliferation, T-cell cytotoxicity, T-cell migration towards chemokines and decrease in IL-2 and IFN-γ production by Th1 effector cells in favour of IL-10 and IL-13 production by Th2 cells) of the NMDA receptor antagonist ifenprodil is effective both in wild-type and in NMDA receptor (GluN1) knockout mice [371]. Moreover, it was found that KN-62, an inhibitor of Ca2+/calmodulin-dependent kinase type II and a potent P2X7R antagonist, provides neuroprotection against NMDA-induced cell death [372]. Therefore, we argue that the anti-inflammatory properties of NMDA receptor antagonists (including lidocaine) should be attributed to the inhibition of P2X7Rs rather than to the inhibition of NMDA receptors.

#### Selective inhibition of the P2X7Rs of the immune cells in the lymphatics avoids exceeding the maximal tolerable plasma concentration of lidocaine and inhibits hyperinflammation in two stages

As mentioned above, the main issue is that the IC_50_ for P2X7R inhibition is much higher than the maximal tolerable plasma concentration for mammals because P2X7Rs are indispensable for normal physiological functions (i.e. in the central nervous system [373], the peripheral nervous system [374] and in the lungs [60, 61]). Therefore, intravenous or oral administration aimed at achieving an effective concentration of lidocaine to inhibit P2X7Rs in serum and in target organs will hamper organ functions and is potentially dangerous.

The lymphatic system is populated exclusively by trafficking immune cells, i.e. naïve T cells, activated T cells, B cells [375], dendritic cells [376], monocytes [377], macrophages [378], neutrophils [379], mast cells [380], eosinophils [381] and basophils [382]. We postulate that selective inhibition of the P2X7Rs of the immune cells of the lymphatic system by lidocaine suppresses hyperinflammation in two stages: stage 1, the selective inhibition of the P2X7Rs of the immune cells residing in the lymph nodes induces clonal expansion of Tregs in these lymph nodes; stage 2, subsequently, these Tregs migrate throughout the body exerting anti-inflammatory activities reducing systemic and (distant) local hyperinflammation (Fig. 1).

The endothelium of the dermal capillaries of the skin belongs to the structural type “continuous endothelium” [383]. Although capillary walls can transport substances from blood to tissue, the absorption of substances from tissue to blood is, if any, extremely low [384]. Apparently, specialised initial lymphatics harbouring one-way valve leaflets capable of absorbing fluid and molecules from the interstitium are localised in the dermis. The absorbed lymph fluid is then propelled forward in the lymphatic network by collecting lymphatic vessels harbouring a rhythmic contracting muscle layer [385]. This system brings fluids and particles into the lymph nodes where numerous immune processes take place. The administration route to target the lymphatic system in a domestic swine model is illustrated by the subcutaneous or intradermal injection of compounds (isosulfan blue, fluorescein and radioactive technetium-99 isotope—Tc^99^) and by tracing the extent and the transit time of the distribution of these compounds using whole body scintigraphy in pigs [386]. The absorption of intradermal application of radioactive Tc^99^ into the lymph nodes is 10 times faster than after deep subcutaneous application and leads to higher concentrations in the lymph nodes related to these lymphatic vessels [386]. Radionuclide lymphoscintigraphy with molecules of different sizes after intradermal and subcutaneous injections showed that smaller particles (i.e. 99mTc-dextran and 99mTc-human serum albumin) migrate more rapidly towards the lymphatic vessels and lymphatic nodes than larger particles (i.e. radiocolloids of larger molecular size) [387]. The rate of clearance of ^99m^Tc-pertechnetate and ^99m^TcDTPA after subcutaneous and intradermal administration in the back of the hand in humans is 1 %/min and 8 to 10 %/min, respectively [387].

The additional advantage is that the plasma concentrations of subcutaneously administered lidocaine are much lower than intravenously administered lidocaine. Intravenous administration of 2 mg/kg lidocaine in cats is almost immediately followed by a peak plasma concentration of 3.6 μg/mL [388]. In contrast, the achieved mean peak plasma concentrations after the subcutaneous administration of 30 mg/kg, 20 mg/kg and 10 mg/kg lidocaine are much lower: 1.69, 1.07 and 0.77 μg/mL, respectively [389]. Note that the applied subcutaneous dose [389] is 15, 10 and 5 times higher than the intravenous dose, respectively [388, 389]. Reportedly, the difference in the plasma concentrations after intravenous and subcutaneous administration of lidocaine is caused by the fact that, in contrast to the intravenous administration, a large proportion of the subcutaneously administered lidocaine is drained into the lymphatic system [390–392]. Obviously, this slows down the release of lidocaine to the venous blood. This is confirmed for bevacizumab in mice [390], for trastuzumab in rats [391] and for docetaxel in rats by [392].

**Fig. 5:**
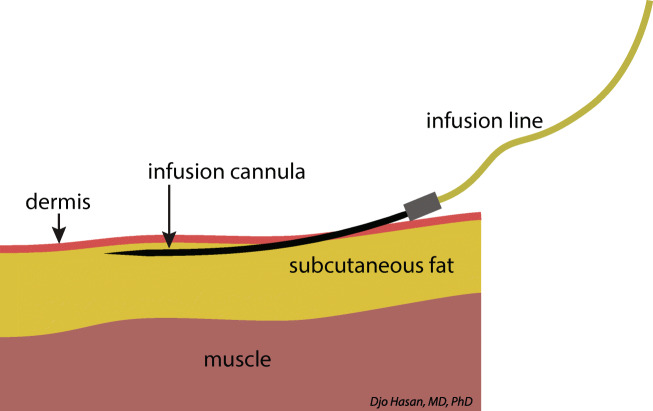
*The cannula for subdermal infusion of lidocaine is superficially positioned just below the dermis* to promote the uptake of lidocaine by the initial lymphatics of the dermis and to avoid accumulation of lidocaine in the subcutaneous fat tissue

**Fig. 6:**
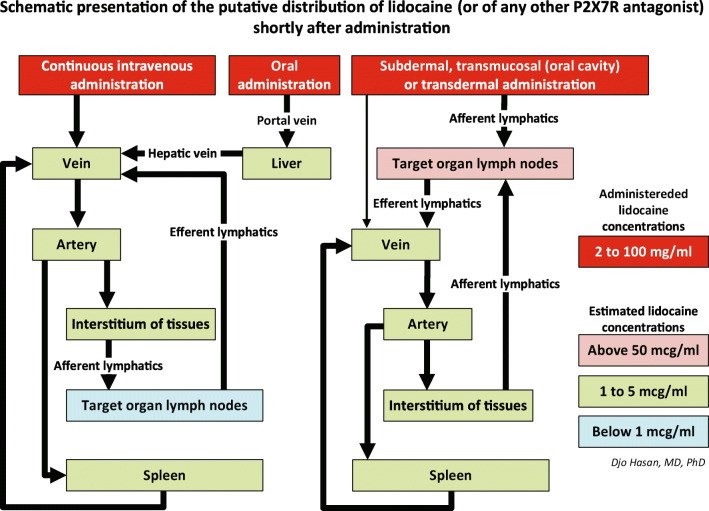
*Schematic presentation of the putative distribution of intravenous*, oral, transmucosal (i.e. in the oral cavity) and subdermal administered lidocaine. Administration of hydrophilic lidocaine (lidocaine HCL) through a (central) venous catheter or by oral intake results in concentration gradients with the highest value in the venous blood and the lowest value in the lymph nodes. The reason is that by the time lidocaine reaches the lymph nodes*,* the drug is massively diluted and may never reach the effective concentration required to adequately inhibit the P2X7Rs of the immune system. In contrast, after subdermal injection of hydrophilic lidocaine, apart from a minimal absorption by the dermal capillaries, almost all the lidocaine is absorbed by the lymphatic system via the initial lymphatics. Because the fluid in the afferent collecting lymphatics originates from the interstitial fluid of the tissues, dilution of the concentration of lidocaine occurs. This fluid is then drained into the local lymph nodes. The extent of the dilution of lidocaine in the targeted lymph nodes is far less drastic compared to the (central) venous administration of the drug. We postulate that with continuous subdermal infusion*,* we can achieve concentrations of lidocaine in the lymph nodes sufficient to effectively inhibit the P2X7Rs of the immune cells. Theoretically, similar results may be expected from transmucosal and transdermal administration of lipophilic lidocaine base with a high concentration. Obviously, the subdermal, transmucosal and transdermal administration routes may also apply to other P2X7R antagonists

As stated above, lymphatic absorption after intradermal administration is much higher than after deep subcutaneous administration [386, 387]. Practically, the intradermal infusion with lidocaine is not an accepted administration route for lidocaine. Therefore, we argue that a subdermal administration of lidocaine using a catheter inserted just beneath the dermis (subdermal infusion, Fig. 5) will result in higher concentrations of lidocaine in the draining local lymph nodes than a deep subcutaneous or intravenous infusion as depicted in the schematic presentation of the putative distribution of lidocaine in Fig. 6.

In summary, by means of the subdermal administration of lidocaine, we can ensure high concentrations of lidocaine in the local lymph nodes enabling an effective inhibition of the P2X7R of the immune cells while keeping the lidocaine plasma concentrations <4.7 μg/ml(stage 1a and 1b in Fig. 1). The induced Tregs clonal expansion in these local lymph nodes produces Tregs which migrate throughout the body controlling the ongoing hyperinflammation (stage 2 in Fig. 1). Obviously, the subdermal administration route may also apply to other P2X7R antagonists.

Three other P2X7R antagonists have been tested in human: CE-224,535 500 (Pfizer), AZD-9056(Astra-Zeneca) and JNJ-54175446 (Johnson and Johnson). A phase IIa study with CE-224,535 in patients with rheumatoid arthritis not responding adequately to methotrexate was recently reported [393]. Patients in the treatment arm received oral CE-224,535 500 mg twice/day for 12 weeks. Although the safety and tolerability for the compound were acceptable, CE-224,535 was not effective in this group of patients. The results of a phase II study with AZD-9056 in patients with active rheumatoid arthritis despite treatment with methotrexate or sulphasalazine was published. The treatment arm consists of oral AZD-9056 100 or 400 mg/day for 6 months [394]. The AZD-9056 used in this trial is non-lipophilic as indicated by the fact that this compound cannot penetrate the blood-brain barrier [395]. The authors conceded that “AZD-9056 does not have significant efficacy in the treatment of RA, and the P2X7 receptor does not appear to be a therapeutically useful target in RA” [394]. Recently, a randomised, placebo controlled, sequential-group, single-centre ascending dose phase I study was reported. The patients in the 5 treatment arms received 0.5, 2.5, 10, 50, 150 and 300 mg JNJ-54175446, respectively. The authors reported dose-dependent plasma levels, no serious adverse events, ex vivo attenuation of lipopolysaccharide-induced IL-1β release in peripheral blood and confirmation of passive brain penetration of JNJ-54175446 [396]. The approach of the P2X7R antagonist therapy of the above-mentioned authors is quite different from ours: While these authors directly targeted the diseased organs via the gut absorption of the drug, we target the immune cells in local lymph nodes inducing an anti-inflammatory immune response which in turn targets the diseased organs (Fig. 1). This is illustrated by the following study concerning a placebo-controlled, multicentre, double-blind phase IIa study in patients with moderately to severely active Crohn’s disease. The patients in the treatment arm received oral AZD-9056 200 mg/day for 28 days. The authors found a significant improvement in the Crohn’s Disease Activity Index (CDAI) at day 28 [397]. In contrast to the skin, the endothelium of the mucosal capillaries of the mouth and the gastrointestinal tract are fenestrated allowing molecules to pass from the submucosal tissue into the capillaries [383]. Unlike the failure of the treatment of rheumatoid arthritis described above, the successful treatment of gut inflammation here can be attributed to the absorption of non-lipophilic oral AZD-9056 by the mucosa-associated lymphoid tissue (MALT). This is the inductive site of the mucosal immune system consisting of mesenteric lymph nodes, Peyer’s patches and isolated lymph follicles [398, 399]. Although lymphatic transport to the lymph nodes of the non-lipophilic oral AZD-9056 is limited [400, 401], AZD-9056 inhibits P2X7Rs of the local T-cells via absorption by the inductive sites of MALT. This induces a local anti-inflammatory immune response executed by the effector sites of MALT consisting of lamina propria lymphocytes and intraepithelial lymphocytes [398, 399].

## Real-world subdermal administration of lidocaine in critically ill COVID-19 patients

### Six COVID-19-induced ARDS patients

**Fig. 7:**
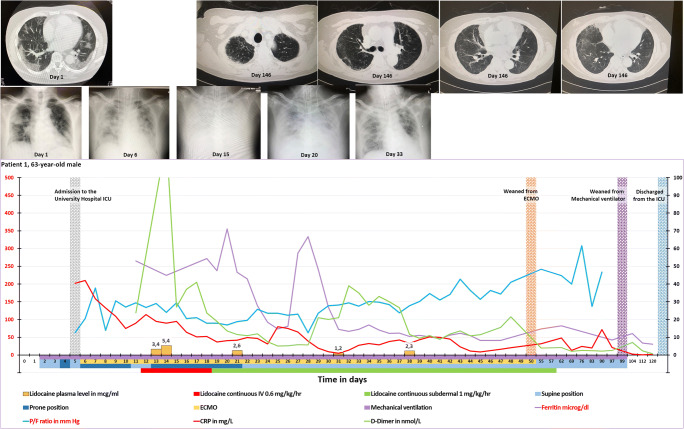
Patient 1, the first of the six cases with severe COVID-19 treated with subdermal lidocaine in the ICU of the Showa University, Tokyo, Japan. A 63-year-old male with COVID-19-induced ARDS, was admitted to the hospital. The CT scan showed bilateral ground glass opacities. Co-morbidities: COPD, smoking 60 cigarettes per day for more than 40 years. About 40 years before admission*, the patient suffer*ed from pneumothorax. After admission the clinical condition deteriorated requiring an ICU admission and mechanical ventilation on day 4. On day 11*, continuous intravenous lidocaine of 0.6 mg/kg/h* was initiated*, but the patient’*s condition kept worsening with high pulmonary artery pressures and reduced aeration of the lung. On day 19*, the continuous intravenous lidocaine of 0.6 mg/kg/h* was changed to continuous subdermal lidocaine of 1 mg/kg/h. This was followed by improvement of the clinical condition*, and* on day 20*, the aeration of the lung was improved, but the pulmonary artery pressures remain*ed high. Despite this the P/F ratio was gradually improving*, and ECMO weaning was done on day 50. No new ECG changes were observed during treatment with lidocaine. Bloo*d metHb were within the normal range (0.3*–0.8%).* On day 99*, he was weaned from the mechanical ventilator and was discharged from the ICU on day 121.* CT scan on day 146 showed reduced ground glass opacities in both lungs and some interstitial change in upper and middle fields of the lung and improvement of the pneumothorax. The patient was discharged from the hospital on day 187, he went home, and he could walk but needed extra oxygen supply of 2L/min. Nine months after admission, the patient is doing well and has returned to work. The patient visited the hospital 3 months after discharge: He only uses oxygen 1 L/min to go shopping and during physical training (out-patient rehabilitation). He talked to the treating intensivist without requiring oxygen and had no shortness of breath or tachypnoea. The red-coloured labels of the legends refer to graph plots using the (left) primary *Y*-axis, and the black-coloured labels of the legends refer to graph plots using the (right) secondary *Y*-axis

**Fig. 8:**
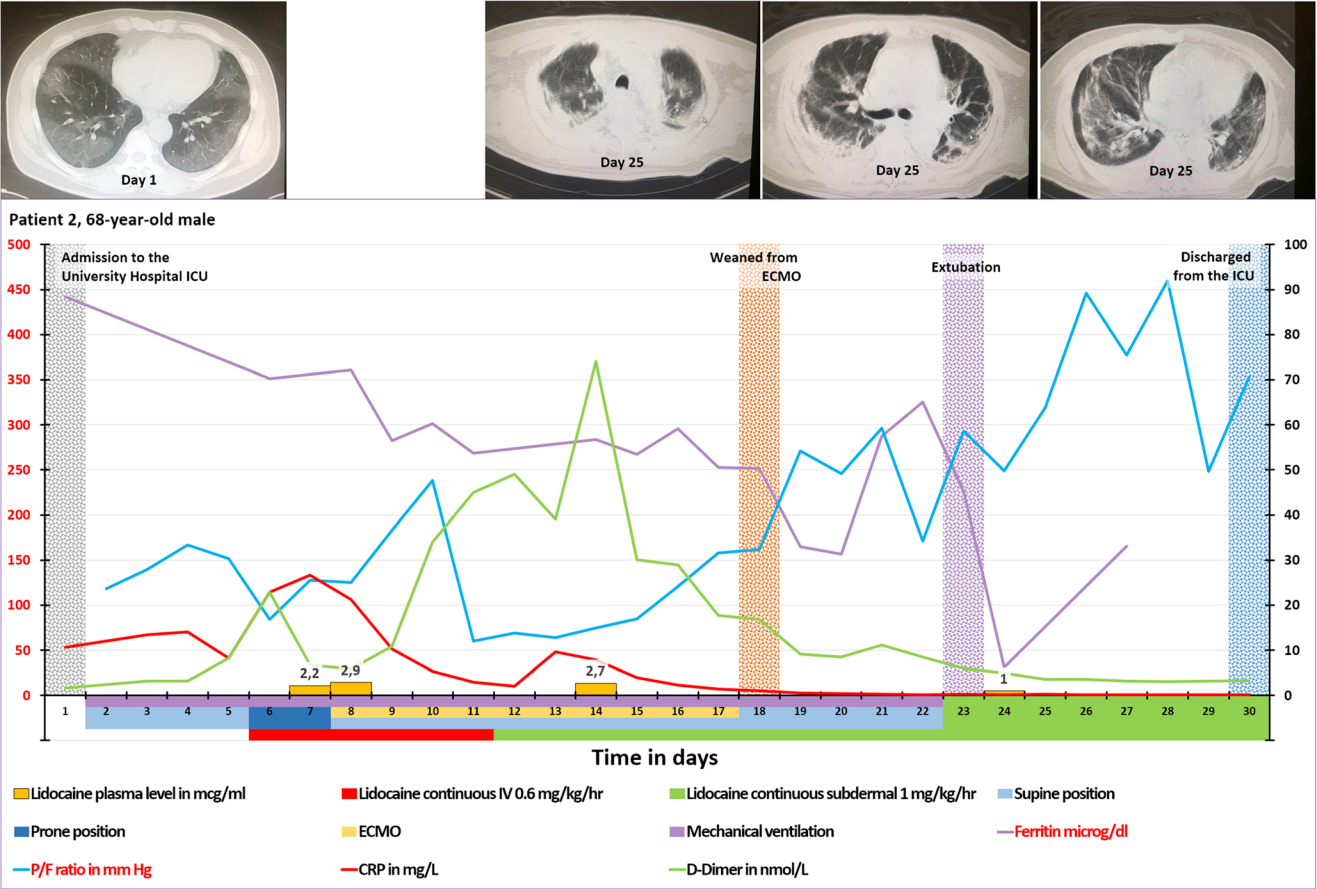
Patient 2. A 68-year-old male with COVID-19-induced ARDS admitted to the ICU and required mechanical ventilation. The CT scan showed bilateral ground glass opacities. Co-morbidity: Asthma. After admission the patient*’*s condition was deteriorating. On day 5*, continuous intravenous lidocaine of 0.6 mg/kg/h* was initiated, but the clinical condition and the P/F ratio kept worsening. On day 11*, the intravenous lidocaine of 0.6 mg/kg/h* was changed to continuous subdermal lidocaine of 1 mg/kg/h. A few days later*, this was followed by improvement of the clinical condition and the P/F ratio. No new ECG changes were observed during treatment with lidocaine. Blood metHb were within the normal range (0.1–*0.6%). The patient was discharged from the ICU on day 30 home on day 37. At *3* months after admission*, the patient is doing* well. The red coloured labels of the legends refer to graph plots using the (left) primary *Y*-axis, and the black-coloured labels of the legends refer to graph plots using the (right) secondary *Y*-axis

**Fig. 9:**
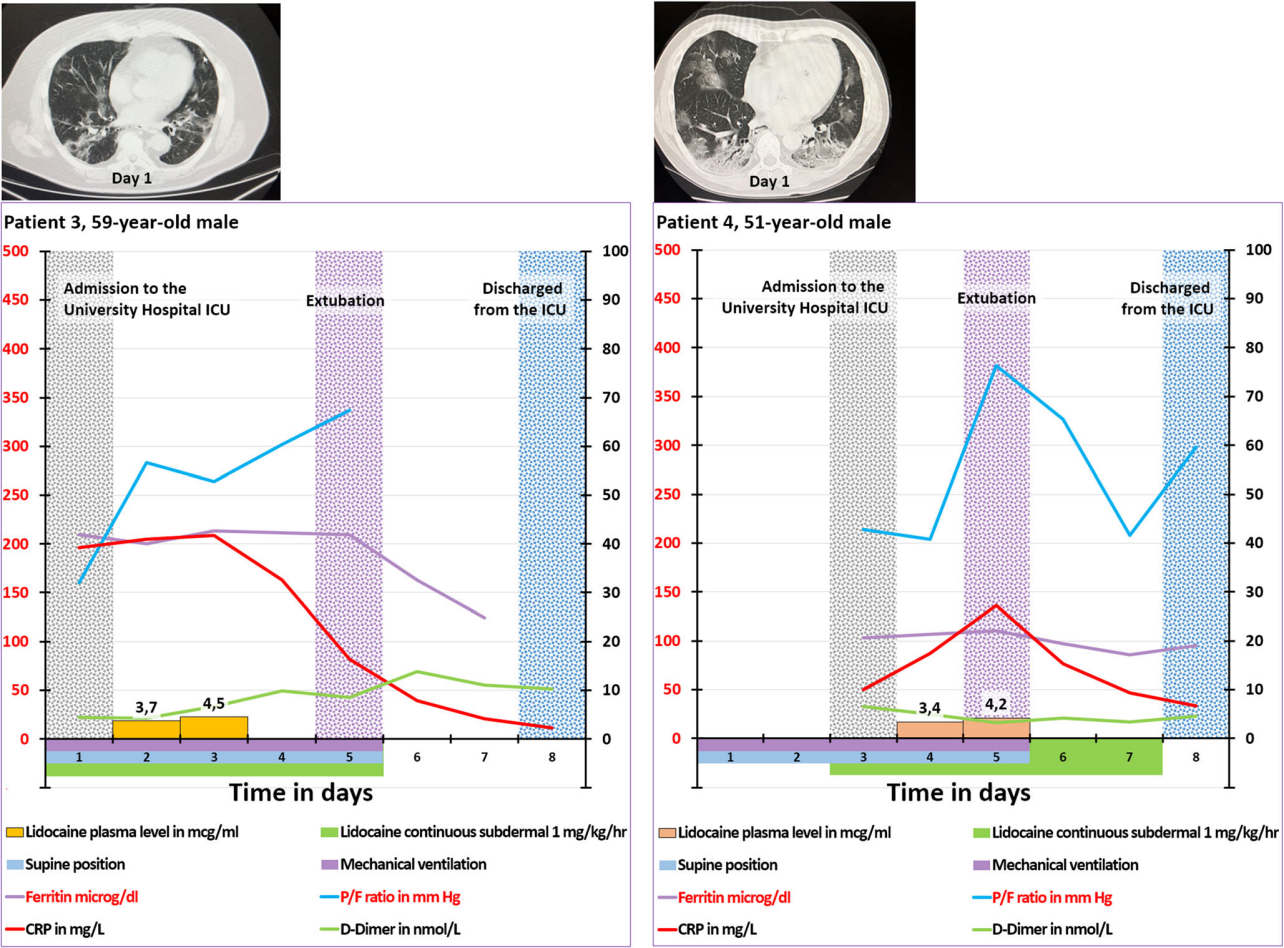
Left graph: Patient 3. A 59-year-old male with respiratory distress and bilateral ground glass opacities on the CT scan. Co-morbidity: Obesity, diabetes mellitus and gout. No new ECG changes were observed during treatment with lidocaine. Blood metHb were within the normal range (0.1*–0.4%).* The patient was discharged from the ICU on day 8 and was discharged home on day 20. After *3* months*, he is* doing well. Right graph: Patient 4. A 51-year-old male with fever, dyspnoea and cough due to COVID-19. The CT scan showed bilateral ground glass opacities. Co-morbidity: none. No new ECG changes were observed during treatment with lidocaine. Blood metHb were within the normal range (0.1*–*0.3%). The patient was discharged from the ICU on day 8 and was disc harged home on day 28. At *3 months, he is doing well and has returned to work.* The red-coloured labels of the legends refer to graph plots using the (left) primary *Y*-axis, and the black-coloured labels of the legends refer to graph plots using the (right) secondary *Y*-axis

**Fig. 10:**
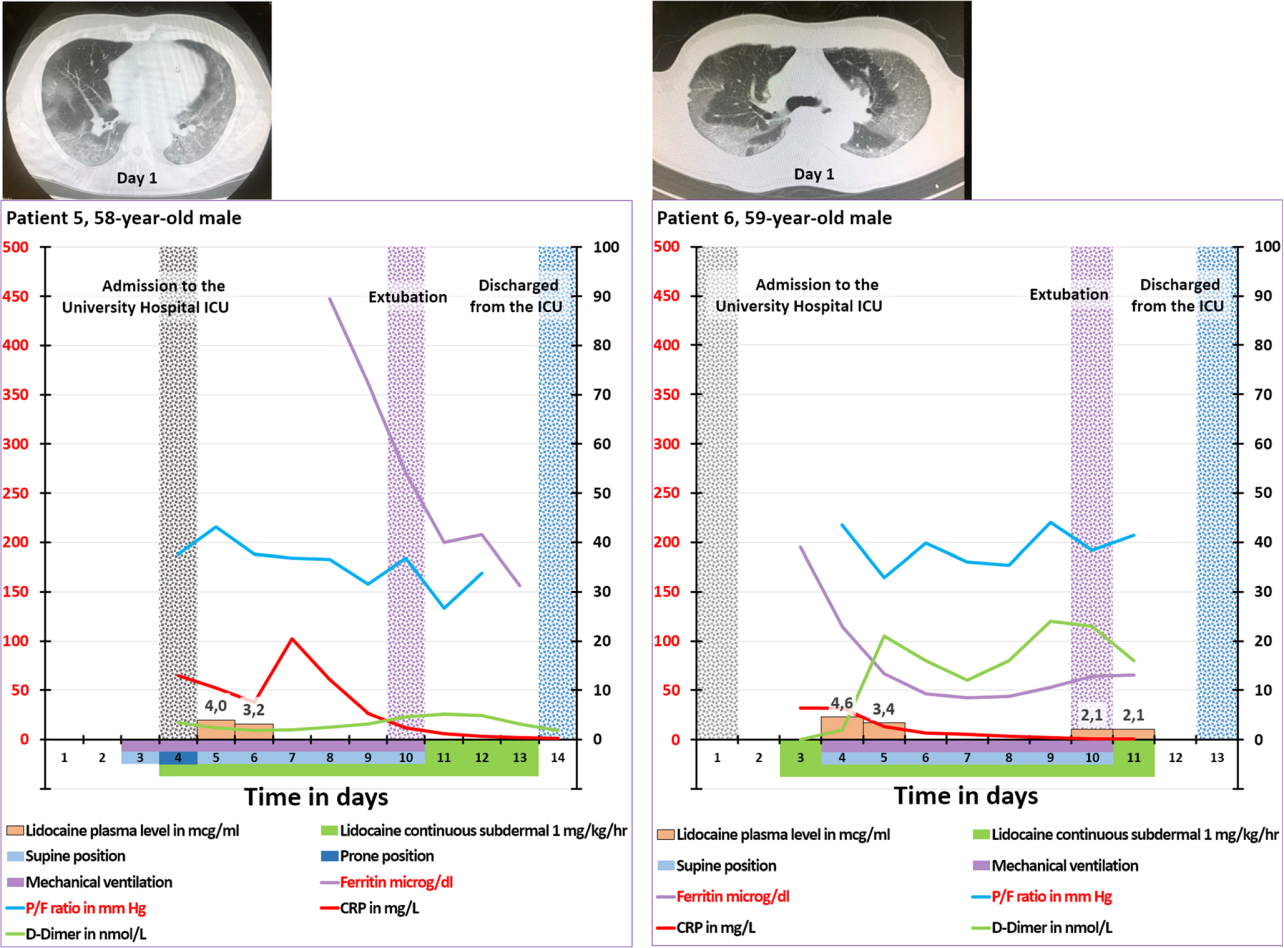
Left gr aph:  P atient 5. A 5 8 -year-o ld male with fever, dyspnoea and cough due to COVID-19. The CT scan showed bilateral ground glass opacities. Co-morbidity: Fatty liver. No new ECG changes were observed during treatment with lidocaine. Blood metHb were within the normal range (0.1*–0.3%).* On day 14*, the patient was discharged from the ICU. On day 20, the patient was* discharged home and is doing well at *3 months after admission.* Right graph: Patien ts 6. A 59-year-old male with fever, dyspnoea and cough due to COVID-19. CT scan showed bilateral ground glass opacities. Co-morbidity: Hypertension on medication. No new ECG changes were observed during treatment with lidocaine. MetHb were within the normal range (0.1*–0*.3%). On day 13*, the patient was discharged from the ICU. He was discharged from the hospital on day 20, and a t 3* months after admission*, he is doing well, played golf* regularly and has returned to work. The red-coloured labels of the legends refer to graph plots using the (left) primary *Y*-axis, and the black-coloured labels of the legends refer to graph plots using the (right) secondary *Y*-axis

From April 2020 until end of July 2020, two of the authors of this report (AS and TK) have successfully treated six critically ill patients with COVID-19 admitted to the ICU of the Showa University in Tokyo, Japan, with lidocaine. The lidocaine treatment was based on off-label use. The Medical Ethical Committee of the Showa University, School of Medicine, Tokyo, approved the collection, analysis and publication of patients on mechanical ventilation admitted to the ICU (protocol number 3313). The administration was initially intravenously in the two first patients, followed by subdermally (a superficially inserted subcutaneous catheter as illustrated in Fig. 5). In the other four patients, only the subdermal administration was further applied. The concentration of the intravenous lidocaine infusion solution is 20 mg/ml (2%), the route for continuous administration of lidocaine commonly used in daily practice. The dose for intravenous administration is 0.6 mg/kg/h as recommended earlier [402]. Due to the limited efficacy of intravenous lidocaine and based on the hypothesis of selectively targeting the inhibition of the P2X7Rs of the immune cells, the infusion in both patients was converted to subdermal infusion of 1.0 mg/kg/h (dosage as reported by Japanese researchers [403]) after 7 and 6 days, respectively. The time course of clinical parameters of these six patients is presented in Figures 7, 8, 9, 10. In about 20% of the inserted subdermal cannulae, local subdermal indurations were observed. Whenever this occurred, the infusion cannula was removed and replaced with a new cannula at a different location.

The first patient (Fig*.*7), a 63-year-old male (75 kg, 168 cm), developed fever and nausea on March 27, 2020, and 3 days later, he started to cough and became dyspnoeic. After 5 days, the PCR SARS-Cov-2 test was positive, and he was admitted to the hospital with SARS-Cov-2-induced ARDS. Co-morbidities include COPD and smoking 60 cigarettes per day for more than 40 years. About 40 years earlier, the patient suffered from pneumothorax. On day 3, the patient deteriorated and was intubated and mechanically ventilated due to poor blood gases. No haemodynamic instability was observed. The CT scan showed bilateral ground glass opacities compatible with ARDS. On day 5, the patient was transferred to the ICU of the university hospital because of further respiratory deterioration. The patient received favipiravir for 14 days after admission; the patient did not receive dexamethasone. Prone position mechanical ventilation was initiated due to the progression of the respiratory disease with an extremely low PaO_2_/FiO_2_ ratio of 63.3 mm Hg (severe ARDS according to the Berlin definition. The Berlin definition of ARDS includes severe PaO_2_/FiO_2_ ratio ≤100 mm Hg, moderate PaO_2_/FiO_2_ >100 to 200 mm Hg, mild PaO2/FiO2 >200 to 300 mm Hg, no ARDS PaO2/FiO2 >300 mm Hg [404]). The initial ventilator settings include APRV, P_high_27 cm H_2_O, T_high_ 7.0 s, P_low_ 0 cm H_2_O and T_low_ 0.32 s. The PaCO_2_ was normal. The echocardiographic estimated pulmonary arterial systolic pressure (PASP) was 80 mm Hg. The Krebs von Lungen 6 (KL-6, a marker for lung fibrosis [405]) plasma level was highly elevated (1299 U/mL; normal value <425 U/mL), CRP was also high (40.4 mg/L; normal value <10 mg/L), and albumin was 2.2 g/dl. The white blood cell count, platelet count and urine production were normal. On day 4, the chest X-ray was not improved. On day 6, the PaO_2_/FiO_2_ ratio was slightly increased but remained low at 103 mm Hg, and the chest X-ray showed progression of the ARDS. ECMO was initiated due to exhausted ventilatory strategy. On day 9, the PaO_2_/FiO_2_ ratio improved but remained low at around 153 mm Hg, but the CRP declined to around 21.8 mg/L. The patient was put on muscle relaxants. The patient’s ARDS status had improved from severe to moderate ARDS. From day 10 until day 30, the ferritin levels were well >1000 ng/ml (>100 μg/dl, normal values <300 ng/ml). From day 11 until day 62, D-Dimer was very high reaching 121.9 nM/L day 14. On day 11, no improvement of the blood gases was observed, and it was decided to treat the patient with continuous intravenous lidocaine 0.6 mg/kg/h. The CRP showed a progressive decline from 19 (on day 12) to 12.8 (on day 16) and 7.4 (on day 19), but the PaO_2_/FiO_2_ ratio remained poor at around 90 mm Hg (severe ARDS according to the Berlin criteria) and the chest X-ray image on day 15, 3 days after the initiation of the intravenous lidocaine infusion, deteriorated dramatically. The lidocaine plasma concentrations were 3.4 μg/ml on day 13 and 5.4 μg/ml on day 14. On day 19, the continuous intravenous lidocaine infusion was replaced by continuous subdermal lidocaine infusion of 1 mg/kg/h. Although the PaO_2_/FiO_2_ ratio remained unchanged on day 20 (1 day after the switch to the continuous subdermal lidocaine), the chest X-ray improved clearly. On day 21, the lidocaine plasma concentration was 2.6 μg/ml, and albumin was 2.5 g/dl. From day 22, the PaO_2_/FiO_2_ratio was gradually improving reaching 151 mm Hg on day 34 (moderate ARDS). The KL-6 on day 22 dropped to 458 U/L (this is only slightly above the normal value of <450 U/l). On day 31, the CRP was low at 1 mg/L, and the lidocaine plasma concentration was 1.2 μg/ml. The muscle relaxants were discontinued. Albumin was 2.3 g/dl. On day 33, the chest X-ray was further improved, and the CRP remained low at 5.5 mg/L. The patient was awake and could communicate with the nurses. On day 38, the lidocaine plasma level was 2.3. On day 43, the PaO_2_/FiO_2_ ratio was increased to 214 mm Hg. According to the Berlin definition of ARDS [404], the patient’s ARDS status had changed from moderate to mild. Albumin was 2.8 g/dl. On day 50, the patient was weaned from ECMO. On day 51, the patient underwent tracheotomy. Because the clinical condition of the patient was stabilised with a low CRP of 6.3 mg/L on day 55, the continuous subdermal lidocaine was discontinued on day 57. On day 69, he developed pneumothorax requiring pleural drainage. On day 99, he was weaned from the mechanical ventilator and was discharged from the ICU on day 121. No new ECG changes were observed during treatment with lidocaine. Blood methaemoglobin (metHb) were within the normal range (0.3–0.8%). CT scan on day 146 showed reduced ground glass opacities in both lungs, some interstitial change in upper and middle fields of the lung and improvement of the pneumothorax. The patient left the hospital on day 187, he went home, and he could walk but needed extra oxygen supply of 2L/min. Nine months after admission, the patient is doing well and has returned to work. The patient visited the hospital 3 months after discharge: He only uses oxygen 1 L/min to go shopping and during physical training (out-patient rehabilitation). He talked to the treating intensivist without requiring oxygen and had no shortness of breath or tachypnoea.

The second patient (Fig*.*8) is a 68-year-old male (75 kg, 164 cm) with SARS-Cov-2-induced ARDS and positive SARS-Cov-2 PCR test admitted to the university hospital. Co-morbidity is asthma. The CT scan showed bilateral ground glass opacities. Haemodynamically the patient was stable. The patient received tocilizumab on day 8 and favipiravir for 14 days; he did not receive dexamethasone. On day 2, the respiratory conditions deteriorated, and the PaO_2_/FiO_2_ ratio is 118 mm Hg (moderate ARDS according to the Berlin ARDS definition [404]). The patient was intubated and required mechanical ventilation. The initial ventilator settings include pressure control, peak inspiratory pressure 28 cm H_2_O, PEEP 13 cm H_2_O and respiratory rate 30/min. CRP was 10.6 mg/L, and KL-6 was 486 U/ml. White blood cell count, platelet count and urine production were normal. The ferritin levels remained >1000 ng/ml (100 μg/dl) during the entire ICU stay. Albumin was 2.9 g/dl. In the following 3 days, the PaO_2_/FiO_2_ ratio improved to around 150 mm Hg. The PaO_2_/FiO_2_ ratio dropped from 152 on day 5 to 84 mm Hg on day 6. CRP was increased to 22.9, and the KL-6 was increased to 762 U/ml. The patient was put in prone position and given muscle relaxants. Continuous intravenous lidocaine of 0.6 ml/kg/h was started. Albumin was 1.8 g/dl. On day 7, the PaO_2_/FiO_2_ ratio increased to 128 mm Hg, CRP dropped to 10.3 mg/mL and the lidocaine plasma concentration was 2.2 μg/ml. From day 3 until discharge from the ICU, D-dimer values were elevated reaching 75 nM/L on day 14. On day 8, although the PaO2/FiO2 ratio improved from 84 to 125 mm Hg, the mechanical ventilatory strategies were exhausted, and the patient was put on ECMO. The KL-6 was increased to 845 U/L, and lidocaine plasma level was 2.9 μg/ml. The PaO_2_/FiO_2_ ratio improved to 238 mm Hg on day 9, but on day 10, a sharp drop of the PaO_2_/FiO_2_ ratio to 60 mm Hg was observed, and CRP was 2.0 mg/ml. The patient’s ARDS status had changed from moderate to severe according to the Berlin ARDS criteria [404]. Lidocaine treatment was switched from continuous intravenous to continuous subdermal (dosage: 1 mg/kg/h). On day 14, the lidocaine plasma level was 2.7 μg/ml. KL-6 dropped to 549 U/l. On day 17, the clinical condition of the patient was improving, and the PaO_2_/FiO_2_ ratio reached 158 mm Hg. The patient was weaned from ECMO. The PaO_2_/FiO_2_ ratio improved further reaching 291 mm Hg on day 21, and the patient’s ARDS status has changed from moderate to mild ARDS [404]. On day 22, mechanical ventilation was discontinued, and the patient was extubated. The patient was orientated, and no signs of confusion were detected. CT scan on day 25 showed persistent ground glass opacities in both lungs, some pulmonary effusion (right >left), and no signs of vascular thrombosis. In addition, no signs of deep venous thrombosis were found in the lower extremities. Lidocaine treatment was continued until discharge from the ICU on day 30. No new ECG changes were observed during treatment with lidocaine. Blood metHb were within the normal range (0.1–0.6%). The patient was discharged home on day 37. At 3 months after admission, the patient is doing well.

The third patient (Fig. 9*left)*, a 59-year-old male (109 kg, 170 cm), was admitted to the university hospital with respiratory distress and bilateral ground glass opacities on the CT scan with a positive SARS-CoV-2 test. Co-morbidities include obesity (BMI 37.7 kg/m^2^), diabetes mellitus and gout. The patient required immediate intubation and mechanical ventilation. The patient received tocilizumab on day 3 and favipiravir for 15 days and did not receive dexamethasone. The initial ventilator settings are pressure control, peak inspiratory pressure 30 cm H_2_O, PEEP 15 cm H_2_O and respiratory rate 25/min. The PaO_2_/FiO_2_ ratio on admission was 160 mm Hg (moderate ARDS according to the Berlin definition [404]), CRP was 39.3 mg/L and KL-6 was 294 U/ml. White blood cell count was increased (13.10^−9^/L) and platelet count and urine production were normal. Albumin was 2.1 g/dl. Haemodynamic parameters were stable. On the admission day, continuous subdermal lidocaine was started at 1 mg/kg/h. On day 2, the PaO_2_/FiO_2_ ratio improved to 283 mm Hg, and the patient’s ARDS status had changed from moderate to mild ARDS. CRP was 41 mg/L, KL-6 was 268 U/L and the lidocaine plasma level was 3.7 μg/ml. Albumin was 1.7 g/dl. On day 4, the PaO_2_/FiO_2_ ratio was 302 mm Hg, and the patient’s ARDS status had changed from mild ARDS to no ARDS according to the Berlin ARDS criteria. On day 5, the PaO_2_/FiO_2_ ratio was improved further to 328 mm Hg, and CRP dropped to 16.4, and the patient was extubated. The patient was orientated, no signs of confusion were detected. The patient was discharged from the ICU on day 8; CRP was 2.3 mg/ml. Albumin was 2.5 g/dl. No new ECG changes were observed during treatment with lidocaine. Blood metHb were within the normal range (0.1–0.4%). The patient was discharged home on day 20. After 3 months, he is doing well.

The fourth patient (Fig. 9*right*) is a 51-year-old male (68 kg, 175 cm). Ten days before admission, he developed fever and 2 days before admission dyspnoea and coughing. On the day of admission, the PCR SARS-CoV-2 test was positive. The CT scan showed bilateral ground glass opacities. Co-morbidity is none. The patient was intubated and put on mechanical ventilation on admission. The patient received favipiravir for 14 days; he did not receive dexamethasone. On day 3, he was transferred to the university hospital because of deterioration of pulmonary condition. The initial ventilator settings include pressure control, peak inspiratory pressure 24 cm H_2_O, PEEP 12 cm H_2_O and respiratory rate 15/min. The haemodynamic conditions were stable. White blood cell count and platelet count were normal. Albumin was 2.6 g/dl. Continuous subdermal lidocaine was started immediately. On day 3, the PaO_2_/FiO_2_ ratio was 214 (moderate ARDS according to the Berlin definition [404]). KL-6 was 177 U/L, and CRP was 17.4 mg/L. On day 5, the PaO_2_/FiO_2_ ratio was increased to 382 (the patient’s ARDS status had changed from mild ARDS to no ARDS), and lidocaine plasma concentration was 5.2 μg/ml. CRP was 27.3mg/L. Lidocaine plasma levels on day 3 and 4 were 3.4 and 4.2 μg/ml, respectively. KL-6 was 163 U/L. The patient was extubated. The patient was orientated, and no signs of confusion were detected. The patient was discharged from the ICU on day 8, and the CRP was 9.3 mg/L. No new ECG changes were observed during treatment with lidocaine. Blood metHb were within the normal range (0.1–0.3%). He was discharged home on day 28. At 3 months, he is doing well and has returned to work.

The fifth patient (Fig. 10*left*) is a 58-year-old male (80 kg, 175 cm). Nine days before admission, he developed a sore throat. A day later, he developed fever. Two days before admission, he started coughing and was dyspnoeic. On the day of admission, the PCR SARS-Cov-2 test was positive. Co-morbidity includes fatty liver. The CT scan showed bilateral ground glass opacities. The patient was initially admitted to the hospital ward. The patient received tocilizumab on day 7 and favipiravir for 10 days; dexamethasone was not prescribed. On day 3, the patient deteriorated and had to be intubated and put on mechanical ventilation. On day 4, the patient was transferred to the university hospital due to deterioration of the pulmonary condition. The initial ventilator settings include pressure control, peak inspiratory pressure 27 cm H2O, PEEP 12 cm H2O and respiratory rate 25/min. PaO_2_/FiO_2_ ratio was 188 (moderate ARDS according to the Berlin definition). Haemodynamic parameters were stable, and CRP was 12.9 mg/ml. White blood cell count was increased (14.4.10^9^/L), but platelet count was normal. KL-6 was 330 U/L. Continuous subdermal lidocaine was started at 1 mg/kg/h at arrival at the ICU of the university hospital. Albumin was 2.8 g/dl. On day 5, the PaO_2_/FiO_2_ ratio was unchanged, CRP was 10.4 mg/L and the lidocaine plasma level was 4 μg/ml. On day 6, the lidocaine plasma level was 3.2 μg/ml. KL-6 remained stable at 400 U/L. Albumin was 2.3 g/dl. On day 10, the respiratory insufficiency had cleared; although the PaO2/FiO2 ratio remained 184, the CRP dropped to 2.4 mg/L, and KL-6 was 322 U/L. The patient was extubated, and he was orientated; no signs of confusion were detected. On day 14, the patient was discharged from the ICU. No new ECG changes were observed during treatment with lidocaine. Blood metHb were within the normal range (0.1–0.3%). On day 20, the patient was discharged home and is doing well at 3 months after admission.

The sixth patient (Fig. 10*right*) is a 59-year-old male (65 kg, 175 cm) with fever, dyspnoea and cough due to COVID-19. CT scan showed bilateral ground glass opacities. Co-morbidity includes hypertension on medication. The patient was admitted to the general ward. KL-6 233 U/L, white blood cell count and platelet count were normal. Albumin was 3.6 g/dl. On day 3, there is a deterioration of the respiratory function necessitating a transfer to the ICU and mechanical ventilation. Tocilizumab was given on day 4. The patient received favipiravir for 11 days, and the patient did not receive dexamethasone. The initial ventilator settings include pressure control, peak inspiratory pressure 22 cm H_2_O, PEEP 10 cm H_2_O and respiratory rate 20/min. Continuous subdermal lidocaine of 1 mg/kg/h was initiated after admission to the ICU. Haemodynamic parameters were stable. CRP was 6.3 mg/L, and KL-6 was 263 U/L. On day 4, a progressive respiratory failure occurred requiring intubation and mechanical ventilation. PaO_2_/FiO_2_ ratio was 218 mm Hg; the haemodynamic parameters remained stable. CRP was 6.3 mg/L, and the white blood count and platelet count were normal. Lidocaine plasma level was 4.6 μg/ml. On day 5, the PaO_2_/FiO_2_ ratio dropped further to 164 mm Hg. Lidocaine plasma level was 3.4 μg/ml. Albumin was 3.2 g/dl. On day 9, the clinical condition of the patient improved. The ventilator settings could be decreased, the PaO_2_/FiO_2_ ratio remained 207 mm Hg during the weaning period, and CRP was 0.7 mg/L. On day 10, the patient was extubated, he was orientated, and no signs of confusion were detected. On day 13, the patient was discharged from the ICU. No new ECG changes were observed during treatment with lidocaine. Blood metHb were within the normal range (0.1–0.3%). He was discharged from the hospital on day 20, and at 3 months after admission, he is doing well, played golf regularly and has returned to work.

### Additional 14 patients with COVID-19-induced ARDS

From July 2020 until beginning of December 2020, 14 additional critically ill patients with COVID-19-induced ARDS requiring mechanical ventilation were treated in the ICU of the Showa University with continuous subdermal lidocaine infusion (1 mg/kg/h) plus intravenous or oral dexamethasone (6 mg/day) as reported earlier [35]. Of these 20 patients, 19 survived, but an 87-year-old female patient died of invasive aspergillosis. No other patient developed secondary co-infections (unpublished data, personal communication by AS and TK).

### Discussion of the clinical cases

After completing the novel definition of hyperinflammation, we developed a new approach to target the lymphatic system with continuous subdermal administration of lidocaine. This is meant to increase the anti-hyperinflammatory effect of lidocaine while avoiding toxic plasma levels. We described the treatment of six critically ill patients with COVID-19 with lidocaine. Two patients required mechanical ventilation and ECMO, and four patients were treated with mechanical ventilation. As mentioned under the heading “Introduction”, the case fatality rates of patients requiring mechanical ventilation and/or ECMO are alarmingly high [1, 2]. Patient 1 and patient 2 were older than 60 years. Additionally, patient 1 had COPD and had smoked 60 cigarettes per day for more than 40 years. Patient 3 suffered from obesity and diabetes mellitus. These are serious prognostic factors for bad outcome COVID-19 [406, 407]. Patient 1 and patient 2 were initially treated with continuous intravenous lidocaine through a central venous line. In both patients, the pulmonary conditions deteriorated after the initiation of intravenous lidocaine: Patient 1 who was already on ECMO showed progressive pulmonary deterioration on the chest X-rays, and patient 2 deteriorated further necessitating the initiation of ECMO therapy. Remarkably, the pulmonary conditions of both patients improved within 48 h after the switch from intravenous to subdermal continuous lidocaine. The lidocaine plasma levels remained around 5 μg/ml. To our knowledge, these six cases represent the first observations of the promising treatment of critically ill COVID-19 patients with lidocaine targeting P2X7Rs of the immune cells in the lymphatics. All patients recovered completely from their illness. None of the patients showed the feared side effect of cardiac arrhythmia and methaemoglobinaemia during lidocaine therapy. Our findings suggest that continuous subdermal lidocaine infusion at the rate of 1 mg/kg/h has the potential to mitigate hyperinflammation and ARDS in critically ill COVID-19-patients. Obviously, although all six patients appeared to respond positively to the treatment and no severe adverse effects were observed, no final conclusions can be made on the efficacy of lidocaine in critically ill COVID-19 patients.

Researchers from Lima, Peru, reported the treatment of 28 (three mild, 21 moderate and four severe) COVID-19 patients with 0.5% lidocaine HCL solution with an intravenous dose of 1 mg/kg once a day for 2 days and 2% lidocaine HCL solution with a subcutaneous dose of 1 mg/kg once a day for 2 days [408]. The authors aimed at the improvement of pain, cough, respiratory rate and oxygen saturation. They found improvement in most patients. In severe cases, this treatment did not improve the oxygen saturation. As expected, treatment with a low daily dose of lidocaine once per day for a total treatment duration of 2 days could not adequately inhibit the P2X7R-induced hyperinflammation in COVID-19.

Recently, a group of researchers from Strasbourg, France, announced a study entitled: “Impact of intravenous lidocaine on clinical outcomes of patients with ARDS during COVID-19 pandemia (LidoCovid): A structured summary of a study protocol for a randomised controlled trial” (ClinicalTrials.gov Identifier: NCT04609865) [409].

Lately, an extraordinary treatment of COVID-19 ARDS was reported [410]. The authors performed lung transplantations in three critically ill COVID-19 ARDS patients: a 28-year-old female, a 62-year-old male and a 43-year-old male. The first patient underwent lung transplantation after weeks on veno-venous ECMO support with elevated pulmonary arterial pressures and severe secondary Serratia marcescens pneumonia. The second patient underwent lung transplantation after 100 days on veno-venous ECMO support complicated by Pseudomonas aeruginosa pneumonia, haemothorax and empyema, while the third patient after 90 days on the mechanical ventilator. This patient suffered from many complications: asystolic cardiac arrest, heparin-induced thrombocytopenia, a left frontal lobe infarct of the cerebral cortex, Serratia marcescens-mediated pneumonia with bacteraemia, acute kidney injury, a left haemothorax requiring thoracotomy and lung decortication, a right pneumothorax requiring tube thoracostomy, hypernatremia associated with seizures and malnutrition. Before lung transplantation, the patient developed increasing clinical signs of pulmonary fibrosis and severe pulmonary hypertension. The first two patients are reported to have achieved independence in daily life activities several months after lung transplantation. Three months after lung transplantation, the third patient made improvements in the neurocognitive status and muscular strength at an inpatient rehabilitation centre.

Far less drastic is our proposed treatment of hyperinflammation in COVID-19-induced ARDS with lidocaine, an old drug that is readily available to hospitals all over the world at a low cost. In November 1948, Xylocaine was approved by the Food and Drug Administration (FDA) in the USA [411]. Lidocaine is used as a local anaesthetic [411], treatment of chronic neuropathic pain [412] but also for the prophylaxis or treatment of ventricular arrhythmia [328, 329]. Recently, intravenous lidocaine has been administered as general anaesthetic replacing opioids in the perioperative settings [413]. Potentially, lidocaine, as a P2X7R antagonist, can abrogate hyperinflammation, can restore the capacity of the immune system to combat secondary co-infections and can improve the clinical condition in critically ill COVID-19 patients. Despite several in vitro [326, 327, 414, 415], animal studies [319, 416–420] and patient cohorts [408, 421] on the anti-inflammatory properties of lidocaine, completed clinical trials which deliver a proof of concept (i.e. a randomised controlled trial) have not yet been performed. We postulate that because the maximal tolerable plasma concentration of lidocaine is much lower than the required extracellular concentration to effectively inhibit P2X7Rs, intravenous systemic administration of lidocaine simply cannot not be used to effectively treat hyperinflammation. This is a plausible reason why 5 years after the discovery of lidocaine as a P2X7R inhibitor (published in 2015) [74] the drug is still not used as an anti-hyperinflammatory treatment in clinical practice.

## Concluding remarks

As stated in the introduction, therapeutic measures that can immediately attenuate the course of SARS-CoV-2-related lung damage are promptly needed on a global scale. In contrast to the investigational P2X7R antagonists described above, continuous subdermal infusion of 2% lidocaine solution to primarily deposit lidocaine into the lymphatics is readily available and can be used in the daily practice immediately and, in principle, even outside the ICU and is very well affordable. Therefore, this therapy deserves to be investigated in larger placebo controlled randomised clinical studies with COVID-19 patients.

## Future development

However, our experience with subdermal administration of lidocaine in the ICU made clear that this method may not be routinely suitable outside hospital settings. Needless to say that high complexity and high-cost treatments (requiring highly skilled nurses and infusion pump equipment) are inaccessible to low-income COVID-19 patients in developing countries. Also, as the severity and case fatality rate of COVID-19 increase with age [406], the case fatality rate in elderly patients in nursing homes is strikingly high, and many residents have poor access to medical care [422]. This encouraged us to explore alternative uncomplicated methods of lidocaine administration accessible to everyone, particularly elderly COVID-19 patients and COVID-19 patients in developing countries.

Recently, researchers stated in their article on targeting the P2X7R in COVID-19 that the P2X7R antagonists for human use are available only in oral form and that this might be an inefficient route of drug delivery [298]. We found a solution to this problem. Permeability of the skin and mucous membrane to water, drugs, etc. is said to be dependent on the site of the administration [423, 424]. For example, the permeability constant of the floor of the mouth (sublingual mucosa), lateral border of the tongue and buccal mucosa for tritium-labelled water is 22, 17 and 13 times as high as human skin, respectively [423]. We argue that this also applies to lidocaine. As mentioned above, the endothelium of the mucosal capillaries of the mouth and the gastrointestinal tract belong to the structural type “fenestrated endothelium” allowing molecules to pass from the submucosal tissue into the capillaries [383]. Lidocaine hydrochloride is highly soluble in water (solubility of 680 mg/ml in water) [425] and therefore will mainly be absorbed by the submucosal capillary [426] and the inductive sites of MALT [398, 399]. In contrast, the highly lipophilic lidocaine base (solubility of 4 mg/ml in water, 760 mg/ml in 95% ethanol and 790 mg/ml in chloroform) [425] is preferably absorbed by the local initial lymphatics in the submucosal tissue [426, 427]. In addition, the lymphatic drainage of the floor of the mouth is extensive, involving many lymph nodes [428–431].

We estimate that with a sublingual administration of lipophilic lidocaine base (Fig. 1), we may reach the IC_50_ of the P2X7Rs in the draining lymph nodes to control systemic hyperinflammation and avoid toxic lidocaine plasma levels (Figs. 4 and 6). Obviously, such solution may also apply to other P2X7R antagonists. We stress that sublingual and buccal administration of lipophilic lidocaine is different from oral administration of lidocaine. Oral administration of lidocaine is aimed at the resorption of the drug in the gastrointestinal tract (Fig*.*6).

There are other methods of targeting the immune cells in the lymphatics, i.e. transdermal administration of lipophilic P2X7R antagonist with skin penetration enhancers (i.e. alpha-terpineol [432], ethanol [433] and lipid based nanoformulations [434]), intravenous administration of a P2X7R antagonist using nano-sized drug delivery systems [435], liposomes or polymer micelles [436] and oral administration of a P2X7R antagonist using delivery systems for intestinal lymphatic drug transport such as chylomicrons [437].

## Data Availability

The datasets generated during and/or analysed during the current study are not publicly available due to privacy reasons but are available from the corresponding author on reasonable request.

## References

[1] Quah P, Li A, Phua J (2020). Mortality rates of patients with COVID-19 in the intensive care unit: a systematic review of the emerging literature. Crit Care.

[2] Barbaro RP, MacLaren G, Boonstra PS, Iwashyna TJ, Slutsky AS, Fan E, Bartlett RH, Tonna JE, Hyslop R, Fanning JJ, Rycus PT, Hyer SJ, Anders MM, Agerstrand CL, Hryniewicz K, Diaz R, Lorusso R, Combes A, Brodie D (2020). Extracorporeal membrane oxygenation support in COVID-19: an international cohort study of the Extracorporeal Life Support Organization registry. Lancet.

[3] Manson JJ, Crooks C, Naja M, Ledlie A, Goulden B, Liddle T, Khan E, Mehta P, Martin-Gutierrez L, Waddington KE, Robinson GA, Ribeiro Santos L, McLoughlin E, Snell A, Adeney C, Schim van der Loeff I, Baker KF, Duncan CJA, Hanrath AT, Lendrem BC, De Soyza A, Peng J, J'Bari H, Greenwood M, Hawkins E, Peckham H, Marks M, Rampling T, Luintel A, Williams B, Brown M, Singer M, West J, Jury EC, Collin M, Tattersall RS (2020). COVID-19-associated hyperinflammation and escalation of patient care: a retrospective longitudinal cohort study. Lancet Rheumatol.

[4] Gustine JN, Jones D (2021). Immunopathology of hyperinflammation in COVID-19. Am J Pathol.

[5] Afrin LB, Weinstock LB, Molderings GJ (2020). Covid-19 hyperinflammation and post-Covid-19 illness may be rooted in mast cell activation syndrome. Int J Infect Dis.

[6] Fajgenbaum DC, June CH (2020). Cytokine Storm. N Engl J Med.

[7] Webb BJ, Peltan ID, Jensen P, Hoda D, Hunter B, Silver A, Starr N, Buckel W, Grisel N, Hummel E, Snow G, Morris D, Stenehjem E, Srivastava R, Brown SM (2020). Clinical criteria for COVID-19-associated hyperinflammatory syndrome: a cohort study. Lancet Rheumatol.

[8] Cardone M, Yano M, Rosenberg AS, Puig M (2020). Lessons learned to date on COVID-19 hyperinflammatory syndrome: considerations for interventions to mitigate SARS-CoV-2 viral infection and detrimental hyperinflammation. Front Immunol.

[9] Bozzi G, Mangioni D, Minoia F, Aliberti S, Grasselli G, Barbetta L, Castelli V, Palomba E, Alagna L, Lombardi A, Ungaro R, Agostoni C, Baldini M, Blasi F, Cesari M, Costantino G, Fracanzani AL, Montano N, Monzani V, Pesenti A, Peyvandi F, Sottocorno M, Muscatello A, Filocamo G, Gori A, Bandera A (2021). Anakinra combined with methylprednisolone in patients with severe COVID-19 pneumonia and hyperinflammation: an observational cohort study. J Allergy Clin Immunol.

[10] Landewé RBM, Ramiro S, Mostard RLM (2021)COVID-19-induced hyperinflammation, immunosuppression, recovery and survival: how causal inference may help draw robust conclusions. RMD Open 7(1). 10.1136/rmdopen-2021-001638 10.1136/rmdopen-2021-001638 PMC8015793 33790049

[11] Anka AU, Tahir MI, Abubakar SD, Alsabbagh M, Zian Z, Hamedifar H, Sabzevari A, Azizi G (2021). Coronavirus disease 2019 (COVID-19): an overview of the immunopathology, serological diagnosis and management. Scand J Immunol.

[12] Mehta P, McAuley DF, Brown M, Sanchez E, Tattersall RS, Manson JJ (2020) COVID-19: consider cytokine storm syndromes and immunosuppression. Lancet. 10.1016/s0140-6736(20)30628-0 10.1016/S0140-6736(20)30628-0 PMC7270045 32192578

[13] Freeman TL, Swartz TH (2020). Targeting the NLRP3 Inflammasome in severe COVID-19. Front Immunol.

[14] De Luca G, Cavalli G, Campochiaro C, Della-Torre E, Angelillo P, Tomelleri A, Boffini N, Tentori S, Mette F, Farina N, Rovere-Querini P, Ruggeri A, D'Aliberti T, Scarpellini P, Landoni G, De Cobelli F, Paolini JF, Zangrillo A, Tresoldi M, Trapnell BC, Ciceri F, Dagna L (2020). GM-CSF blockade with mavrilimumab in severe COVID-19 pneumonia and systemic hyperinflammation: a single-centre, prospective cohort study. Lancet Rheumatol.

[15] Wang L, He W, Yu X, Hu D, Bao M, Liu H, Zhou J, Jiang H (2020) Coronavirus disease 2019 in elderly patients: Characteristics and prognostic factors based on 4-week follow-up. J Inf Secur. 10.1016/j.jinf.2020.03.019 10.1016/j.jinf.2020.03.019 PMC7118526 32240670

[16] Lv Z, Cheng S, Le J, Huang J, Feng L, Zhang B, Li Y (2020). Clinical characteristics and co-infections of 354 hospitalized patients with COVID-19 in Wuhan, China: a retrospective cohort study. Microbes Infect.

[17] Kooistra EJ, Waalders NJB, Grondman I, Janssen NAF, de Nooijer AH, Netea MG, van de Veerdonk FL, Ewalds E, van der Hoeven JG, Kox M, Pickkers P (2020). Anakinra treatment in critically ill COVID-19 patients: a prospective cohort study. Crit Care.

[18] Xu X, Han M, Li T, Sun W, Wang D, Fu B, Zhou Y, Zheng X, Yang Y, Li X, Zhang X, Pan A, Wei H (2020). Effective treatment of severe COVID-19 patients with tocilizumab. Proc Natl Acad Sci U S A.

[19] Perrone F, Piccirillo MC, Ascierto PA, Salvarani C, Parrella R, Marata AM, Popoli P, Ferraris L, Marrocco-Trischitta MM, Ripamonti D, Binda F, Bonfanti P, Squillace N, Castelli F, Muiesan ML, Lichtner M, Calzetti C, Salerno ND, Atripaldi L, Cascella M, Costantini M, Dolci G, Facciolongo NC, Fraganza F, Massari M, Montesarchio V, Mussini C, Negri EA, Botti G, Cardone C, Gargiulo P, Gravina A, Schettino C, Arenare L, Chiodini P, Gallo C (2020). Tocilizumab for patients with COVID-19 pneumonia. The single-arm TOCIVID-19 prospective trial. J Transl Med.

[20] Luis BM, Miguel MB, Pedro DL, David IP, Itziar A, Ana GH, Enrique IJ, María LV, Noelia TF, Julio César BB, Marta UI, Rodrigo SL, María CB, Andrés LM, Javier MI, Juan Pablo GM, Gerardo HF, Carolina NF, Jorge BL, María FR, Fernando CT, Sergio OE, Lourdes FC, María GE, Gregoria ML, Adolfo SR, José Antonio FR (2021). Benefits of early aggressive immunomodulatory therapy (tocilizumab and methylprednisolone) in COVID-19: single center cohort study of 685 patients. J Transl Autoimmun.

[21] Gokhale Y, Mehta R, Kulkarni U, Karnik N, Gokhale S, Sundar U, Chavan S, Kor A, Thakur S, Trivedi T, Kumar N, Baveja S, Wadal A, Kolte S, Deolankar A, Pednekar S, Kalekar L, Padiyar R, Londhe C, Darole P, Pol S, Gokhe SB, Padwal N, Pandey D, Yadav D, Joshi A, Badgujar H, Trivedi M, Shah P, Bhavsar P (2021). Tocilizumab improves survival in severe COVID-19 pneumonia with persistent hypoxia: a retrospective cohort study with follow-up from Mumbai, India. BMC Infect Dis.

[22] Aomar-Millán IF, Salvatierra J, Torres-Parejo Ú, Faro-Miguez N, Callejas-Rubio JL, Ceballos-Torres Á, Cruces-Moreno MT, Gómez-Jiménez FJ, Hernández-Quero J, Anguita-Santos F (2021). Anakinra after treatment with corticosteroids alone or with tocilizumab in patients with severe COVID-19 pneumonia and moderate hyperinflammation. A retrospective cohort study. Intern Emerg Med.

[23] Cavalli G, Larcher A, Tomelleri A, Campochiaro C, Della-Torre E, De Luca G, Farina N, Boffini N, Ruggeri A, Poli A, Scarpellini P, Rovere-Querini P, Tresoldi M, Salonia A, Montorsi F, Landoni G, Castagna A, Ciceri F, Zangrillo A, Dagna L (2021). Interleukin-1 and interleukin-6 inhibition compared with standard management in patients with COVID-19 and hyperinflammation: a cohort study. Lancet Rheumatol.

[24] Gordon AC, Mouncey PR, Al-Beidh F, Rowan KM, Nichol AD, Arabi YM, Annane D, Beane A, van Bentum-Puijk W, Berry LR, Bhimani Z, Bonten MJM, Bradbury CA, Brunkhorst FM, Buzgau A, Cheng AC, Detry MA, Duffy EJ, Estcourt LJ, Fitzgerald M, Goossens H, Haniffa R, Higgins AM, Hills TE, Horvat CM, Lamontagne F, Lawler PR, Leavis HL, Linstrum KM, Litton E, Lorenzi E, Marshall JC, Mayr FB, McAuley DF, McGlothlin A, McGuinness SP, McVerry BJ, Montgomery SK, Morpeth SC, Murthy S, Orr K, Parke RL, Parker JC, Patanwala AE, Pettilä V, Rademaker E, Santos MS, Saunders CT, Seymour CW, Shankar-Hari M, Sligl WI, Turgeon AF, Turner AM, van de Veerdonk FL, Zarychanski R, Green C, Lewis RJ, Angus DC, McArthur CJ, Berry S, Webb SA, Derde LPG (2021). Interleukin-6 receptor antagonists in critically ill patients with Covid-19. N Engl J Med.

[25] The CORIMUNO-19 Collaborative group (2021). Effect of anakinra versus usual care in adults in hospital with COVID-19 and mild-to-moderate pneumonia (CORIMUNO-ANA-1): a randomised controlled trial. Lancet Respir Med.

[26] Hermine O, Mariette X, Tharaux PL, Resche-Rigon M, Porcher R, Ravaud P (2021). Effect of tocilizumab vs usual care in adults hospitalized with COVID-19 and moderate or severe pneumonia: a randomized clinical trial. JAMA Intern Med.

[27] Ramiro S, Mostard RLM, Magro-Checa C, van Dongen CMP, Dormans T, Buijs J, Gronenschild M, de Kruif MD, van Haren EHJ, van Kraaij T, Leers MPG, Peeters R, Wong DR, Landewé RBM (2020). Historically controlled comparison of glucocorticoids with or without tocilizumab versus supportive care only in patients with COVID-19-associated cytokine storm syndrome: results of the CHIC study. Ann Rheum Dis.

[28] Rosas IO, Bräu N, Waters M, Go RC, Hunter BD, Bhagani S, Skiest D, Aziz MS, Cooper N, Douglas IS, Savic S, Youngstein T, Del Sorbo L, Cubillo Gracian A, De La Zerda DJ, Ustianowski A, Bao M, Dimonaco S, Graham E, Matharu B, Spotswood H, Tsai L, Malhotra A (2021). Tocilizumab in hospitalized patients with severe Covid-19 pneumonia. N Engl J Med.

[29] Salama C, Han J, Yau L, Reiss WG, Kramer B, Neidhart JD, Criner GJ, Kaplan-Lewis E, Baden R, Pandit L, Cameron ML, Garcia-Diaz J, Chávez V, Mekebeb-Reuter M, Lima de Menezes F, Shah R, González-Lara MF, Assman B, Freedman J, Mohan SV (2021). Tocilizumab in patients hospitalized with Covid-19 pneumonia. N Engl J Med.

[30] Salvarani C, Dolci G, Massari M, Merlo DF, Cavuto S, Savoldi L, Bruzzi P, Boni F, Braglia L, Turrà C, Ballerini PF, Sciascia R, Zammarchi L, Para O, Scotton PG, Inojosa WO, Ravagnani V, Salerno ND, Sainaghi PP, Brignone A, Codeluppi M, Teopompi E, Milesi M, Bertomoro P, Claudio N, Salio M, Falcone M, Cenderello G, Donghi L, Del Bono V, Colombelli PL, Angheben A, Passaro A, Secondo G, Pascale R, Piazza I, Facciolongo N, Costantini M (2021). Effect of tocilizumab vs standard care on clinical worsening in patients hospitalized with COVID-19 pneumonia: a randomized clinical trial. JAMA Intern Med.

[31] Soin AS, Kumar K, Choudhary NS, Sharma P, Mehta Y, Kataria S, Govil D, Deswal V, Chaudhry D, Singh PK, Gupta A, Agarwal V, Kumar S, Sangle SA, Chawla R, Narreddy S, Pandit R, Mishra V, Goel M, Ramanan AV (2021) Tocilizumab plus standard care versus standard care in patients in India with moderate to severe COVID-19-associated cytokine release syndrome (COVINTOC): an open-label, multicentre, randomised, controlled, phase 3 trial. Lancet Respir Med. 10.1016/s2213-2600(21)00081-3 10.1016/S2213-2600(21)00081-3 PMC8078880 33676589

[32] Stone JH, Frigault MJ, Serling-Boyd NJ, Fernandes AD, Harvey L, Foulkes AS, Horick NK, Healy BC, Shah R, Bensaci AM, Woolley AE, Nikiforow S, Lin N, Sagar M, Schrager H, Huckins DS, Axelrod M, Pincus MD, Fleisher J et al (2020) Efficacy of tocilizumab in patients hospitalized with Covid-19. N Engl J Med. 10.1056/NEJMoa2028836 10.1056/NEJMoa2028836 PMC7646626 33085857

[33] Huang E, Jordan SC (2020). Tocilizumab for Covid-19 - the ongoing search for effective therapies. N Engl J Med.

[34] Beigel JH, Tomashek KM, Dodd LE, Mehta AK, Zingman BS, Kalil AC, Hohmann E, Chu HY, Luetkemeyer A, Kline S, Lopez de Castilla D, Finberg RW, Dierberg K, Tapson V, Hsieh L, Patterson TF, Paredes R, Sweeney DA, Short WR, Touloumi G, Lye DC, Ohmagari N, Oh MD, Ruiz-Palacios GM, Benfield T, Fätkenheuer G, Kortepeter MG, Atmar RL, Creech CB, Lundgren J, Babiker AG, Pett S, Neaton JD, Burgess TH, Bonnett T, Green M, Makowski M, Osinusi A, Nayak S, Lane HC (2020). Remdesivir for the treatment of Covid-19 - final report. N Engl J Med.

[35] Horby P, Lim WS, Emberson JR, Mafham M, Bell JL, Linsell L, Staplin N, Brightling C, Ustianowski A, Elmahi E, Prudon B, Green C, Felton T, Chadwick D, Rege K, Fegan C, Chappell LC, Faust SN, Jaki T, Jeffery K, Montgomery A, Rowan K, Juszczak E, Baillie JK, Haynes R, Landray MJ (2021). Dexamethasone in hospitalized patients with Covid-19. N Engl J Med.

[36] Kim PS, Read SW, Fauci AS (2020). Therapy for early COVID-19: a critical need. JAMA.

[37] Cain DW, Cidlowski JA (2017). Immune regulation by glucocorticoids. Nat Rev Immunol.

[38] Tang X, Feng YM, Ni JX, Zhang JY, Liu LM, Hu K, Wu XZ, Zhang JX, Chen JW, Zhang JC, Su J, Li YL, Zhao Y, Xie J, Ding Z, He XL, Wang W, Jin RH, Shi HZ, Sun B (2021). Early use of corticosteroid may prolong SARS-CoV-2 shedding in non-intensive care unit patients with COVID-19 pneumonia: a multicenter, single-blind, randomized control trial. Respiration.

[39] Sanders JM, Monogue ML, Jodlowski TZ, Cutrell JB (2020). Pharmacologic treatments for coronavirus disease 2019 (COVID-19): a review. JAMA.

[40] World Health Organisation (2021). COVID-19 vaccines: resolving deployment challenges. Bull World Health Organ.

[41] Troiano G, Nardi A (2021). Vaccine hesitancy in the era of COVID-19. Public Health.

[42] Paris C, Bénézit F, Geslin M, Polard E, Baldeyrou M, Turmel V, Tadié É, Garlantezec R, Tattevin P (2021)COVID-19 vaccine hesitancy among healthcare workers. Infect Dis Now. 10.1016/j.idnow.2021.04.001 10.1016/j.idnow.2021.04.001 PMC8098031 33964486

[43] Dzieciolowska S, Hamel D, Gadio S, Dionne M, Gagnon D, Robitaille L, Cook E, Caron I, Talib A, Parkes L, Dubé È, Longtin Y (2021)Covid-19 vaccine acceptance, hesitancy, and refusal among Canadian healthcare workers: a multicenter survey. Am J Infect Control. 10.1016/j.ajic.2021.04.079 10.1016/j.ajic.2021.04.079 PMC8079260 33930516

[44] Phillips N (2021). The coronavirus is here to stay - here's what that means. Nature.

[45] Drury AN, Szent-Gyorgyi A (1929). The physiological activity of adenine compounds with especial reference to their action upon the mammalian heart. J Physiol.

[46] Lohmann K (1929). Über die Pyrophosphatfraktion im Muskel. Naturwissenschaften.

[47] Engelhardt WA, Ljubimowa MN (1939). Myosine and adenosinetriphosphatase. Nature.

[48] Lipmann F (1940). A phosphorylated oxygenation product of pyruvic acid. J Biol Chem.

[49] Lipmann F (1944). Enzymatic synthesis of acetyl phosphate. J Biol Chem.

[50] Lipmann F, Jones ME, Black S, Flynn RM (1953). The mechanism of the ATP-CoA-acetate reaction. J Cell Physiol Suppl.

[51] Feldberg W, Hebb C (1948). The stimulating action of phosphate compounds on the perfused superior cervical ganglion of the cat. J Physiol.

[52] Holton P (1959). The liberation of adenosine triphosphate on antidromic stimulation of sensory nerves. J Physiol.

[53] Burnstock G (1972). Purinergic nerves. Pharmacol Rev.

[54] Burnstock G (2012). Purinergic signalling: its unpopular beginning, its acceptance and its exciting future. Bioessays.

[55] Burnstock G (2014). Purinergic signalling: from discovery to current developments. ExpPhysiol.

[56] Di Virgilio F (1995). The P2Z purinoceptor: an intriguing role in immunity, inflammation and cell death. Immunol Today.

[57] Eltzschig HK, Sitkovsky MV, Robson SC (2012). Purinergic signaling during inflammation. N Engl J Med.

[58] Burnstock G (2007). Physiology and pathophysiology of purinergic neurotransmission. Physiol Rev.

[59] Patel AS, Reigada D, Mitchell CH, Bates SR, Margulies SS, Koval M (2005). Paracrine stimulation of surfactant secretion by extracellular ATP in response to mechanical deformation. Am J Physiol Lung Cell MolPhysiol.

[60] Hasan D, Blankman P, Nieman GF (2017). Purinergic signalling links mechanical breath profile and alveolar mechanics with the pro-inflammatory innate immune response causing ventilation-induced lung injury. Purinergic Signal.

[61] Hasan D, Satalin J, van der Zee P, Kollisch-Singule M, Blankman P, Shono A, Somhorst P, den Uil C, Meeder H, Kotani T, Nieman GF (2018) Excessive extracellular ATP desensitizes P2Y2 and P2X4 ATP receptors provoking surfactant impairment ending in ventilation-induced lung injury. Int J Mol Sci 19(4). 10.3390/ijms19041185 10.3390/ijms19041185 PMC5979391 29652806

[62] Takahara N, Ito S, Furuya K, Naruse K, Aso H, Kondo M, Sokabe M, Hasegawa Y (2014). Real-time imaging of ATP release induced by mechanical stretch in human airway smooth muscle cells. Am J Respir Cell Mol Biol.

[63] Furuya K, Tan JJ, Boudreault F, Sokabe M, Berthiaume Y, Grygorczyk R (2016)Real-time imaging of inflation-induced ATP release in the ex-vivo rat lung. Am J Phys Lung Cell Mol Phys. 10.1152/ajplung.00425.2015 10.1152/ajplung.00425.2015 27638905

[64] Bodin P, Burnstock G (1995). Synergistic effect of acute hypoxia on flow-induced release of ATP from cultured endothelial cells. Experientia.

[65] Seminario-Vidal L, Kreda S, Jones L, O'Neal W, Trejo J, Boucher RC, Lazarowski ER (2009). Thrombin promotes release of ATP from lung epithelial cells through coordinated activation of rho- and Ca2+-dependent signaling pathways. J Biol Chem.

[66] Guzman-Aranguez A, Perez de Lara MJ, Pintor J (2017). Hyperosmotic stress induces ATP release and changes in P2X7 receptor levels in human corneal and conjunctival epithelial cells. Purinergic Signal.

[67] Nandigama R, Padmasekar M, Wartenberg M, Sauer H (2006). Feed forward cycle of hypotonic stress-induced ATP release, purinergic receptor activation, and growth stimulation of prostate cancer cells. J Biol Chem.

[68] Cheung-Flynn J, Alvis BD, Hocking KM, Guth CM, Luo W, McCallister R, Chadalavada K, Polcz M, Komalavilas P, Brophy CM (2019). Normal saline solutions cause endothelial dysfunction through loss of membrane integrity, ATP release, and inflammatory responses mediated by P2X7R/p38 MAPK/MK2 signaling pathways. PLoS One.

[69] Bodin P, Burnstock G (1998). Increased release of ATP from endothelial cells during acute inflammation. Inflamm Res.

[70] Okada SF, Ribeiro CM, Sesma JI, Seminario-Vidal L, Abdullah LH, van Heusden C, Lazarowski ER, Boucher RC (2013). Inflammation promotes airway epithelial ATP release via calcium-dependent vesicular pathways. Am J Respir Cell Mol Biol.

[71] Kim KC, Zheng QX, Van-Seuningen I (1993). Involvement of a signal transduction mechanism in ATP-induced mucin release from cultured airway goblet cells. Am J Respir Cell Mol Biol.

[72] Tozzi M, Larsen AT, Lange SC, Giannuzzo A, Andersen MN, Novak I (2018). The P2X7 receptor and pannexin-1 are involved in glucose-induced autocrine regulation in beta-cells. Sci Rep.

[73] Jacques-Silva MC, Correa-Medina M, Cabrera O, Rodriguez-Diaz R, Makeeva N, Fachado A, Diez J, Berman DM, Kenyon NS, Ricordi C, Pileggi A, Molano RD, Berggren PO, Caicedo A (2010). ATP-gated P2X3 receptors constitute a positive autocrine signal for insulin release in the human pancreatic beta cell. Proc Natl Acad Sci U S A.

[74] Okura D, Horishita T, Ueno S, Yanagihara N, Sudo Y, Uezono Y, Minami T, Kawasaki T, Sata T (2015). Lidocaine preferentially inhibits the function of purinergic P2X7 receptors expressed in Xenopus oocytes. Anesth Analg.

[75] Adinolfi E, Callegari MG, Ferrari D, Bolognesi C, Minelli M, Wieckowski MR, Pinton P, Rizzuto R, Di Virgilio F (2005). Basal activation of the P2X7 ATP receptor elevates mitochondrial calcium and potential, increases cellular ATP levels, and promotes serum-independent growth. Mol Biol Cell.

[76] Ghazi K, Deng-Pichon U, Warnet JM, Rat P (2012). Hyaluronan fragments improve wound healing on in vitro cutaneous model through P2X7 purinoreceptor basal activation: role of molecular weight. PLoS One.

[77] Adinolfi E, Callegari MG, Cirillo M, Pinton P, Giorgi C, Cavagna D, Rizzuto R, Di Virgilio F (2009). Expression of the P2X7 receptor increases the Ca2+ content of the endoplasmic reticulum, activates NFATc1, and protects from apoptosis. J Biol Chem.

[78] Amoroso F, Falzoni S, Adinolfi E, Ferrari D, Di Virgilio F (2012). The P2X7 receptor is a key modulator of aerobic glycolysis. Cell Death Dis.

[79] Cronstein BN, Daguma L, Nichols D, Hutchison AJ, Williams M (1990). The adenosine/neutrophil paradox resolved: human neutrophils possess both A1 and A2 receptors that promote chemotaxis and inhibit O2 generation, respectively. J Clin Invest.

[80] Rose FR, Hirschhorn R, Weissmann G, Cronstein BN (1988). Adenosine promotes neutrophil chemotaxis. J Exp Med.

[81] Felsch A, Stocker K, Borchard U (1995). Phorbol ester-stimulated adherence of neutrophils to endothelial cells is reduced by adenosine A2 receptor agonists. J Immunol.

[82] Thiel M, Chouker A (1995). Acting via A2 receptors, adenosine inhibits the production of tumor necrosis factor-alpha of endotoxin-stimulated human polymorphonuclear leukocytes. J Lab Clin Med.

[83] Salmon JE, Cronstein BN (1990). Fc gamma receptor-mediated functions in neutrophils are modulated by adenosine receptor occupancy. A1 receptors are stimulatory and A2 receptors are inhibitory. J Immunol.

[84] Zalavary S, Stendahl O, Bengtsson T (1994). The role of cyclic AMP, calcium and filamentous actin in adenosine modulation of Fc receptor-mediated phagocytosis in human neutrophils. Biochim Biophys Acta.

[85] Zalavary S, Bengtsson T (1998). Adenosine inhibits actin dynamics in human neutrophils: evidence for the involvement of cAMP. Eur J Cell Biol.

[86] Xu X, Zheng S, Xiong Y, Wang X, Qin W, Zhang H, Sun B (2017). Adenosine effectively restores endotoxin-induced inhibition of human neutrophil chemotaxis via A1 receptor-p38 pathway. Inflamm Res.

[87] Chen L, Fredholm BB, Jondal M (2008). Adenosine, through the A1 receptor, inhibits vesicular MHC class I cross-presentation by resting DC. Mol Immunol.

[88] Schnurr M, Toy T, Shin A, Hartmann G, Rothenfusser S, Soellner J, Davis ID, Cebon J, Maraskovsky E (2004). Role of adenosine receptors in regulating chemotaxis and cytokine production of plasmacytoid dendritic cells. Blood.

[89] Figueiro F, Muller L, Funk S, Jackson EK, Battastini AM, Whiteside TL (2016). Phenotypic and functional characteristics of CD39high human regulatory B cells (Breg). Oncoimmunology.

[90] Ohtsuka T, Changelian PS, Bouis D, Noon K, Harada H, Lama VN, Pinsky DJ (2010)Ecto-5'-nucleotidase(CD73) attenuates allograft airway rejection through adenosine 2A receptor stimulation. J Immunol 185(2):1321–1329. 10.4049/jimmunol.0901847 10.4049/jimmunol.0901847 PMC3014992 20548026

[91] Link AA, Kino T, Worth JA, McGuire JL, Crane ML, Chrousos GP, Wilder RL, Elenkov IJ (2000). Ligand-activation of the adenosine A2a receptors inhibits IL-12 production by human monocytes. J Immunol.

[92] Zhang JG, Hepburn L, Cruz G, Borman RA, Clark KL (2005). The role of adenosine A2A and A2B receptors in the regulation of TNF-alpha production by human monocytes. Biochem Pharmacol.

[93] Thiel M, Chambers JD, Chouker A, Fischer S, Zourelidis C, Bardenheuer HJ, Arfors KE, Peter K (1996). Effect of adenosine on the expression of beta(2) integrins and L-selectin of human polymorphonuclear leukocytes in vitro. J Leukoc Biol.

[94] Wollner A, Wollner S, Smith JB (1993). Acting via A2 receptors, adenosine inhibits the upregulation of Mac-1 (Cd11b/CD18) expression on FMLP-stimulated neutrophils. Am J Respir Cell Mol Biol.

[95] Cadieux JS, Leclerc P, St-Onge M, Dussault AA, Laflamme C, Picard S, Ledent C, Borgeat P, Pouliot M (2005). Potentiation of neutrophil cyclooxygenase-2 by adenosine: an early anti-inflammatory signal. J Cell Sci.

[96] Sullivan GW, Linden J, Buster BL, Scheld WM (1999). Neutrophil A2A adenosine receptor inhibits inflammation in a rat model of meningitis: synergy with the type IV phosphodiesterase inhibitor, rolipram. J Infect Dis.

[97] McColl SR, St-Onge M, Dussault AA, Laflamme C, Bouchard L, Boulanger J, Pouliot M (2006). Immunomodulatory impact of the A2A adenosine receptor on the profile of chemokines produced by neutrophils. FASEB J.

[98] Krump E, Lemay G, Borgeat P (1996). Adenosine A2 receptor-induced inhibition of leukotriene B4 synthesis in whole blood ex vivo. Br J Pharmacol.

[99] Krump E, Picard S, Mancini J, Borgeat P (1997). Suppression of leukotriene B4 biosynthesis by endogenous adenosine in ligand-activated human neutrophils. J Exp Med.

[100] Surette ME, Krump E, Picard S, Borgeat P (1999). Activation of leukotriene synthesis in human neutrophils by exogenous arachidonic acid: inhibition by adenosine A(2a) receptor agonists and crucial role of autocrine activation by leukotriene B(4). Mol Pharmacol.

[101] Flamand N, Boudreault S, Picard S, Austin M, Surette ME, Plante H, Krump E, Vallee MJ, Gilbert C, Naccache P, Laviolette M, Borgeat P (2000). Adenosine, a potent natural suppressor of arachidonic acid release and leukotriene biosynthesis in human neutrophils. Am J Respir Crit Care Med.

[102] Flamand N, Surette ME, Picard S, Bourgoin S, Borgeat P (2002). Cyclic AMP-mediated inhibition of 5-lipoxygenase translocation and leukotriene biosynthesis in human neutrophils. Mol Pharmacol.

[103] Sullivan GW, Rieger JM, Scheld WM, Macdonald TL, Linden J (2001). Cyclic AMP-dependent inhibition of human neutrophil oxidative activity by substituted 2-propynylcyclohexyl adenosine A(2A) receptor agonists. Br J Pharmacol.

[104] Anderson R, Visser SS, Ramafi G, Theron AJ (2000). Accelerated resequestration of cytosolic calcium and suppression of the pro-inflammatory activities of human neutrophils by CGS 21680 in vitro. Br J Pharmacol.

[105] Richter J (1992). Effect of adenosine analogues and cAMP-raising agents on TNF-, GM-CSF-, and chemotactic peptide-induced degranulation in single adherent neutrophils. J Leukoc Biol.

[106] Visser SS, Theron AJ, Ramafi G, Ker JA, Anderson R (2000). Apparent involvement of the A(2A) subtype adenosine receptor in the anti-inflammatory interactions of CGS 21680, cyclopentyladenosine, and IB-MECA with human neutrophils. Biochem Pharmacol.

[107] Walker BA, Rocchini C, Boone RH, Ip S, Jacobson MA (1997). Adenosine A2a receptor activation delays apoptosis in human neutrophils. J Immunol.

[108] Liu YW, Yang T, Zhao L, Ni Z, Yang N, He F, Dai SS (2016). Activation of adenosine 2A receptor inhibits neutrophil apoptosis in an autophagy-dependent manner in mice with systemic inflammatory response syndrome. Sci Rep.

[109] Ryzhov S, Goldstein AE, Matafonov A, Zeng D, Biaggioni I, Feoktistov I (2004). Adenosine-activated mast cells induce IgE synthesis by B lymphocytes: an A2B-mediated process involving Th2 cytokines IL-4 and IL-13 with implications for asthma. J Immunol.

[110] Kreckler LM, Gizewski E, Wan TC, Auchampach JA (2009). Adenosine suppresses lipopolysaccharide-induced tumor necrosis factor-alpha production by murine macrophages through a protein kinase A- and exchange protein activated by cAMP-independent signaling pathway. J Pharmacol Exp Ther.

[111] Hassanian SM, Dinarvand P, Rezaie AR (2014). Adenosine regulates the proinflammatory signaling function of thrombin in endothelial cells. J Cell Physiol.

[112] Zarek PE, Huang CT, Lutz ER, Kowalski J, Horton MR, Linden J, Drake CG, Powell JD (2008) A2A receptor signaling promotes peripheral tolerance by inducing T-cell anergy and the generation of adaptive regulatory T cells. Blood 111 (1):251-259. 10.1182/blood-2007-03-081646 10.1182/blood-2007-03-081646 PMC2200810 17909080

[113] Csoka B, Himer L, Selmeczy Z, Vizi ES, Pacher P, Ledent C, Deitch EA, Spolarics Z, Nemeth ZH, Hasko G (2008). Adenosine A2A receptor activation inhibits T helper 1 and T helper 2 cell development and effector function. FASEB J.

[114] Alam MS, Kurtz CC, Wilson JM, Burnette BR, Wiznerowicz EB, Ross WG, Rieger JM, Figler RA, Linden J, Crowe SE, Ernst PB (2009). A2A adenosine receptor (AR) activation inhibits pro-inflammatory cytokine production by human CD4+ helper T cells and regulates Helicobacter-induced gastritis and bacterial persistence. Mucosal Immunol.

[115] Hasko G, Kuhel DG, Chen JF, Schwarzschild MA, Deitch EA, Mabley JG, Marton A, Szabo C (2000). Adenosine inhibits IL-12 and TNF-[alpha] production via adenosine A2a receptor-dependent and independent mechanisms. FASEB J.

[116] Erdmann AA, Gao ZG, Jung U, Foley J, Borenstein T, Jacobson KA, Fowler DH (2005). Activation of Th1 and Tc1 cell adenosine A2A receptors directly inhibits IL-2 secretion in vitro and IL-2-driven expansion in vivo. Blood.

[117] Lappas CM, Rieger JM, Linden J (2005). A2A adenosine receptor induction inhibits IFN-gamma production in murine CD4+ T cells. J Immunol.

[118] Ohta A, Kini R, Ohta A, Subramanian M, Madasu M, Sitkovsky M (2012). The development and immunosuppressive functions of CD4(+) CD25(+) FoxP3(+) regulatory T cells are under influence of the adenosine-A2A adenosine receptor pathway. Front Immunol.

[119] Huang X, He Y, Chen Y, Wu P, Gui D, Cai H, Chen A, Chen M, Dai C, Yao D, Wang L (2016). Baicalin attenuates bleomycin-induced pulmonary fibrosis via adenosine A2a receptor related TGF-beta1-inducedERK1/2 signaling pathway. BMC Pulm Med.

[120] Guillén-Gómez E, Silva I, Serra N, Caballero F, Leal J, Breda A, San Martín R, Pastor-Anglada M, Ballarín JA, Guirado L, Díaz-Encarnación MM (2020) From inflammation to the onset of fibrosis through A(2A) receptors in kidneys from deceased donors. Int J Mol Sci 21(22). 10.3390/ijms21228826 10.3390/ijms21228826 PMC7700266 33233484

[121] Shi L, Feng M, Du S, Wei X, Song H, Yixin X, Song J, Wenxian G (2019). Adenosine generated by regulatory T cells induces CD8(+) T cell exhaustion in gastric cancer through A2aR pathway. Biomed Res Int.

[122] Barbera-Cremades M, Baroja-Mazo A, Pelegrin P (2016). Purinergic signaling during macrophage differentiation results in M2 alternative activated macrophages. J Leukoc Biol.

[123] Csoka B, Selmeczy Z, Koscso B, Nemeth ZH, Pacher P, Murray PJ, Kepka-Lenhart D, Morris SM, Gause WC, Leibovich SJ, Hasko G (2012). Adenosine promotes alternative macrophage activation via A2A and A2B receptors. FASEB J.

[124] Ferrante CJ, Pinhal-Enfield G, Elson G, Cronstein BN, Hasko G, Outram S, Leibovich SJ (2013). The adenosine-dependent angiogenic switch of macrophages to an M2-like phenotype is independent of interleukin-4 receptor alpha (IL-4Ralpha) signaling. Inflammation.

[125] Koscso B, Csoka B, Kokai E, Nemeth ZH, Pacher P, Virag L, Leibovich SJ, Hasko G (2013). Adenosine augments IL-10-induced STAT3 signaling in M2c macrophages. J Leukoc Biol.

[126] Hasko G, Pacher P (2012). Regulation of macrophage function by adenosine. Arterioscler Thromb Vasc Biol.

[127] Koroskenyi K, Kiss B, Szondy Z (2016). Adenosine A2A receptor signaling attenuates LPS-induced pro-inflammatory cytokine formation of mouse macrophages by inducing the expression of DUSP1. Biochim Biophys Acta.

[128] Majumdar S, Aggarwal BB (2003). Adenosine suppresses activation of nuclear factor-kappaB selectively induced by tumor necrosis factor in different cell types. Oncogene.

[129] Mirakaj V, Thix CA, Laucher S, Mielke C, Morote-Garcia JC, Schmit MA, Henes J, Unertl KE, Kohler D, Rosenberger P (2010). Netrin-1 dampens pulmonary inflammation during acute lung injury. Am J Respir Crit Care Med.

[130] Aherne CM, Collins CB, Masterson JC, Tizzano M, Boyle TA, Westrich JA, Parnes JA, Furuta GT, Rivera-Nieves J, Eltzschig HK (2012). Neuronal guidance molecule netrin-1 attenuates inflammatory cell trafficking during acute experimental colitis. Gut.

[131] van der Hoeven D, Wan TC, Gizewski ET, Kreckler LM, Maas JE, Van Orman J, Ravid K, Auchampach JA (2011). A role for the low-affinity A2B adenosine receptor in regulating superoxide generation by murine neutrophils. J Pharmacol Exp Ther.

[132] Nemeth ZH, Lutz CS, Csoka B, Deitch EA, Leibovich SJ, Gause WC, Tone M, Pacher P, Vizi ES, Hasko G (2005). Adenosine augments IL-10 production by macrophages through an A2B receptor-mediated posttranscriptional mechanism. J Immunol.

[133] Novitskiy SV, Ryzhov S, Zaynagetdinov R, Goldstein AE, Huang Y, Tikhomirov OY, Blackburn MR, Biaggioni I, Carbone DP, Feoktistov I, Dikov MM (2008). Adenosine receptors in regulation of dendritic cell differentiation and function. Blood.

[134] Wilson JM, Kurtz CC, Black SG, Ross WG, Alam MS, Linden J, Ernst PB (2011). The A2B adenosine receptor promotes Th17 differentiation via stimulation of dendritic cell IL-6. J Immunol.

[135] Liang D, Zuo A, Shao H, Chen M, Kaplan HJ, Sun D (2015). A2B adenosine receptor activation switches differentiation of bone marrow cells to a CD11c(+)Gr-1(+) dendritic cell subset that promotes the Th17 response. Immun Inflamm Dis.

[136] Karmouty-Quintana H, Philip K, Acero LF, Chen NY, Weng T, Molina JG, Luo F, Davies J, Le NB, Bunge I, Volcik KA, Le TT, Johnston RA, Xia Y, Eltzschig HK, Blackburn MR (2015). Deletion of ADORA2B from myeloid cells dampens lung fibrosis and pulmonary hypertension. FASEB J.

[137] Eckle T, Grenz A, Laucher S, Eltzschig HK (2008). A2B adenosine receptor signaling attenuates acute lung injury by enhancing alveolar fluid clearance in mice. J Clin Invest.

[138] Grant MB, Tarnuzzer RW, Caballero S, Ozeck MJ, Davis MI, Spoerri PE, Feoktistov I, Biaggioni I, Shryock JC, Belardinelli L (1999). Adenosine receptor activation induces vascular endothelial growth factor in human retinal endothelial cells. Circ Res.

[139] Zhong H, Wu Y, Belardinelli L, Zeng D (2006). A2B adenosine receptors induce IL-19 from bronchial epithelial cells, resulting in TNF-alpha increase. Am J Respir Cell Mol Biol.

[140] Wilkinson PF, Farrell FX, Morel D, Law W, Murphy S (2016). Adenosine signaling increases proinflammatory and profibrotic mediators through activation of a functional adenosine 2B receptor in renal fibroblasts. Ann Clin Lab Sci.

[141] Zhou Y, Murthy JN, Zeng D, Belardinelli L, Blackburn MR (2010). Alterations in adenosine metabolism and signaling in patients with chronic obstructive pulmonary disease and idiopathic pulmonary fibrosis. PLoS One.

[142] Huang L, Fan J, Chen YX, Wang JH (2020). Inhibition of A(2B) Adenosine receptor attenuates intestinal injury in a rat model of necrotizing enterocolitis. Mediat Inflamm.

[143] Reyes AWB, Vu SH, Huy TXN, Min W, Lee HJ, Chang HH, Lee JH, Kim S (2020). Adenosine receptor Adora2b antagonism attenuates Brucella abortus 544 infection in professional phagocyte RAW 264.7 cells and BALB/c mice. Vet Microbiol.

[144] Feoktistov I, Ryzhov S, Goldstein AE, Biaggioni I (2003). Mast cell-mediated stimulation of angiogenesis: cooperative interaction between A2B and A3 adenosine receptors. Circ Res.

[145] Joos G, Jakim J, Kiss B, Szamosi R, Papp T, Felszeghy S, Saghy T, Nagy G, Szondy Z (2017). Involvement of adenosine A3 receptors in the chemotactic navigation of macrophages towards apoptotic cells. Immunol Lett.

[146] Chen Y, Corriden R, Inoue Y, Yip L, Hashiguchi N, Zinkernagel A, Nizet V, Insel PA, Junger WG (2006). ATP release guides neutrophil chemotaxis via P2Y2 and A3 receptors. Science.

[147] Inoue Y, Chen Y, Hirsh MI, Yip L, Junger WG (2008). A3 and P2Y2 receptors control the recruitment of neutrophils to the lungs in a mouse model of sepsis. Shock.

[148] Tweedy L, Knecht DA, Mackay GM, Insall RH (2016). Self-generated chemoattractant gradients: attractant depletion extends the range and robustness of chemotaxis. PLoS Biol.

[149] Tweedy L, Susanto O, Insall RH (2016). Self-generated chemotactic gradients-cells steering themselves. Curr Opin Cell Biol.

[150] Dona E, Barry JD, Valentin G, Quirin C, Khmelinskii A, Kunze A, Durdu S, Newton LR, Fernandez-Minan A, Huber W, Knop M, Gilmour D (2013). Directional tissue migration through a self-generated chemokine gradient. Nature.

[151] So H (2016). Where to go: breaking the symmetry in cell motility. PLoS Biol.

[152] Moissoglu K, Majumdar R, Parent CA (2014). Cell migration: sinking in a gradient. Curr Biol.

[153] Lee JY, Jhun BS, Oh YT, Lee JH, Choe W, Baik HH, Ha J, Yoon KS, Kim SS, Kang I (2006). Activation of adenosine A3 receptor suppresses lipopolysaccharide-induced TNF-alpha production through inhibition of PI 3-kinase/Akt and NF-kappaB activation in murine BV2 microglial cells. Neurosci Lett.

[154] Ren T, Qiu Y, Wu W, Feng X, Ye S, Wang Z, Tian T, He Y, Yu C, Zhou Y (2014). Activation of adenosine A3 receptor alleviates TNF-alpha-induced inflammation through inhibition of the NF-kappaB signaling pathway in human colonic epithelial cells. Mediat Inflamm.

[155] Hoskin DW, Butler JJ, Drapeau D, Haeryfar SM, Blay J (2002). Adenosine acts through an A3 receptor to prevent the induction of murine anti-CD3-activated killer T cells. Int J Cancer.

[156] Ferreira-Silva J, Aires ID, Boia R, Ambrósio AF, Santiago AR (2020) Activation of adenosine A(3) receptor inhibits microglia reactivity elicited by elevated pressure. Int J Mol Sci 21(19). 10.3390/ijms21197218 10.3390/ijms21197218 PMC7582754 33007835

[157] Morschl E, Molina JG, Volmer JB, Mohsenin A, Pero RS, Hong JS, Kheradmand F, Lee JJ, Blackburn MR (2008). A3 adenosine receptor signaling influences pulmonary inflammation and fibrosis. Am J Respir Cell Mol Biol.

[158] Ren TH, Lv MM, An XM, Leung WK, Seto WK (2020). Activation of adenosine A3 receptor inhibits inflammatory cytokine production in colonic mucosa of patients with ulcerative colitis by down-regulating the nuclear factor-kappa B signaling. J Dig Dis.

[159] Oury C, Lecut C, Hego A, Wera O, Delierneux C (2015). Purinergic control of inflammation and thrombosis: role of P2X1 receptors. Comput Struct Biotechnol J.

[160] Alarcón P, Manosalva C, Quiroga J, Belmar I, Álvarez K, Díaz G, Taubert A, Hermosilla C, Carretta MD, Burgos RA, Hidalgo MA (2020). Oleic and linoleic acids induce the release of neutrophil extracellular traps via pannexin 1-dependent ATP release and P2X1 receptor activation. Front Vet Sci.

[161] Woehrle T, Yip L, Elkhal A, Sumi Y, Chen Y, Yao Y, Insel PA, Junger WG (2010). Pannexin-1 hemichannel-mediated ATP release together with P2X1 and P2X4 receptors regulate T-cell activation at the immune synapse. Blood.

[162] Bulanova E, Budagian V, Orinska Z, Koch-Nolte F, Haag F, Bulfone-Paus S (2009). ATP induces P2X7 receptor-independent cytokine and chemokine expression through P2X1 and P2X3 receptors in murine mast cells (Article retracted in 2011 due to figure irregularities). J Leukoc Biol.

[163] Manohar M, Hirsh MI, Chen Y, Woehrle T, Karande AA, Junger WG (2012). ATP release and autocrine signaling through P2X4 receptors regulate gammadelta T cell activation. J Leukoc Biol.

[164] Vazquez-Villoldo N, Domercq M, Martin A, Llop J, Gomez-Vallejo V, Matute C (2014). P2X4 receptors control the fate and survival of activated microglia. Glia.

[165] Ledderose C, Liu K, Kondo Y, Slubowski CJ, Dertnig T, Denicoló S, Arbab M, Hubner J, Konrad K, Fakhari M, Lederer JA, Robson SC, Visner GA, Junger WG (2018). Purinergic P2X4 receptors and mitochondrial ATP production regulate T cell migration. J Clin Invest.

[166] Nguyen HM, di Lucente J, Chen YJ, Cui Y, Ibrahim RH, Pennington MW, Jin LW, Maezawa I, Wulff H (2020). Biophysical basis for Kv1.3 regulation of membrane potential changes induced by P2X4-mediated calcium entry in microglia. Glia.

[167] Ledderose C, Bromberger S, Slubowski CJ, Sueyoshi K, Junger WG (2020) Frontline Science: P2Y11 receptors support T cell activation by directing mitochondrial trafficking to the immune synapse. J Leukoc Biol. 10.1002/jlb.2hi0520-191r 10.1002/JLB.2HI0520-191R PMC8772287 32531829

[168] Ledderose C, Bromberger S, Slubowski CJ, Sueyoshi K, Aytan D, Shen Y, Junger WG (2020) The purinergic receptor P2Y11 choreographs the polarization, mitochondrial metabolism, and migration of T lymphocytes. Sci Signal 13(651). 10.1126/scisignal.aba3300 10.1126/scisignal.aba3300 PMC8826773 32994212

[169] Cekic C, Linden J (2016). Purinergic regulation of the immune system. Nat Rev Immunol.

[170] Lee BH, Hwang DM, Palaniyar N, Grinstein S, Philpott DJ, Hu J (2012). Activation of P2X(7) receptor by ATP plays an important role in regulating inflammatory responses during acute viral infection. PLoS One.

[171] Jo EK, Kim JK, Shin DM, Sasakawa C (2016). Molecular mechanisms regulating NLRP3 inflammasome activation. Cell Mol Immunol.

[172] Latz E, Xiao TS, Stutz A (2013). Activation and regulation of the inflammasomes. Nat Rev Immunol.

[173] Sakaki H, Fujiwaki T, Tsukimoto M, Kawano A, Harada H, Kojima S (2013). P2X4 receptor regulates P2X7 receptor-dependent IL-1beta and IL-18 release in mouse bone marrow-derived dendritic cells. Biochem Biophys Res Commun.

[174] Luna-Gomes T, Santana PT, Coutinho-Silva R (2015). Silica-induced inflammasome activation in macrophages: role of ATP and P2X7 receptor. Immunobiology.

[175] Karmakar M, Katsnelson MA, Dubyak GR, Pearlman E (2016). Neutrophil P2X7 receptors mediate NLRP3 inflammasome-dependent IL-1beta secretion in response to ATP. Nat Commun.

[176] Eleftheriadis T, Pissas G, Karioti A, Antoniadi G, Golfinopoulos S, Liakopoulos V, Mamara A, Speletas M, Koukoulis G, Stefanidis I (2013). Uric acid induces caspase-1 activation, IL-1beta secretion and P2X7 receptor dependent proliferation in primary human lymphocytes. Hippokratia.

[177] Jeong YH, Walsh MC, Yu J, Shen H, Wherry EJ, Choi Y (2020). Mice lacking the purinergic receptor P2X5 exhibit defective inflammasome activation and early susceptibility to listeria monocytogenes. J Immunol.

[178] Wiley JS, Gu BJ (2012). A new role for the P2X7 receptor: a scavenger receptor for bacteria and apoptotic cells in the absence of serum and extracellular ATP. Purinergic Signal.

[179] Gu BJ, Saunders BM, Petrou S, Wiley JS (2011). P2X(7) is a scavenger receptor for apoptotic cells in the absence of its ligand, extracellular ATP. J Immunol.

[180] Brandao-Burch A, Key ML, Patel JJ, Arnett TR, Orriss IR (2012). The P2X7 receptor is an important regulator of extracellular ATP levels. Front Endocrinol (Lausanne).

[181] Gutierrez-Martin Y, Bustillo D, Gomez-Villafuertes R, Sanchez-Nogueiro J, Torregrosa-Hetland C, Binz T, Gutierrez LM, Miras-Portugal MT, Artalejo AR (2011). P2X7 receptors trigger ATP exocytosis and modify secretory vesicle dynamics in neuroblastoma cells. J Biol Chem.

[182] Suadicani SO, Brosnan CF, Scemes E (2006). P2X7 receptors mediate ATP release and amplification of astrocytic intercellular Ca2+ signaling. J Neurosci.

[183] Henriquez M, Herrera-Molina R, Valdivia A, Alvarez A, Kong M, Munoz N, Eisner V, Jaimovich E, Schneider P, Quest AF, Leyton L (2011). ATP release due to Thy-1-integrin binding induces P2X7-mediated calcium entry required for focal adhesion formation. J Cell Sci.

[184] Mishra A, Chintagari NR, Guo Y, Weng T, Su L, Liu L (2011). Purinergic P2X7 receptor regulates lung surfactant secretion in a paracrine manner. J Cell Sci.

[185] Ohshima Y, Tsukimoto M, Takenouchi T, Harada H, Suzuki A, Sato M, Kitani H, Kojima S (2010). gamma-Irradiation induces P2X(7) receptor-dependent ATP release from B16 melanoma cells. Biochim Biophys Acta.

[186] Johnsen B, Kaschubowski KE, Nader S, Schneider E, Nicola JA, Fliegert R, Wolf IMA, Guse AH, Nikolaev VO, Koch-Nolte F, Haag F (2019). P2X7-mediated ATP secretion is accompanied by depletion of cytosolic ATP. Purinergic Signal.

[187] Sáez PJ, Vargas P, Shoji KF, Harcha PA, Lennon-Duménil AM, Sáez JC (2017) ATP promotes the fast migration of dendritic cells through the activity of pannexin 1 channels and P2X(7) receptors. Sci Signal 10(506). 10.1126/scisignal.aah7107 10.1126/scisignal.aah7107 29162744

[188] Gilbert SM, Oliphant CJ, Hassan S, Peille AL, Bronsert P, Falzoni S, Di Virgilio F, McNulty S, Lara R (2019). ATP in the tumour microenvironment drives expression of nfP2X7, a key mediator of cancer cell survival. Oncogene.

[189] Pellegatti P, Falzoni S, Donvito G, Lemaire I, Di Virgilio F (2011). P2X7 receptor drives osteoclast fusion by increasing the extracellular adenosine concentration. FASEB J.

[190] Schenk U, Frascoli M, Proietti M, Geffers R, Traggiai E, Buer J, Ricordi C, Westendorf AM, Grassi F (2011). ATP inhibits the generation and function of regulatory T cells through the activation of purinergic P2X receptors. Sci Signal.

[191] Figliuolo VR, Savio LEB, Safya H, Nanini H, Bernardazzi C, Abalo A, de Souza HSP, Kanellopoulos J, Bobe P, Coutinho C, Coutinho-Silva R (2017). P2X7 receptor promotes intestinal inflammation in chemically induced colitis and triggers death of mucosal regulatory T cells. Biochim Biophys Acta Mol basis Dis.

[192] Koo TY, Lee JG, Yan JJ, Jang JY, Ju KD, Han M, Oh KH, Ahn C, Yang J (2017). The P2X7 receptor antagonist, oxidized adenosine triphosphate, ameliorates renal ischemia-reperfusion injury by expansion of regulatory T cells. Kidney Int.

[193] Frascoli M, Marcandalli J, Schenk U, Grassi F (2012). Purinergic P2X7 receptor drives T cell lineage choice and shapes peripheral γδ cells. J Immunol.

[194] Romagnani A, Rottoli E, Mazza EMC, Rezzonico-Jost T, De Ponte CB, Proietti M, Perotti M, Civanelli E, Perruzza L, Catapano AL, Baragetti A, Tenedini E, Tagliafico E, Falzoni S, Di Virgilio F, Norata GD, Bicciato S, Grassi F (2020). P2X7 Receptor activity limits accumulation of T cells within tumors. Cancer Res.

[195] Verhoef PA, Estacion M, Schilling W, Dubyak GR (2003). P2X7 receptor-dependent blebbing and the activation of Rho-effector kinases, caspases, and IL-1 beta release. J Immunol.

[196] Pfeiffer ZA, Aga M, Prabhu U, Watters JJ, Hall DJ, Bertics PJ (2004). The nucleotide receptor P2X7 mediates actin reorganization and membrane blebbing in RAW 264.7 macrophages via p38 MAP kinase and Rho. J Leukoc Biol.

[197] Gu BJ, Wiley JS (2006). Rapid ATP-induced release of matrix metalloproteinase 9 is mediated by the P2X7 receptor. Blood.

[198] de Torre-Minguela C, Barbera-Cremades M, Gomez AI, Martin-Sanchez F, Pelegrin P (2016). Macrophage activation and polarization modify P2X7 receptor secretome influencing the inflammatory process. Sci Rep.

[199] Csoka B, Nemeth ZH, Toro G, Idzko M, Zech A, Koscso B, Spolarics Z, Antonioli L, Cseri K, Erdelyi K, Pacher P, Hasko G (2015). Extracellular ATP protects against sepsis through macrophage P2X7 purinergic receptors by enhancing intracellular bacterial killing. FASEB J.

[200] Wareham KJ, Seward EP (2016). P2X7 receptors induce degranulation in human mast cells. Purinergic Signal.

[201] Kawamura H, Aswad F, Minagawa M, Govindarajan S, Dennert G (2006). P2X7 receptors regulate NKT cells in autoimmune hepatitis. J Immunol.

[202] Pupovac A, Foster CM, Sluyter R (2013). Human P2X7 receptor activation induces the rapid shedding of CXCL16. Biochem Biophys Res Commun.

[203] Pupovac A, Geraghty NJ, Watson D, Sluyter R (2015). Activation of the P2X7 receptor induces the rapid shedding of CD23 from human and murine B cells. Immunol Cell Biol.

[204] Huang SW, Walker C, Pennock J, Else K, Muller W, Daniels MJ, Pellegrini C, Brough D, Lopez-Castejon G, Cruickshank SM (2017). P2X7 receptor-dependent tuning of gut epithelial responses to infection. Immunol Cell Biol.

[205] Yip L, Woehrle T, Corriden R, Hirsh M, Chen Y, Inoue Y, Ferrari V, Insel PA, Junger WG (2009). Autocrine regulation of T-cell activation by ATP release and P2X7 receptors. FASEB J.

[206] Proietti M, Cornacchione V, Rezzonico Jost T, Romagnani A, Faliti CE, Perruzza L, Rigoni R, Radaelli E, Caprioli F, Preziuso S, Brannetti B, Thelen M, McCoy KD, Slack E, Traggiai E, Grassi F (2014). ATP-gated ionotropic P2X7 receptor controls follicular T helper cell numbers in Peyer's patches to promote host-microbiota mutualism. Immunity.

[207] Hubert S, Rissiek B, Klages K, Huehn J, Sparwasser T, Haag F, Koch-Nolte F, Boyer O, Seman M, Adriouch S (2010). Extracellular NAD+ shapes the Foxp3+ regulatory T cell compartment through the ART2-P2X7 pathway. J Exp Med.

[208] Wilhelm K, Ganesan J, Muller T, Durr C, Grimm M, Beilhack A, Krempl CD, Sorichter S, Gerlach UV, Juttner E, Zerweck A, Gartner F, Pellegatti P, Di Virgilio F, Ferrari D, Kambham N, Fisch P, Finke J, Idzko M, Zeiser R (2010). Graft-versus-host disease is enhanced by extracellular ATP activating P2X7R. Nat Med.

[209] Mishra A, Guo Y, Zhang L, More S, Weng T, Chintagari NR, Huang C, Liang Y, Pushparaj S, Gou D, Breshears M, Liu L (2016) A critical role for P2X7 receptor-induced VCAM-1 shedding and neutrophil infiltration during acute lung injury. JImmunol 10.4049/jimmunol.1501041 PMC5026933 27559050

[210] Semenova S, Shatrova A, Vassilieva I, Shamatova M, Pugovkina N, Negulyaev Y (2020). Adenosine-5'-triphosphate suppresses proliferation and migration capacity of human endometrial stem cells. J Cell Mol Med.

[211] Wang J, Liu S, Nie Y, Wu B, Wu Q, Song M, Tang M, Xiao L, Xu P, Tan X, Zhang L, Li G, Liang S, Zhang C (2015). Activation of P2X7 receptors decreases the proliferation of murine luteal cells. Reprod Fertil Dev.

[212] Wang CM, Chang YY, Sun SH (2003). Activation of P2X7 purinoceptor-stimulated TGF-beta 1 mRNA expression involves PKC/MAPK signalling pathway in a rat brain-derived type-2 astrocyte cell line, RBA-2. Cell Signal.

[213] Qu LP, Xue H, Yuan P, Zhou L, Yao T, Huang Y, Lu LM (2009). Adenosine 5'-triphosphate stimulates the increase of TGF-beta1 in rat mesangial cells under high-glucose conditions via reactive oxygen species and ERK1/2. Acta Pharmacol Sin.

[214] Fan X, Ma W, Zhang Y, Zhang L (2020). P2X7 receptor (P2X7R) of microglia mediates neuroinflammation by regulating (NOD)-like receptor protein 3 (NLRP3)inflammasome-dependent inflammation after spinal cord injury. Med Sci Monit.

[215] Wu H, Jiang M, Liu Q, Wen F, Nie Y (2020). lncRNA uc.48+ regulates immune and inflammatory reactions mediated by the P2X(7) receptor in type 2 diabetic mice. Exp Ther Med.

[216] Wang W, Hu D, Feng Y, Wu C, Song Y, Liu W, Li A, Wang Y, Chen K, Tian M, Xiao F, Zhang Q, Chen W, Pan P, Wan P, Liu Y, Lan H, Wu K, Wu J (2020). Paxillin mediates ATP-induced activation of P2X7 receptor and NLRP3 inflammasome. BMC Biol.

[217] Zhang C, Qin J, Zhang S, Zhang N, Tan B, Siwko S, Zhang Y, Wang Q, Chen J, Qian M, Liu M, Du B (2020). ADP/P2Y(1) aggravates inflammatory bowel disease through ERK5-mediated NLRP3 inflammasome activation. Mucosal Immunol.

[218] Alarcón-Vila C, Baroja-Mazo A, de Torre-Minguela C, Martínez CM, Martínez-García JJ, Martínez-Banaclocha H, García-Palenciano C, Pelegrin P (2020) CD14 release induced by P2X7 receptor restricts inflammation and increases survival during sepsis. Elife 9. 10.7554/eLife.60849 10.7554/eLife.60849 PMC7690950 33135636

[219] Jiang M, Cui BW, Wu YL, Zhang Y, Shang Y, Liu J, Yang HX, Qiao CY, Zhan ZY, Ye H, Jin Q, Nan JX, Lian LH (2020). P2X7R orchestrates the progression of murine hepatic fibrosis by making a feedback loop from macrophage to hepatic stellate cells. Toxicol Lett.

[220] Xu SL, Lin Y, Liu W, Zhu XZ, Liu D, Tong ML, Liu LL, Lin LR (2020). The P2X7 receptor mediates NLRP3-dependent IL-1β secretion and promotes phagocytosis in the macrophage response to Treponema pallidum. Int Immunopharmacol.

[221] Kahlenberg JM, Carmona-Rivera C, Smith CK, Kaplan MJ (2013). Neutrophil extracellular trap-associated protein activation of the NLRP3 inflammasome is enhanced in lupus macrophages. J Immunol.

[222] Pelegrin P, Surprenant A (2006). Pannexin-1 mediates large pore formation and interleukin-1beta release by the ATP-gated P2X7 receptor. EMBO J.

[223] Suh BC, Kim JS, Namgung U, Ha H, Kim KT (2001). P2X7 nucleotide receptor mediation of membrane pore formation and superoxide generation in human promyelocytes and neutrophils. J Immunol.

[224] Tsukimoto M, Maehata M, Harada H, Ikari A, Takagi K, Degawa M (2006). P2X7 receptor-dependent cell death is modulated during murine T cell maturation and mediated by dual signaling pathways. J Immunol.

[225] Aswad F, Dennert G (2006). P2X7 receptor expression levels determine lethal effects of a purine based danger signal in T lymphocytes. Cell Immunol.

[226] Scheuplein F, Schwarz N, Adriouch S, Krebs C, Bannas P, Rissiek B, Seman M, Haag F, Koch-Nolte F (2009). NAD+ and ATP released from injured cells induce P2X7-dependent shedding of CD62L and externalization of phosphatidylserine by murine T cells. J Immunol.

[227] Surprenant A, Rassendren F, Kawashima E, North RA, Buell G (1996). The cytolytic P2Z receptor for extracellular ATP identified as a P2X receptor (P2X7). Science.

[228] Shen D, Shen X, Schwarz W, Grygorczyk R, Wang L (2020). P2Y(13) and P2X(7) receptors modulate mechanically induced adenosine triphosphate release from mast cells. Exp Dermatol.

[229] Nylander S, Mattsson C, Ramstrom S, Lindahl TL (2004). Synergistic action between inhibition of P2Y12/P2Y1 and P2Y12/thrombin in ADP- and thrombin-induced human platelet activation. Br J Pharmacol.

[230] Anderson R, Theron AJ, Steel HC, Nel JG, Tintinger GR (2020)ADP-mediated upregulation of expression of CD62P on human platelets is critically dependent on co-activation of P2Y1 and P2Y12 receptors. Pharmaceuticals (Basel) 13(12). 10.3390/ph13120420 10.3390/ph13120420 PMC7760858 33255391

[231] Muller T, Fay S, Vieira RP, Karmouty-Quintana H, Cicko S, Ayata K, Zissel G, Goldmann T, Lungarella G, Ferrari D, Di Virgilio F, Robaye B, Boeynaems JM, Blackburn MR, Idzko M (2017) The purinergic receptor subtype P2Y2 mediates chemotaxis of neutrophils and fibroblasts in fibrotic lung disease. Oncotarget. 10.18632/oncotarget.16414 10.18632/oncotarget.16414 PMC5482630 28415591

[232] Muller T, Robaye B, Vieira RP, Ferrari D, Grimm M, Jakob T, Martin SF, Di Virgilio F, Boeynaems JM, Virchow JC, Idzko M (2010). The purinergic receptor P2Y2 receptor mediates chemotaxis of dendritic cells and eosinophils in allergic lung inflammation. Allergy.

[233] Vanderstocken G, Bondue B, Horckmans M, Di Pietrantonio L, Robaye B, Boeynaems JM, Communi D (2010). P2Y2 receptor regulates VCAM-1 membrane and soluble forms and eosinophil accumulation during lung inflammation. J Immunol.

[234] de la Rosa G, Gómez AI, Baños MC, Pelegrín P (2020) Signaling through purinergic receptor P2Y(2) enhances macrophage IL-1β production. Int J Mol Sci 21(13). 10.3390/ijms21134686 10.3390/ijms21134686 PMC7370188 32630144

[235] Thorstenberg ML, Martins MDA, Figliuolo V, Silva CLM, Savio LEB, Coutinho-Silva R (2020). P2Y(2) Receptor induces L. amazonensis infection control in a mechanism dependent on caspase-1 activation and IL-1β secretion. Mediat Inflamm.

[236] Ualiyeva S, Hallen N, Kanaoka Y, Ledderose C, Matsumoto I, Junger WG, Barrett NA, Bankova LG (2020) Airway brush cells generate cysteinyl leukotrienes through the ATP sensor P2Y2. Sci Immunol 5(43). 10.1126/sciimmunol.aax7224 10.1126/sciimmunol.aax7224 PMC7176051 31953256

[237] Ohsawa K, Irino Y, Nakamura Y, Akazawa C, Inoue K, Kohsaka S (2007). Involvement of P2X4 and P2Y12 receptors in ATP-induced microglial chemotaxis. Glia.

[238] Irino Y, Nakamura Y, Inoue K, Kohsaka S, Ohsawa K (2008). Akt activation is involved in P2Y12 receptor-mediated chemotaxis of microglia. J Neurosci Res.

[239] Sil P, Hayes CP, Reaves BJ, Breen P, Quinn S, Sokolove J, Rada B (2017). P2Y6 receptor antagonist MRS2578 inhibits neutrophil activation and aggregated neutrophil extracellular trap formation induced by gout-associated monosodium urate crystals. J Immunol.

[240] Uratsuji H, Tada Y, Kawashima T, Kamata M, Hau CS, Asano Y, Sugaya M, Kadono T, Asahina A, Sato S, Tamaki K (2012). P2Y6 receptor signaling pathway mediates inflammatory responses induced by monosodium urate crystals. J Immunol.

[241] Kimura T, Kobayashi S, Hanihara-Tatsuzawa F, Sayama A, MaruYama T, Muta T (2014). Responses of macrophages to the danger signals released from necrotic cells. Int Immunol.

[242] Inoue K (2007). UDP facilitates microglial phagocytosis through P2Y6 receptors. Cell Adhes Migr.

[243] Morioka N, Tokuhara M, Harano S, Nakamura Y, Hisaoka-Nakashima K, Nakata Y (2013). The activation of P2Y6 receptor in cultured spinal microglia induces the production of CCL2 through the MAP kinases-NF-kappaB pathway. Neuropharmacology.

[244] Xu Y, Hu W, Liu Y, Xu P, Li Z, Wu R, Shi X, Tang Y (2016). P2Y6 receptor-mediated microglial phagocytosis in radiation-induced brain injury. Mol Neurobiol.

[245] Zhu J, Wang Z, Zhang N, Ma J, Xu SL, Wang Y, Shen Y, Li YH (2016). Protein interacting C-kinase 1 modulates surface expression of P2Y6 purinoreceptor, actin polymerization and phagocytosis in microglia. Neurochem Res.

[246] Nakano M, Ito K, Yuno T, Soma N, Aburakawa S, Kasai K, Nakamura T, Takami H (2017)UDP/P2Y6 receptor signaling regulates IgE-dependent degranulation in human basophils. Allergol Int. 10.1016/j.alit.2017.02.014 10.1016/j.alit.2017.02.014 28318884

[247] Shin A, Toy T, Rothenfusser S, Robson N, Vorac J, Dauer M, Stuplich M, Endres S, Cebon J, Maraskovsky E, Schnurr M (2008). P2Y receptor signaling regulates phenotype and IFN-alpha secretion of human plasmacytoid dendritic cells. Blood.

[248] Khine AA, Del Sorbo L, Vaschetto R, Voglis S, Tullis E, Slutsky AS, Downey GP, Zhang H (2006). Human neutrophil peptides induce interleukin-8 production through the P2Y6 signaling pathway. Blood.

[249] Grbic DM, Degagne E, Larrivee JF, Bilodeau MS, Vinette V, Arguin G, Stankova J, Gendron FP (2012). P2Y6 receptor contributes to neutrophil recruitment to inflamed intestinal mucosa by increasing CXC chemokine ligand 8 expression in an AP-1-dependent manner in epithelial cells. Inflamm Bowel Dis.

[250] Li R, Tan B, Yan Y, Ma X, Zhang N, Zhang Z, Liu M, Qian M, Du B (2014). Extracellular UDP and P2Y6 function as a danger signal to protect mice from vesicular stomatitis virus infection through an increase in IFN-beta production. J Immunol.

[251] Li Z, He C, Zhang J, Zhang H, Wei H, Wu S, Jiang W (2020). P2Y(6) Deficiency enhances dendritic cell-mediatedTh1/Th17 differentiation and aggravates experimental autoimmune encephalomyelitis. J Immunol.

[252] Wen RX, Shen H, Huang SX, Wang LP, Li ZW, Peng P, Mamtilahun M, Tang YH, Shen FX, Tian HL, Yang GY, Zhang ZJ (2020). P2Y6 receptor inhibition aggravates ischemic brain injury by reducing microglial phagocytosis. CNS Neurosci Ther.

[253] Vaughan KR, Stokes L, Prince LR, Marriott HM, Meis S, Kassack MU, Bingle CD, Sabroe I, Surprenant A, Whyte MK (2007). Inhibition of neutrophil apoptosis by ATP is mediated by the P2Y11 receptor. J Immunol.

[254] Alkayed F, Kashimata M, Koyama N, Hayashi T, Tamura Y, Azuma Y (2012). P2Y11 purinoceptor mediates the ATP-enhanced chemotactic response of rat neutrophils. J Pharmacol Sci.

[255] van der Weyden L, Conigrave AD, Morris MB (2000). Signal transduction and white cell maturation via extracellular ATP and the P2Y11 receptor. Immunol Cell Biol.

[256] Wilkin F, Duhant X, Bruyns C, Suarez-Huerta N, Boeynaems JM, Robaye B (2001). The P2Y11 receptor mediates the ATP-induced maturation of human monocyte-derived dendritic cells. J Immunol.

[257] Schnurr M, Toy T, Stoitzner P, Cameron P, Shin A, Beecroft T, Davis ID, Cebon J, Maraskovsky E (2003). ATP gradients inhibit the migratory capacity of specific human dendritic cell types: implications for P2Y11 receptor signaling. Blood.

[258] Meis S, Hamacher A, Hongwiset D, Marzian C, Wiese M, Eckstein N, Royer HD, Communi D, Boeynaems JM, Hausmann R, Schmalzing G, Kassack MU (2010). NF546 [4,4'-(carbonylbis(imino-3,1-phenylene-carbonylimino-3,1-(4-methyl-phenylene)-car bonylimino))-bis(1,3-xylene-alpha,alpha'-diphosphonic acid) tetrasodium salt] is a non-nucleotide P2Y11 agonist and stimulates release of interleukin-8 from human monocyte-derived dendritic cells. J Pharmacol Exp Ther.

[259] Sakaki H, Tsukimoto M, Harada H, Moriyama Y, Kojima S (2013). Autocrine regulation of macrophage activation via exocytosis of ATP and activation of P2Y11 receptor. PLoS One.

[260] Satonaka H, Nagata D, Takahashi M, Kiyosue A, Myojo M, Fujita D, Ishimitsu T, Nagano T, Nagai R, Hirata Y (2015). Involvement of P2Y12 receptor in vascular smooth muscle inflammatory changes via MCP-1 upregulation and monocyte adhesion. Am J Physiol Heart Circ Physiol.

[261] Ben Addi A, Cammarata D, Conley PB, Boeynaems JM, Robaye B (2010). Role of the P2Y12 receptor in the modulation of murine dendritic cell function by ADP. J Immunol.

[262] Lou N, Takano T, Pei Y, Xavier AL, Goldman SA, Nedergaard M (2016). Purinergic receptor P2RY12-dependent microglial closure of the injured blood-brain barrier. Proc Natl Acad Sci U S A.

[263] Haynes SE, Hollopeter G, Yang G, Kurpius D, Dailey ME, Gan WB, Julius D (2006). The P2Y12 receptor regulates microglial activation by extracellular nucleotides. Nat Neurosci.

[264] Moore CS, Ase AR, Kinsara A, Rao VT, Michell-Robinson M, Leong SY, Butovsky O, Ludwin SK, Seguela P, Bar-Or A, Antel JP (2015). P2Y12 expression and function in alternatively activated human microglia. Neurol Neuroimmunol Neuroinflamm.

[265] Tozaki-Saitoh H, Miyata H, Yamashita T, Matsushita K, Tsuda M, Inoue K (2017). P2Y12 receptors in primary microglia activate nuclear factor of activated T-cell signaling to induce C-C chemokine 3 expression. J Neurochem.

[266] Albayati S, Vemulapalli H, Tsygankov AY, Liverani E (2020) P2Y(12) antagonism results in altered interactions between platelets and regulatory T cells during sepsis. J Leukoc Biol. 10.1002/jlb.3a0220-097r 10.1002/JLB.3A0220-097R 33242353

[267] Jing F, Zhang Y, Long T, He W, Qin G, Zhang D, Chen L, Zhou J (2019). P2Y12 receptor mediates microglial activation via RhoA/ROCK pathway in the trigeminal nucleus caudalis in a mouse model of chronic migraine. J Neuroinflammation.

[268] Wang L, Olivecrona G, Gotberg M, Olsson ML, Winzell MS, Erlinge D (2005). ADP acting on P2Y13 receptors is a negative feedback pathway for ATP release from human red blood cells. Circ Res.

[269] Arase T, Uchida H, Kajitani T, Ono M, Tamaki K, Oda H, Nishikawa S, Kagami M, Nagashima T, Masuda H, Asada H, Yoshimura Y, Maruyama T (2009). The UDP-glucose receptor P2RY14 triggers innate mucosal immunity in the female reproductive tract by inducing IL-8. J Immunol.

[270] Barrett MO, Sesma JI, Ball CB, Jayasekara PS, Jacobson KA, Lazarowski ER, Harden TK (2013). A selective high-affinity antagonist of the P2Y14 receptor inhibits UDP-glucose-stimulated chemotaxis of human neutrophils. Mol Pharmacol.

[271] Li H, Jiang W, Ye S, Zhou M, Liu C, Yang X, Hao K, Hu Q (2020). P2Y(14) receptor has a critical role in acute gouty arthritis by regulating pyroptosis of macrophages. Cell Death Dis.

[272] Kaunitz JD, Yamaguchi DT (2008). TNAP, TrAP, ecto-purinergic signaling, and bone remodeling. J Cell Biochem.

[273] Burnstock G, Arnett TR, Orriss IR (2013). Purinergic signalling in the musculoskeletal system. Purinergic Signal.

[274] Sebastián-Serrano Á, de Diego-García L, Martínez-Frailes C, Ávila J, Zimmermann H, Millán JL, Miras-Portugal MT, Díaz-Hernández M (2015). Tissue-nonspecific alkaline phosphatase regulates purinergic transmission in the central nervous system during development and disease. Comput Struct Biotechnol J.

[275] Zimmermann H (2021). History of ectonucleotidases and their role in purinergic signaling. Biochem Pharmacol.

[276] Jacobson KA, Muller CE (2016). Medicinal chemistry of adenosine, P2Y and P2X receptors. Neuropharmacology.

[277] North RA (2016) P2X receptors. Philos Trans R Soc Lond Ser B Biol Sci 371(1700). 10.1098/rstb.2015.0427 10.1098/rstb.2015.0427 PMC4938027 27377721

[278] Coddou C, Stojilkovic SS, Huidobro-Toro JP (2011). Allosteric modulation of ATP-gated P2X receptor channels. Rev Neurosci.

[279] Coddou C, Yan Z, Obsil T, Huidobro-Toro JP, Stojilkovic SS (2011). Activation and regulation of purinergic P2X receptor channels. Pharmacol Rev.

[280] Sanabria P, Ross E, Ramirez E, Colon K, Hernandez M, Maldonado HM, Silva WI, Jimenez-Rivera CA, Gonzalez FA (2008). P2Y2 receptor desensitization on single endothelial cells. Endothelium.

[281] Schulte G, Fredholm BB (2000). Human adenosine A(1), A(2A), A(2B), and A(3) receptors expressed in Chinese hamster ovary cells all mediate the phosphorylation of extracellular-regulated kinase 1/2. Mol Pharmacol.

[282] Klaasse EC, Ijzerman AP, de Grip WJ, Beukers MW (2008). Internalization and desensitization of adenosine receptors. Purinergic Signal.

[283] North RA (2002). Molecular physiology of P2X receptors. Physiol Rev.

[284] Illes P, Müller CE, Jacobson KA, Grutter T, Nicke A, Fountain SJ, Kennedy C, Schmalzing G, Jarvis MF, Stojilkovic SS, King BF, Di Virgilio F (2021). Update of P2X receptor properties and their pharmacology: IUPHAR review 30. Br J Pharmacol.

[285] Fischer W, Urban N, Immig K, Franke H, Schaefer M (2014). Natural compounds with P2X7 receptor-modulating properties. Purinergic Signal.

[286] Roth-Cross JK, Bender SJ, Weiss SR (2008). Murine coronavirus mouse hepatitis virus is recognized by MDA5 and induces type I interferon in brain macrophages/microglia. J Virol.

[287] Swayne LA, Johnstone SR, Ng CS, Sanchez-Arias JC, Good ME, Penuela S, Lohman AW, Wolpe AG, Laubach VE, Koval M, Isakson BE (2020). Consideration of pannexin 1 channels in COVID-19 pathology and treatment. Am J Phys Lung Cell Mol Phys.

[288] Agier J, Brzezińska-Błaszczyk E, Żelechowska P, Wiktorska M, Pietrzak J, Różalska S (2018). Cathelicidin LL-37 affects surface and intracellular toll-like receptor expression in tissue mast cells. J Immunol Res.

[289] Lohman AW, Leskov IL, Butcher JT, Johnstone SR, Stokes TA, Begandt D, DeLalio LJ, Best AK, Penuela S, Leitinger N, Ravichandran KS, Stokes KY, Isakson BE (2015). Pannexin 1 channels regulate leukocyte emigration through the venous endothelium during acute inflammation. Nat Commun.

[290] Idzko M, Ferrari D, Eltzschig HK (2014). Nucleotide signalling during inflammation. Nature.

[291] Di Virgilio F, Sarti AC, Coutinho-Silva R (2020). Purinergic signaling, DAMPs, and inflammation. Am J Phys Cell Phys.

[292] Venereau E, Ceriotti C, Bianchi ME (2015). DAMPs from cell death to new life. Front Immunol.

[293] McGonagle D, O'Donnell JS, Sharif K, Emery P, Bridgewood C (2020). Immune mechanisms of pulmonary intravascular coagulopathy in COVID-19 pneumonia. Lancet Rheumatol.

[294] Jacobson KA, Delicado EG, Gachet C, Kennedy C, von Kügelgen I, Li B, Miras-Portugal MT, Novak I, Schöneberg T, Perez-Sen R, Thor D, Wu B, Yang Z, Müller CE (2020). Update of P2Y receptor pharmacology: IUPHAR Review 27. Br J Pharmacol.

[295] Eckle T, Fullbier L, Wehrmann M, Khoury J, Mittelbronn M, Ibla J, Rosenberger P, Eltzschig HK (2007). Identification of ectonucleotidases CD39 and CD73 in innate protection during acute lung injury. J Immunol.

[296] Zhao R, Qiao J, Zhang X, Zhao Y, Meng X, Sun D, Peng X (2019). Toll-like receptor-mediated activation of CD39 internalization in BMDCs leads to extracellular ATP accumulation and facilitates P2X7 receptor activation. Front Immunol.

[297] Burnstock G (2016). P2X ion channel receptors and inflammation. Purinergic Signal.

[298] Di Virgilio F, Tang Y, Sarti AC, Rossato M (2020) A rationale for targeting the P2X7 receptor in coronavirus disease 19 (Covid-19). Br J Pharmacol. 10.1111/bph.15138 10.1111/bph.15138 PMC7280564 32441783

[299] Savio LEB, de Andrade MP, da Silva CG, Coutinho-Silva R (2018). The P2X7 receptor in inflammatory diseases: angel or demon?. Front Pharmacol.

[300] Flores RV, Hernandez-Perez MG, Aquino E, Garrad RC, Weisman GA, Gonzalez FA (2005). Agonist-induced phosphorylation and desensitization of the P2Y2 nucleotide receptor. Mol Cell Biochem.

[301] Sromek SM, Harden TK (1998). Agonist-induced internalization of the P2Y2 receptor. Mol Pharmacol.

[302] Hotchkiss RS, Monneret G, Payen D (2013). Sepsis-induced immunosuppression: from cellular dysfunctions to immunotherapy. Nat Rev Immunol.

[303] Hotchkiss RS, Monneret G, Payen D (2013). Immunosuppression in sepsis: a novel understanding of the disorder and a new therapeutic approach. Lancet Infect Dis.

[304] Ward NS, Casserly B, Ayala A (2008). The compensatory anti-inflammatory response syndrome (CARS) in critically ill patients. Clin Chest Med.

[305] Bone RC (1996). Sir Isaac Newton, sepsis, SIRS, and CARS. Crit Care Med.

[306] Musuuza JS, Watson L, Parmasad V, Putman-Buehler N, Christensen L, Safdar N (2021). Prevalence and outcomes of co-infection and superinfection with SARS-CoV-2 and other pathogens: a systematic review and meta-analysis. PLoS One.

[307] Hori S, Carvalho TL, Demengeot J (2002) CD25+CD4+ regulatory T cells suppress CD4+ T cell-mediated pulmonary hyperinflammation driven by Pneumocystis carinii in immunodeficient mice. Eur J Immunol 32(5):1282–1291. 10.1002/1521-4141(200205)32:5<1282::AID-IMMU1282>3.0.CO;2-# 11981815

[308] Chen G, Wu D, Guo W, Cao Y, Huang D, Wang H, Wang T, Zhang X, Chen H, Yu H, Zhang X, Zhang M, Wu S, Song J, Chen T, Han M, Li S, Luo X, Zhao J, Ning Q (2020). Clinical and immunological features of severe and moderate coronavirus disease 2019. J Clin Invest.

[309] Sadeghi A, Tahmasebi S, Mahmood A, Kuznetsova M, Valizadeh H, Taghizadieh A, Nazemiyeh M, Aghebati-Maleki L, Jadidi-Niaragh F, Abbaspour-Aghdam S, Roshangar L, Mikaeili H, Ahmadi M (2020) Th17 and Treg cells function in SARS-CoV2 patients compared with healthy controls. J Cell Physiol. 10.1002/jcp.30047 10.1002/jcp.30047 32926425

[310] Wang F, Hou H, Luo Y, Tang G, Wu S, Huang M, Liu W, Zhu Y, Lin Q, Mao L, Fang M, Zhang H, Sun Z (2020) The laboratory tests and host immunity of COVID-19 patients with different severity of illness. JCI Insight 5(10). 10.1172/jci.insight.137799 10.1172/jci.insight.137799 PMC7259533 32324595

[311] Meckiff BJ, Ramírez-Suástegui C, Fajardo V, Chee SJ, Kusnadi A, Simon H, Eschweiler S, Grifoni A, Pelosi E, Weiskopf D, Sette A, Ay F, Seumois G, Ottensmeier CH, Vijayanand P (2020) Imbalance of regulatory and cytotoxic SARS-CoV-2-reactive CD4(+) T cells in COVID-19. Cell. 10.1016/j.cell.2020.10.001 10.1016/j.cell.2020.10.001 PMC7534589 33096020

[312] Wu X, Ren J, Chen G, Wu L, Song X, Li G, Deng Y, Wang G, Gu G, Li J (2017). Systemic blockade of P2X7 receptor protects against sepsis-induced intestinal barrier disruption. Sci Rep.

[313] Wang S, Zhao J, Wang H, Liang Y, Yang N, Huang Y (2015). Blockage of P2X7 attenuates acute lung injury in mice by inhibiting NLRP3 inflammasome. Int Immunopharmacol.

[314] Galam L, Rajan A, Failla A, Soundararajan R, Lockey RF, Kolliputi N (2016). Deletion of P2X7 attenuates hyperoxia-induced acute lung injury via inflammasome suppression. Am J Phys Lung Cell Mol Phys.

[315] Pacheco PAF, Faria RX (2021). The potential involvement of P2X7 receptor in COVID-19 pathogenesis: a new therapeutic target?. Scand J Immunol.

[316] Ribeiro DE, Oliveira-Giacomelli Á, Glaser T, Arnaud-Sampaio VF, Andrejew R, Dieckmann L, Baranova J, Lameu C, Ratajczak MZ, Ulrich H (2021). Hyperactivation of P2X7 receptors as a culprit of COVID-19 neuropathology. Mol Psychiatry.

[317] Wang HL, Xing YQ, Xu YX, Rong F, Lei WF, Zhang WH (2013). The protective effect of lidocaine on septic rats via the inhibition of high mobility group box 1 expression and NF-κB activation. Mediat Inflamm.

[318] Liu J, Zhang H, Qi Z, Zheng X (2014). Lidocaine protects against renal and hepatic dysfunction in septic rats via downregulation of Toll-like receptor 4. Mol Med Rep.

[319] Gallos G, Jones DR, Nasr SH, Emala CW, Lee HT (2004). Local anesthetics reduce mortality and protect against renal and hepatic dysfunction in murine septic peritonitis. Anesthesiology.

[320] Berger C, Rossaint J, Van Aken H, Westphal M, Hahnenkamp K, Zarbock A (2014). Lidocaine reduces neutrophil recruitment by abolishing chemokine-induced arrest and transendothelial migration in septic patients. J Immunol.

[321] Barnes BJ, Adrover JM, Baxter-Stoltzfus A, Borczuk A, Cools-Lartigue J, Crawford JM, Daßler-Plenker J, Guerci P, Huynh C, Knight JS, Loda M, Looney MR, McAllister F, Rayes R, Renaud S, Rousseau S, Salvatore S, Schwartz RE, Spicer JD et al (2020) Targeting potential drivers of COVID-19: neutrophil extracellular traps. J Exp Med 217(6). 10.1084/jem.20200652 10.1084/jem.20200652 PMC7161085 32302401

[322] Narasaraju T, Tang BM, Herrmann M, Muller S, Chow VTK, Radic M (2020). Neutrophilia and NETopathy as key pathologic drivers of progressive lung impairment in patients with COVID-19. Front Pharmacol.

[323] Middleton EA, He XY, Denorme F, Campbell RA, Ng D, Salvatore SP, Mostyka M, Baxter-Stoltzfus A, Borczuk AC, Loda M, Cody MJ, Manne BK, Portier I, Harris ES, Petrey AC, Beswick EJ, Caulin AF, Iovino A, Abegglen LM, Weyrich AS, Rondina MT, Egeblad M, Schiffman JD, Yost CC (2020). Neutrophil extracellular traps contribute to immunothrombosis in COVID-19 acute respiratory distress syndrome. Blood.

[324] Tomar B, Anders HJ, Desai J, Mulay SR (2020) Neutrophils and neutrophil extracellular traps drive necroinflammation in COVID-19. Cells 9(6). 10.3390/cells9061383 10.3390/cells9061383 PMC7348784 32498376

[325] Li H, Li C, Zhang H, Zhang L, Cheng R, Li M, Guo Y, Zhang Z, Lu Z, Zhuang Y, Yan M, Gu Y, Feng X, Liang J, Yu X, Wang H, Yao Z (2016). Effects of lidocaine on regulatory T cells in atopic dermatitis. J Allergy Clin Immunol.

[326] Van Der Wal S, Vaneker M, Steegers M, Van Berkum B, Kox M, Van Der Laak J, Van Der Hoeven J, Vissers K, Scheffer GJ (2015). Lidocaine increases the anti-inflammatory cytokine IL-10 following mechanical ventilation in healthy mice. Acta Anaesthesiol Scand.

[327] Garutti I, Rancan L, Simón C, Cusati G, Sanchez-Pedrosa G, Moraga F, Olmedilla L, Lopez-Gil MT, Vara E (2014). Intravenous lidocaine decreases tumor necrosis factor alpha expression both locally and systemically in pigs undergoing lung resection surgery. Anesth Analg.

[328] Hermanns H, Hollmann MW, Stevens MF, Lirk P, Brandenburger T, Piegeler T, Werdehausen R (2019). Molecular mechanisms of action of systemic lidocaine in acute and chronic pain: a narrative review. Br J Anaesth.

[329] Weinberg L, Peake B, Tan C, Nikfarjam M (2015). Pharmacokinetics and pharmacodynamics of lignocaine: a review. World J Anesthesiol.

[330] Montnach J, Lorenzini M, Lesage A, Simon I, Nicolas S, Moreau E, Marionneau C, Baró I, De Waard M, Loussouarn G (2021). Computer modeling of whole-cell voltage-clamp analyses to delineate guidelines for good practice of manual and automated patch-clamp. Sci Rep.

[331] Wang Y, Jones PJ, Batts TW, Landry V, Patel MK, Brown ML (2009). Ligand-based design and synthesis of novel sodium channel blockers from a combined phenytoin-lidocaine pharmacophore. Bioorg Med Chem.

[332] Cheng KI, Lai CS, Wang FY, Wang HC, Chang LL, Ho ST, Tsai HP, Kwan AL (2011). Intrathecal lidocaine pretreatment attenuates immediate neuropathic pain by modulating Nav1.3 expression and decreasing spinal microglial activation. BMC Neurol.

[333] Gingrich KJ, Wagner LE (2016). Fast-onset lidocaine block of rat NaV1.4 channels suggests involvement of a second high-affinity open state. Biochim Biophys Acta.

[334] Elajnaf T, Baptista-Hon DT, Hales TG (2018). Potent inactivation-dependent inhibition of adult and neonatal NaV1.5 channels by lidocaine and levobupivacaine. Anesth Analg.

[335] Sheets PL, Jarecki BW, Cummins TR (2011). Lidocaine reduces the transition to slow inactivation in Na(v)1.7 voltage-gated sodium channels. Br J Pharmacol.

[336] Mert T, Gunes Y (2012). Antinociceptive activities of lidocaine and the nav1.8 blocker a803467 in diabetic rats. J Am Assoc Lab Anim Sci.

[337] Dusmez D, Cengiz B, Yumrutas O, Demir T, Oztuzcu S, Demiryurek S, Tutar E, Bayraktar R, Bulut A, Simsek H, Daglı SN, Kılıc T, Bagcı C (2014). Effect of verapamil and lidocaine on TRPM and NaV1.9 gene expressions in renal ischemia-reperfusion. Transplant Proc.

[338] Wang L, Wang M, Li S, Wu H, Shen Q, Zhang S, Fang L, Liu R (2018). Nebulized lidocaine ameliorates allergic airway inflammation via downregulation of TLR2. Mol Immunol.

[339] Nishizawa N, Shirasaki T, Nakao S, Matsuda H, Shingu K (2002). The inhibition of the N-methyl-D-aspartate receptor channel by local anesthetics in mouse CA1 pyramidal neurons. Anesth Analg.

[340] Bennett DL, Clark AJ, Huang J, Waxman SG, Dib-Hajj SD (2019). The role of voltage-gated sodium channels in pain signaling. Physiol Rev.

[341] Kis-Toth K, Hajdu P, Bacskai I, Szilagyi O, Papp F, Szanto A, Posta E, Gogolak P, Panyi G, Rajnavolgyi E (2011). Voltage-gated sodium channel Nav1.7 maintains the membrane potential and regulates the activation and chemokine-induced migration of a monocyte-derived dendritic cell subset. J Immunol.

[342] Carrithers LM, Hulseberg P, Sandor M, Carrithers MD (2011). The human macrophage sodium channel NaV1.5 regulates mycobacteria processing through organelle polarization and localized calcium oscillations. FEMS Immunol Med Microbiol.

[343] Black JA, Waxman SG (2012). Sodium channels and microglial function. Exp Neurol.

[344] Poffers M, Bühne N, Herzog C, Thorenz A, Chen R, Güler F, Hage A, Leffler A, Echtermeyer F (2018). Sodium channel Nav1.3 is expressed by polymorphonuclear neutrophils during mouse heart and kidney ischemia in vivo and regulates adhesion, transmigration, and chemotaxis of human and mouse neutrophils in vitro. Anesthesiology.

[345] Lo WL, Donermeyer DL, Allen PM (2012). A voltage-gated sodium channel is essential for the positive selection of CD4(+) T cells. Nat Immunol.

[346] Lee JY, Kam YL, Oh J, Kim DH, Choi JS, Choi HY, Han S, Youn I, Choo HP, Yune TY (2017). HYP-17, a novel voltage-gated sodium channel blocker, relieves inflammatory and neuropathic pain in rats. Pharmacol Biochem Behav.

[347] Jarvis MF, Honore P, Shieh CC, Chapman M, Joshi S, Zhang XF, Kort M, Carroll W, Marron B, Atkinson R, Thomas J, Liu D, Krambis M, Liu Y, McGaraughty S, Chu K, Roeloffs R, Zhong C, Mikusa JP, Hernandez G, Gauvin D, Wade C, Zhu C, Pai M, Scanio M, Shi L, Drizin I, Gregg R, Matulenko M, Hakeem A, Gross M, Johnson M, Marsh K, Wagoner PK, Sullivan JP, Faltynek CR, Krafte DS (2007). A-803467, a potent and selective Nav1.8 sodium channel blocker, attenuates neuropathic and inflammatory pain in the rat. Proc Natl Acad Sci U S A.

[348] Joshi SK, Honore P, Hernandez G, Schmidt R, Gomtsyan A, Scanio M, Kort M, Jarvis MF (2009). Additive antinociceptive effects of the selective Nav1.8 blocker A-803467 and selective TRPV1 antagonists in rat inflammatory and neuropathic pain models. J Pain.

[349] Bankar G, Goodchild SJ, Howard S, Nelkenbrecher K, Waldbrook M, Dourado M, Shuart NG, Lin S, Young C, Xie Z, Khakh K, Chang E, Sojo LE, Lindgren A, Chowdhury S, Decker S, Grimwood M, Andrez JC, Dehnhardt CM, Pang J, Chang JH, Safina BS, Sutherlin DP, Johnson JP, Hackos DH, Robinette CL, Cohen CJ (2018). Selective Na(V)1.7 Antagonists with long residence time show improved efficacy against inflammatory and neuropathic pain. Cell Rep.

[350] Sun H, Jiang J, Gong L, Li X, Yang Y, Luo Y, Guo Z, Lu R, Li H, Li J, Zhao J, Yang N, Li Y (2019). Voltage-gated sodium channel inhibitor reduces atherosclerosis by modulating monocyte/macrophage subsets and suppressing macrophage proliferation. Biomed Pharmacother.

[351] Beloeil H, Ababneh Z, Chung R, Zurakowski D, Mulkern RV, Berde CB (2006). Effects of bupivacaine and tetrodotoxin on carrageenan-induced hind paw inflammation in rats (Part 1): hyperalgesia, edema, and systemic cytokines. Anesthesiology.

[352] Beloeil H, Ji RR, Berde CB (2006). Effects of bupivacaine and tetrodotoxin on carrageenan-induced hind paw inflammation in rats (Part 2): cytokines and p38 mitogen-activated protein kinases in dorsal root ganglia and spinal cord. Anesthesiology.

[353] Alcántara Montero A, Sánchez Carnerero CI (2021). Voltage-gated sodium channel blockers: new perspectives in the treatment of neuropathic pain. Neurologia.

[354] Huang C, Wang Y, Li X, Ren L, Zhao J, Hu Y, Zhang L, Fan G, Xu J, Gu X, Cheng Z, Yu T, Xia J, Wei Y, Wu W, Xie X, Yin W, Li H, Liu M, Xiao Y, Gao H, Guo L, Xie J, Wang G, Jiang R, Gao Z, Jin Q, Wang J, Cao B (2020). Clinical features of patients infected with 2019 novel coronavirus in Wuhan, China. Lancet.

[355] Dhar SK, Damodar S, Gujar S, Das M, K V (2021). IL-6 and IL-10 as predictors of disease severity in COVID-19 patients: results from meta-analysis and regression. Heliyon.

[356] Udomsinprasert W, Jittikoon J, Sangroongruangsri S, Chaikledkaew U (2021). Circulating levels of interleukin-6 and Interleukin-10, but not tumor necrosis factor-alpha, as potential biomarkers of severity and mortality for COVID-19: systematic review with meta-analysis. J Clin Immunol.

[357] Sun D, Li H, Lu XX, Xiao H, Ren J, Zhang FR, Liu ZS (2020) Clinical features of severe pediatric patients with coronavirus disease 2019 in Wuhan: a single center's observational study. World J Pediatr. 10.1007/s12519-020-00354-4 10.1007/s12519-020-00354-4 PMC7091225 32193831

[358] Zhou C, Qi C, Zhao J, Wang F, Zhang W, Li C, Jing J, Kang X, Chai Z (2011). Interleukin-1β inhibits voltage-gated sodium currents in a time- and dose-dependent manner in cortical neurons. Neurochem Res.

[359] Li X, Chen W, Sheng J, Cao D, Wang W (2014). Interleukin-6 inhibits voltage-gated sodium channel activity of cultured rat spinal cord neurons. Acta Neuropsychiatr.

[360] Shen KF, Zhu HQ, Wei XH, Wang J, Li YY, Pang RP, Liu XG (2013). Interleukin-10 down-regulates voltage gated sodium channels in rat dorsal root ganglion neurons. Exp Neurol.

[361] Del Puerto A, Fronzaroli-Molinieres L, Perez-Alvarez MJ, Giraud P, Carlier E, Wandosell F, Debanne D, Garrido JJ (2015). ATP-P2X7 receptor modulates axon initial segment composition and function in physiological conditions and brain injury. Cereb Cortex.

[362] Huang C, Chi XS, Li R, Hu X, Xu HX, Li JM, Zhou D (2017). Inhibition of P2X7 receptor ameliorates nuclear factor-kappa B mediated neuroinflammation induced by status epilepticus in rat hippocampus. J Mol Neurosci.

[363] Liu Y, Xiao Y, Li Z (2011). P2X7 receptor positively regulates MyD88-dependent NF-κB activation. Cytokine.

[364] Petrenko AB, Yamakura T, Baba H, Shimoji K (2003). The role of N-methyl-D-aspartate(NMDA) receptors in pain: a review. Anesth Analg.

[365] Di Loreto S, Balestrino M, Pellegrini P, Berghella AM, Del Beato T, Di Marco F, Adorno D (1997). Blockade of N-methyl-D-aspartate receptor prevents hypoxic neuronal death and cytokine release. Neuroimmunomodulation.

[366] Érces D, Varga G, Fazekas B, Kovács T, Tőkés T, Tiszlavicz L, Fülöp F, Vécsei L, Boros M, Kaszaki J (2012). N-methyl-D-aspartate receptor antagonist therapy suppresses colon motility and inflammatory activation six days after the onset of experimental colitis in rats. Eur J Pharmacol.

[367] Liu CH, Cherng CH, Lin SL, Yeh CC, Wu CT, Tai YH, Wong CS (2011). N-methyl-D-aspartate receptor antagonist MK-801 suppresses glial pro-inflammatory cytokine expression in morphine-tolerant rats. Pharmacol Biochem Behav.

[368] Simma N, Bose T, Kahlfuss S, Mankiewicz J, Lowinus T, Lühder F, Schüler T, Schraven B, Heine M, Bommhardt U (2014). NMDA-receptor antagonists block B-cell function but foster IL-10 production in BCR/CD40-activated B cells. Cell Commun Signal.

[369] Sugimoto M, Uchida I, Mashimo T (2003). Local anaesthetics have different mechanisms and sites of action at the recombinant N-methyl-D-aspartate(NMDA) receptors. Br J Pharmacol.

[370] Hahnenkamp K, Durieux ME, Hahnenkamp A, Schauerte SK, Hoenemann CW, Vegh V, Theilmeier G, Hollmann MW (2006). Local anaesthetics inhibit signalling of human NMDA receptors recombinantly expressed in Xenopus laevis oocytes: role of protein kinase C. Br J Anaesth.

[371] Kahlfuß S, Simma N, Mankiewicz J, Bose T, Lowinus T, Klein-Hessling S, Sprengel R, Schraven B, Heine M, Bommhardt U (2014). Immunosuppression by N-methyl-D-aspartate receptor antagonists is mediated through inhibition of Kv1.3 and KCa3.1 channels in T cells. Mol Cell Biol.

[372] Hajimohammadreza I, Probert AW, Coughenour LL, Borosky SA, Marcoux FW, Boxer PA, Wang KK (1995). A specific inhibitor of calcium/calmodulin-dependent protein kinase-II provides neuroprotection against NMDA- and hypoxia/hypoglycemia-induced cell death. J Neurosci.

[373] Cotrina ML, Nedergaard M (2009). Physiological and pathological functions of P2X7 receptor in the spinal cord. Purinergic Signal.

[374] Alves LA, Bezerra RJ, Faria RX, Ferreira LG, da Silva FV (2013). Physiological roles and potential therapeutic applications of the P2X7 receptor in inflammation and pain. Molecules.

[375] Hunter MC, Teijeira A, Halin C (2016). T cell trafficking through lymphatic vessels. Front Immunol.

[376] Teijeira A, Russo E, Halin C (2014). Taking the lymphatic route: dendritic cell migration to draining lymph nodes. Semin Immunopathol.

[377] Lund H, Boysen P, Åkesson CP, Lewandowska-Sabat AM, Storset AK (2016). Transient migration of large numbers of CD14(++) CD16(+) monocytes to the draining lymph node after onset of inflammation. Front Immunol.

[378] Louie DAP, Liao S (2019). Lymph node subcapsular sinus macrophages as the frontline of lymphatic immune defense. Front Immunol.

[379] Hampton HR, Chtanova T (2016). The lymph node neutrophil. Semin Immunol.

[380] Wang HW, Tedla N, Lloyd AR, Wakefield D, McNeil PH (1998). Mast cell activation and migration to lymph nodes during induction of an immune response in mice. J Clin Invest.

[381] Shi HZ, Humbles A, Gerard C, Jin Z, Weller PF (2000). Lymph node trafficking and antigen presentation by endobronchial eosinophils. J Clin Invest.

[382] Kim S, Prout M, Ramshaw H, Lopez AF, LeGros G, Min B (2010). Cutting edge: basophils are transiently recruited into the draining lymph nodes during helminth infection via IL-3, but infection-induced Th2 immunity can develop without basophil lymph node recruitment or IL-3. J Immunol.

[383] Tse D, Stan RV (2010). Morphological heterogeneity of endothelium. Semin Thromb Hemost.

[384] Kretsos K, Kasting GB (2005). Dermal capillary clearance: physiology and modeling. Skin Pharmacol Physiol.

[385] Breslin JW, Yang Y, Scallan JP, Sweat RS, Adderley SP, Murfee WL (2018). Lymphatic vessel network structure and physiology. Compr Physiol.

[386] Kersey TW, Van Eyk J, Lannin DR, Chua AN, Tafra L (2001). Comparison of intradermal and subcutaneous injections in lymphatic mapping. J Surg Res.

[387] Karimeddini MK, Spencer RP (1989). Intradermal pathways. New procedures in nuclear medicine.

[388] Thomasy SM, Pypendop BH, Ilkiw JE, Stanley SD (2005). Pharmacokinetics of lidocaine and its active metabolite, monoethylglycinexylidide, after intravenous administration of lidocaine to awake and isoflurane-anesthetized cats. Am J Vet Res.

[389] Hatef DA, Brown SA, Lipschitz AH, Kenkel JM (2009). Efficacy of lidocaine for pain control in subcutaneous infiltration during liposuction. Aesthet Surg J.

[390] Wu F, Tamhane M, Morris ME (2012). Pharmacokinetics, lymph node uptake, and mechanistic PK model of near-infrared dye-labeled bevacizumab after IV and SC administration in mice. AAPS J.

[391] Dahlberg AM, Kaminskas LM, Smith A, Nicolazzo JA, Porter CJ, Bulitta JB, McIntosh MP (2014). The lymphatic system plays a major role in the intravenous and subcutaneous pharmacokinetics of trastuzumab in rats. Mol Pharm.

[392] Worley DR, Hansen RJ, Wittenburg LA, Chubb LS, Gustafson DL (2016). Docetaxel accumulates in lymphatic circulation following subcutaneous delivery compared to intravenous delivery in rats. Anticancer Res.

[393] Stock TC, Bloom BJ, Wei N, Ishaq S, Park W, Wang X, Gupta P, Mebus CA (2012). Efficacy and safety of CE-224,535, an antagonist of P2X7 receptor, in treatment of patients with rheumatoid arthritis inadequately controlled by methotrexate. J Rheumatol.

[394] Keystone EC, Wang MM, Layton M, Hollis S, McInnes IB (2012). Clinical evaluation of the efficacy of the P2X7 purinergic receptor antagonist AZD9056 on the signs and symptoms of rheumatoid arthritis in patients with active disease despite treatment with methotrexate or sulphasalazine. Ann Rheum Dis.

[395] Bhattacharya A (2018). Recent advances in CNS P2X7 physiology and pharmacology: focus on neuropsychiatric disorders. Front Pharmacol.

[396] Timmers M, Ravenstijn P, Xi L, Triana-Baltzer G, Furey M, Van Hemelryck S, Biewenga J, Ceusters M, Bhattacharya A, van den Boer M, van Nueten L, de Boer P (2018). Clinical pharmacokinetics, pharmacodynamics, safety, and tolerability of JNJ-54175446, a brain permeable P2X7 antagonist, in a randomised single-ascending dose study in healthy participants. J Psychopharmacol.

[397] Eser A, Colombel JF, Rutgeerts P, Vermeire S, Vogelsang H, Braddock M, Persson T, Reinisch W (2015). Safety and efficacy of an oral inhibitor of the purinergic receptor P2X7 in adult patients with moderately to severely active Crohn's disease: a randomized placebo-controlled, double-blind, phase iia study. Inflamm Bowel Dis.

[398] Shakweh M, Ponchel G, Fattal E (2004). Particle uptake by Peyer's patches: a pathway for drug and vaccine delivery. Expert Opin Drug Deliv.

[399] Zgair A, Wong JCM, Gershkovich P (2016) Targeting immunomodulatory agents to the gut-associated lymphoid tissue. Neuro-Immuno-Gastroenterology:237–261. 10.1007/978-3-319-28609-9_14

[400] Trevaskis NL, Charman WN, Porter CJ (2008). Lipid-based delivery systems and intestinal lymphatic drug transport: a mechanistic update. Adv Drug Deliv Rev.

[401] Trevaskis NL, Charman WN, Porter CJ (2010). Targeted drug delivery to lymphocytes: a route to site-specific immunomodulation?. Mol Pharm.

[402] Hsu YW, Somma J, Newman MF, Mathew JP (2011). Population pharmacokinetics of lidocaine administered during and after cardiac surgery. J Cardiothorac Vasc Anesth.

[403] Mori K, Ito H, Toda Y, Hashimoto T, Miyazaki M, Saijo T, Kuroda Y (2004). Successful management of intractable epilepsy with lidocaine tapes and continuous subcutaneous lidocaine infusion. Epilepsia.

[404] Ranieri VM, Rubenfeld GD, Thompson BT, Ferguson ND, Caldwell E, Fan E, Camporota L, Slutsky AS (2012). Acute respiratory distress syndrome: the Berlin Definition. Jama.

[405] Wang J, Zheng P, Huang Z, Huang H, Xue M, Liao C, Sun B, Zhong N (2020). Serum SP-A and KL-6 levels can predict the improvement and deterioration of patients with interstitial pneumonia with autoimmune features. BMC Pulm Med.

[406] Mallapaty S (2020). The coronavirus is most deadly if you are older and male - new data reveal the risks. Nature.

[407] Izcovich A, Ragusa MA, Tortosa F, Lavena Marzio MA, Agnoletti C, Bengolea A, Ceirano A, Espinosa F, Saavedra E, Sanguine V, Tassara A, Cid C, Catalano HN, Agarwal A, Foroutan F, Rada G (2020). Prognostic factors for severity and mortality in patients infected with COVID-19: A systematic review. PLoS One.

[408] Diaz-Vera M, Terrones Santa Cruz J, Forttini Headrington A, Cerna Paz JA, Quintanilla Rios L, Medina Melendez MP, Del Águila Torres J (2020) Lidocaine to reduce the severity of covid-19 cases. Therapia Neural. Sabadell. Barcelona, Spain. http://www.terapianeural.com/articulos/28-studies-estudios/495-lidocaine-to-reduce-the-severity-of-covid-19-cases. Accessed 19-11 2020

[409] Muller M, Lefebvre F, Harlay ML, Glady L, Becker G, Muller C, Aberkane O, Tawk M, Julians M, Romoli A, Hecketsweiler S, Schneider F, Pottecher J, Chamaraux-Tran TN (2021). Impact of intravenous lidocaine on clinical outcomes of patients with ARDS during COVID-19 pandemia (LidoCovid): a structured summary of a study protocol for a randomised controlled trial. Trials.

[410] Bharat A, Querrey M, Markov NS, Kim S, Kurihara C, Garza-Castillon R, Manerikar A, Shilatifard A, Tomic R, Politanska Y, Abdala-Valencia H, Yeldandi AV, Lomasney JW, Misharin AV, Budinger GRS (2020) Lung transplantation for patients with severe COVID-19. Sci Transl Med. 10.1126/scitranslmed.abe4282 10.1126/scitranslmed.abe4282 PMC8050952 33257409

[411] Gordh T (2010). Lidocaine: the origin of a modern local anesthetic. 1949. Anesthesiology.

[412] Derry S, Wiffen PJ, Moore RA, Quinlan J (2014). Topical lidocaine for neuropathic pain in adults. Cochrane Database Syst Rev.

[413] Beaussier M, Delbos A, Maurice-Szamburski A, Ecoffey C, Mercadal L (2018). Perioperative use of intravenous lidocaine. Drugs.

[414] Su D, Gu Y, Wang Z, Wang X (2010). Lidocaine attenuates proinflammatory cytokine production induced by extracellular adenosine triphosphate in cultured rat microglia. Anesth Analg.

[415] Yuan T, Li Z, Li X, Yu G, Wang N, Yang X (2014). Lidocaine attenuates lipopolysaccharide-induced inflammatory responses in microglia. J Surg Res.

[416] Flondor M, Listle H, Kemming GI, Zwissler B, Hofstetter C (2010). Effect of inhaled and intravenous lidocaine on inflammatory reaction in endotoxaemic rats. Eur J Anaesthesiol.

[417] Chen LJ, Ding YB, Ma PL, Jiang SH, Li KZ, Li AZ, Li MC, Shi CX, Du J, Zhou HD (2018). The protective effect of lidocaine on lipopolysaccharide-induced acute lung injury in rats through NF-κB and p38 MAPK signaling pathway and excessive inflammatory responses. Eur Rev Med Pharmacol Sci.

[418] Feng G, Liu S, Wang G-L, Liu G-J (2008). Lidocaine attenuates lipopolysaccharide-induced acute lung injury through inhibiting NF-kappaB activation. Pharmacology.

[419] Huang TK, Uyehara CF, Balaraman V, Miyasato CY, Person D, Egan E, Easa D (2004). Surfactant lavage with lidocaine improves pulmonary function in piglets after HCl-induced acute lung injury. Lung.

[420] Kiyonari Y, Nishina K, Mikawa K, Maekawa N, Obara H (2000). Lidocaine attenuates acute lung injury induced by a combination of phospholipase A2 and trypsin. Crit Care Med.

[421] Pedersen JL, Callesen T, Møiniche S, Kehlet H (1996). Analgesic and anti-inflammatory effects of lignocaine-prilocaine(EMLA) cream in human burn injury. Br J Anaesth.

[422] Werner RM, Hoffman AK, Coe NB (2020). Long-term care policy after Covid-19 - solving the nursing home crisis. N Engl J Med.

[423] Lesch CA, Squier CA, Cruchley A, Williams DM, Speight P (1989). The permeability of human oral mucosa and skin to water. J Dent Res.

[424] Goyal AK, Singh R, Chauhan G, Rath G (2018). Non-invasive systemic drug delivery through mucosal routes. Artif Cells Nanomed Biotechnol.

[425] Gröningsson K, Lindgren JE, Lundberg E, Sandberg R, Wahlén A (1985) Lidocaine base and hydrochloride. In: Florey K (ed) Analytical profiles of drug substances, vol 14. Academic Press, pp 207-243. 10.1016/S0099-5428(08)60582-1

[426] Charman WNA, Stella VJ (1986). Estimating the maximal potential for intestinal lymphatic transport of lipophilic drug molecules. Int J Pharm.

[427] Porter CJ, Trevaskis NL, Charman WN (2007). Lipids and lipid-based formulations: optimizing the oral delivery of lipophilic drugs. Nat Rev Drug Discov.

[428] Gvetadze SR, Ilkaev KD (2020). Lingual lymph nodes: anatomy, clinical considerations, and oncological significance. World J Clin Oncol.

[429] Ananian SG, Gvetadze SR, Ilkaev KD, Mochalnikova VV, Zayratiants GO, Mkhitarov VA, Yang X, Ciciashvili AM (2015). Anatomic-histologic study of the floor of the mouth: the lingual lymph nodes. Jpn J Clin Oncol.

[430] Fujimura A, Sato Y, Shoji M, Onodera M, Nozaka Y (2007). Lymphatic architecture of the oral region - beneath the buccal mucosa. Microvasc Rev Commun.

[431] Ossoff RH, Sisson GA (1981). Lymphatics of the floor of the mouth and neck: anatomical studies related to contralateral drainage pathways. Laryngoscope.

[432] Mohammadi-Samani S, Jamshidzadeh A, Montaseri H, Rangbar-Zahedani M, Kianrad R (2010). The effects of some permeability enhancers on the percutaneous absorption of lidocaine. Pak J Pharm Sci.

[433] Sakdiset P, Kitao Y, Todo H, Sugibayashi K (2017). High-throughput screening of potential skin penetration-enhancers using stratum corneum lipid liposomes: preliminary evaluation for different concentrations of ethanol. J Pharm (Cairo).

[434] Ali Khan A, Mudassir J, Mohtar N, Darwis Y (2013). Advanced drug delivery to the lymphatic system: lipid-based nanoformulations. Int J Nanomedicine.

[435] Cho HY, Lee YB (2014). Nano-sized drug delivery systems for lymphatic delivery. J Nanosci Nanotechnol.

[436] Zhang XY, Lu WY (2014). Recent advances in lymphatic targeted drug delivery system for tumor metastasis. Cancer Biol Med.

[437] Ahn H, Park JH (2016). Liposomal delivery systems for intestinal lymphatic drug transport. Biomater Res.

